# CALM Regulates Clathrin-Coated Vesicle Size and Maturation by Directly Sensing and Driving Membrane Curvature

**DOI:** 10.1016/j.devcel.2015.03.002

**Published:** 2015-04-20

**Authors:** Sharon E. Miller, Signe Mathiasen, Nicholas A. Bright, Fabienne Pierre, Bernard T. Kelly, Nikolay Kladt, Astrid Schauss, Christien J. Merrifield, Dimitrios Stamou, Stefan Höning, David J. Owen

**Affiliations:** 1 Cambridge Institute for Medical Research and Department of Clinical Biochemistry, University of Cambridge, Cambridge Biomedical Campus, Wellcome Trust/MRC Building, Hills Road, Cambridge CB2 0XY, UK; 2 Bionanotechnology and Nanomedicine Laboratory, Department of Chemistry, University of Copenhagen, Universitetsparken 5, 2100 Copenhagen, Denmark; 3 Laboratoire d’Enzymologie et Biochimie Structurales, UPR3082 CNRS - Bat 34, Avenue de la Terrasse, 91198 Gif-sur-Yvette, France; 4 Cologne Excellence Cluster on Cellular Stress Responses in Aging-Associated Diseases (CECAD), Joseph-Stelzmann-Str. 26, 50931 Cologne, Germany; 5 Institute of Biochemistry I and Center for Molecular Medicine Cologne, University of Cologne, Joseph-Stelzmann-Str. 52, 50931 Cologne, Germany

## Abstract

The size of endocytic clathrin-coated vesicles (CCVs) is remarkably uniform, suggesting that it is optimized to achieve the appropriate levels of cargo and lipid internalization. The three most abundant proteins in mammalian endocytic CCVs are clathrin and the two cargo-selecting, clathrin adaptors, CALM and AP2. Here we demonstrate that depletion of CALM causes a substantial increase in the ratio of “open” clathrin-coated pits (CCPs) to “necked”/“closed” CCVs and a doubling of CCP/CCV diameter, whereas AP2 depletion has opposite effects. Depletion of either adaptor, however, significantly inhibits endocytosis of transferrin and epidermal growth factor. The phenotypic effects of CALM depletion can be rescued by re-expression of wild-type CALM, but not with CALM that lacks a functional N-terminal, membrane-inserting, curvature-sensing/driving amphipathic helix, the existence and properties of which are demonstrated. CALM is thus a major factor in controlling CCV size and maturation and hence in determining the rates of endocytic cargo uptake.

## Highlights

• CALM loss increases size and frequency of early endocytic clathrin-coated structures
• Depletion of CALM slows endocytic clathrin-coated pit maturation and endocytic rate
• CALM possesses an N-terminal, membrane-curvature-sensing/driving amphipathic helix
• Clathrin-coated pit maturation is regulated by CALM’s N-terminal amphipathic helix

## Graphical Abstract

**Figure:**
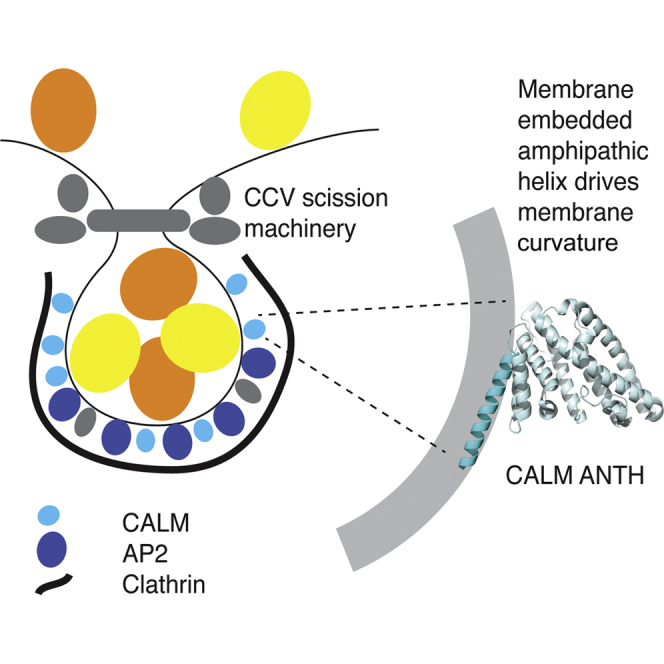

## Teaser

Miller et al. demonstrate that depletion of the abundant endocytic protein CALM increases the diameter and percentage of early endocytic clathrin-coated structures (CCSs) while delaying CCS maturation and reducing endocytic rates. CALM’s ability to influence these CCS properties depends on a membrane-inserting amphipathic helix, which senses and promotes membrane curvature.

## Introduction

During clathrin-mediated endocytosis (CME), clathrin-coated pits (CCPs) are most frequently initiated by the formation of a small patch of the heterotetrameric AP2 clathrin adaptor complex and clathrin at sites of high PtdIns4,5P_2_ concentration (Cocucci et al., 2012; Traub, 2011). If not aborted due to a lack of sufficient PtdIns4,5P_2_ and/or cargo (Loerke et al., 2009), local membrane curvature steadily increases as more clathrin and a variety of additional clathrin adaptors are recruited until a bulbous membrane structure of ∼80–100 nm diameter is formed on top of a membrane stalk that can undergo scission to generate a clathrin-coated vesicle (CCV) (Traub, 2011). The most abundant clathrin adaptors in endocytic CCVs isolated from tissue culture cells are CALM (clathrin assembly lymphoid myeloid leukemia protein) and AP2, each of which account for 30%–35% of the adaptors in a CCV (Blondeau et al., 2004; Borner et al., 2012). AP2 binds to and sorts general, often large, transmembrane cargo such as the transferrin receptor (TfR) into CCVs. CALM binds to and sorts the small R-SNAREs VAMPs 2, 3, and 8 (Koo et al., 2011; Miller et al., 2011). CALM and its neuronal specific homolog AP180, possess large, natively unstructured, C-terminal tails containing clathrin, AP2 α- and β2-appendage binding sites (Ford et al., 2001; Tebar et al., 1999; Traub, 2011), and a membrane-proximal, PtdIns4,5P_2_-binding N-terminal ANTH stacked-helical domain, defined as residues 19–289 (Ford et al., 2001).

Several studies have shown that depletion of CALM in tissue culture cells and vertebrate neurons or of its orthologs in *D. melanogaster* and *C. elegans* causes an increase in CCP/CCV size and probably also a decrease in their uniformity of shape (Bao et al., 2005; Meyerholz et al., 2005; Nonet et al., 1999; Petralia et al., 2013; Zhang et al., 1998). These morphological alterations most likely result from changes in membrane curvature/sculpting. However, structural investigations had shown that CALM possessed neither a BAR domain nor an amphipathic helix that could influence membrane curvature, which was consistent with CALM apparently not affecting membrane curvature in biophysical assays (Boucrot et al., 2012; Ford et al., 2002; Stahelin et al., 2003). In an attempt to reconcile these contradictory observations of a key cellular process, we set out to determine whether, and if so, how CALM could directly affect CCP/CCV size and thus the cargo-carrying capacity because these are two key features in understanding the mechanism of endocytic CCP/CCV formation. Here we show that CALM possesses an N-terminal amphipathic helix that regulates CCP/CCV size and maturation and hence endocytic rate.

## Results

### Effect of CALM Depletion In Vivo

**Figure 1:**
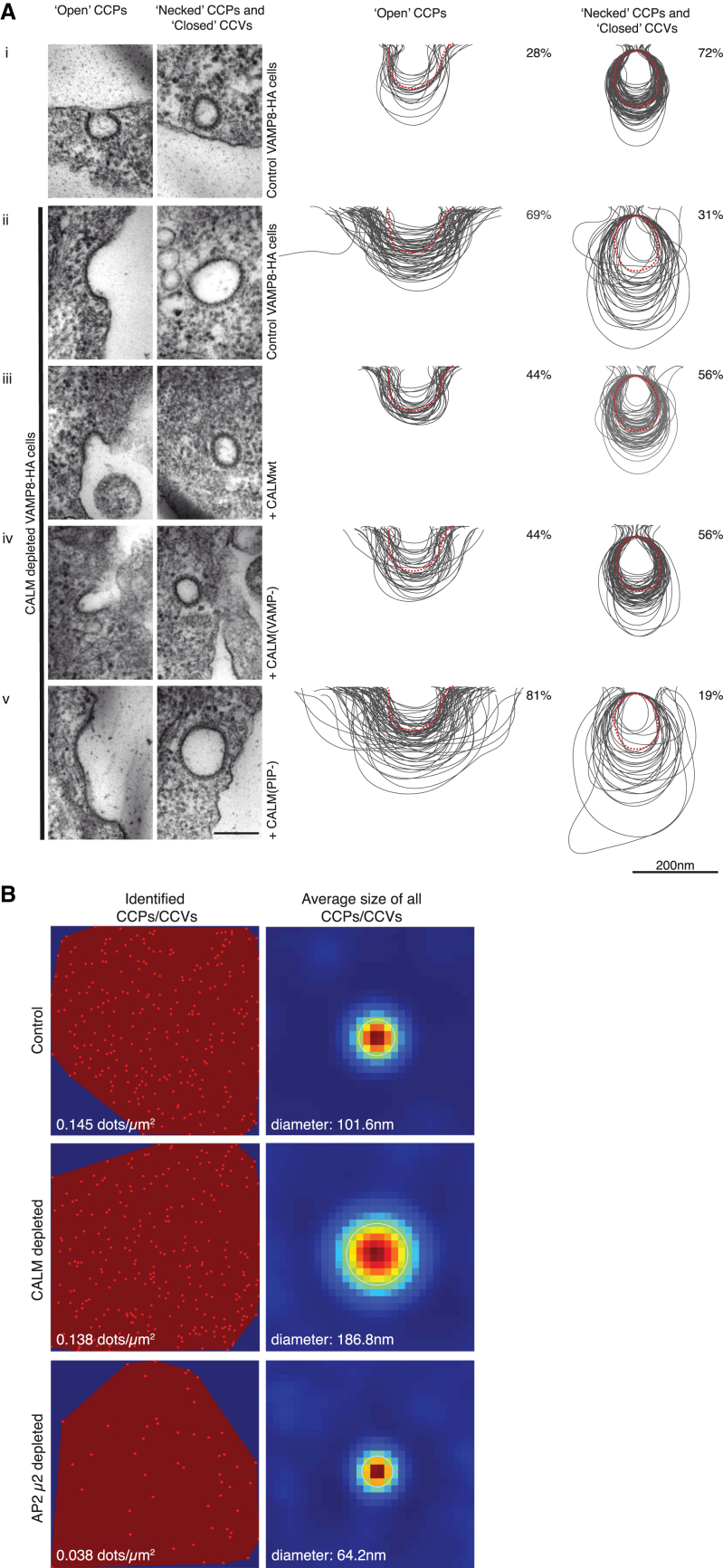
CALM Is Important for CCP and CCV Size Morphology (A) All cells express an HA-tagged version of the endocytic SNARE VAMP8. The size variation of clathrin-coated structures, as a consequence of CALM depletion without and with re-expression of WT and mutant versions of CALM (ii–iv), compared to those found in control cells (i) are shown and quantified. Left-hand columns show representative images of open and necked/closed clathrin-coated structures for each cell line. Right-hand columns show the traces of 100 randomly selected clathrin-coated structures and the percentage (∼150 images) of each type of structure adjacent to the traces. Dotted red line represents average size in control cells. Scale bars represent 200 nm. (B) Cells were stained with antibodies for clathrin, AP2, and CALM and appropriate fluorochrome-conjugated secondary antibodies. After acquisition of confocal images, STED images were collected for clathin and processed for quantification, as described in Experimental Procedures. Images on the left display all identified clathrin-stained CCPs/CCVs in a polygonal area defined by the outermost objects and the calculated number/μm^2^. Images on the right show a heat map of the overlay of all CCPs/CCVs identified (image on the left) and the calculated average diameter.

**Figure 2:**
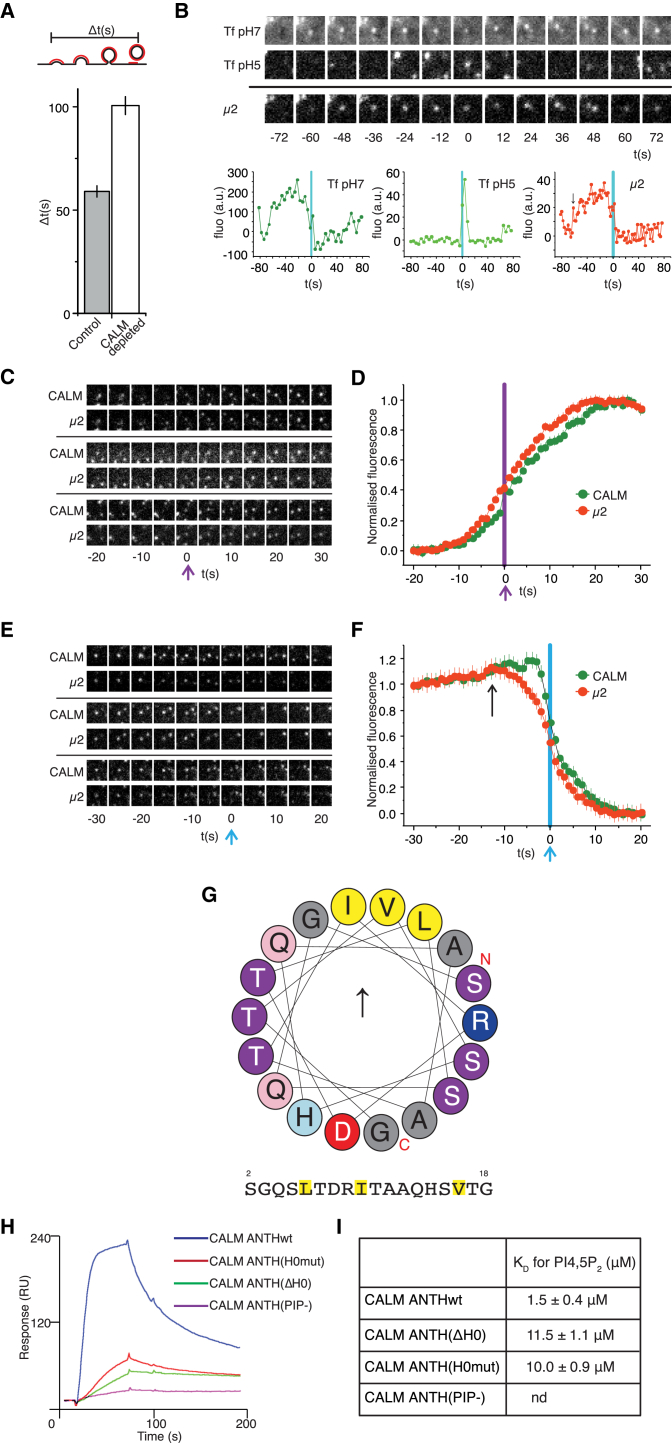
CALM Affects CCV Maturation and Possesses an Amphipathic Helix that Contributes to Membrane Binding (A) The time between first detection of a CCP and the first detected scission event at that CCP (Δts) was significantly extended in cells treated with CALM siRNA (control cells, Δt = 59 s, SEM = 3 s, 688 events, five cells; CALM siRNA, Δt = 100 s, SEM = 4 s, 590 events, five cells). (B) An example CCP and scission event from a control cell. Images were acquired using TIRF microscopy and the pulsed pH protocol to detect single scission events. Images acquired at pH 7 (upper, Tf pH7) show a cluster of Transferrin-phluorin (Tf-phl) at a CCP. A scission event was detected when Tf-phl at the cluster became insulated from the externally imposed pH change (middle, Tf pH5, t = 0 s). Simultaneous detection of μ2-mCherry showed this CCP formed ∼62 s before the detected scission event (lower, arrow in μ2-mCherry graph). The quantified fluorescence changes for this example CCP are plotted (graphs). (C) Montages of example clathrin-coated pit nucleation events imaged using TIRF microscopy. The purple arrow indicates t = 0 s, the first frame in which the CALM-GFP (green) object was detected using automated tracking. (D) Average CALM-GFP and μ2-mCherry (red) fluorescence traces of nucleating CCPs aligned to the first frame of detection of CALM-GFP (green). The average traces were normalized between the average of the first three fluorescence values and the peak fluorescence value for CALM and μ2, respectively. The fluorescence traces have a characteristic sigmoidal shape. (E) Montages of example clathrin-coated pit budding events. The blue arrow indicates t = 0 s, the moment of maximum decrease in CALM-GFP fluorescence that corresponds to vesicle scission. (F) Average CALM-GFP and μ2-mCherry at budding clathrin-coated pits. The average fluorescence traces were normalized to the average of the first three and last three fluorescence values for CALM and μ2, respectively. The μ2-mCherry fluorescence starts to decrease ∼15 s before the CALM-GFP signal (black arrow). (G) Heliquest server (http://heliquest.ipmc.cnrs.fr) helical wheel shows the orientation of the key hydrophobic residues (in yellow) and predicts the N terminus of CALM is an amphipathic helix with curvature-sensing properties. The hydrophobic moment points up toward the membrane and polar but mainly uncharged side chains are disposed laterally along where the surface the membrane would be. The negative charge points down away from the membrane. (H) Liposome-based SPR sensorgrams showing binding of CALM ANTH-WT, CALM ANTH(ΔH0), CALM ANTH(H0mut), and CALM ANTH(PIP−) to PtdIns4,5P_2_-containing liposomes at a protein concentration of 6 μM. (I) K_D_ values for the binding of CALM ANTH domains, determined by liposome-based SPR (mean and SD of four independent measurements). Representative sensorgrams used for the measurements are shown in Figure S2A.

Endogenous CALM was depleted by siRNA and subsequent quantitated ultrastructural studies by electron microscopy (EM) and stimulated-emission depletion (STED) microscopy showed that CALM depletion results in substantially enlarged CCPs and CCVs compared to that of control cells (Figures 1, S1A, S1C, and S1E; see also Meyerholz et al., 2005), but no discernable change in the number of clathrin-coated structures (Figures 1B and S1A). The CCPs/CCVs in control cells have an average diameter of ∼90 nm (Heuser, 1980). This value increased by ∼2-fold upon depletion of CALM producing an ∼4-fold increase in surface area, hence an ∼8-fold increase in volume (Figures 1, S1C, and S1E). Importantly, our quantitation of CCP/CCV profiles of EM micrographs showed that “necked” CCPs/“closed” CCVs were the predominant fraction in control cells (∼70%), whereas the shallow “open” CCPs accounted for only ∼30% (Figures 1A and S1D). This was reversed in CALM-depleted cells, strongly suggestive of an alteration in the efficiency of CCP/CCV maturation, likely resulting from a reduced ability to curve/sculpt the plasma membrane (Figures 1A and S1D). In agreement with this, total internal reflection fluorescence (TIRF) microscopy revealed that the time taken to proceed from initiation of a CCP to its scission approximately doubles when CALM is depleted (Figures 2A and 2B).

We then compared the effects of expressing endogenous levels of full-length, siRNA-resistant CALM-wild-type (WT), CALM(PIP−), a mutant which has no PtdIns4,5P_2_ binding due to the mutations Lys28Glu, Lys38Glu, and Lys40Glu (Ford et al., 2001) and is therefore cytosolic or CALM(VAMP−), which has no R-SNARE binding due to the mutation Met244Lys (Miller et al., 2011) in conjunction with endogenous CALM depletion (Figures S1A and S1B). Immunofluorescence indicated no obvious reduction in the number of punctate endocytic CCPs/CCVs (Figure S1A), whereas EM analysis of the CCP/CCV profiles showed that the expression of CALM-WT or CALM(VAMP−) rescued the relative ratios of open versus closed and sizes of CCPs/CCVs, whereas re-expression of CALM(PIP−) did not (Figures 1A and S1D). These data therefore indicate that CALM is not a major factor in CCP initiation, but rather that its presence strongly affects aspects of early to middle stages of CCP/CCV formation, and consequently influences the final CCV size. Because both CALM-WT and CALM(VAMP−) rescue phenotypes similarly, we further suggest that CALM’s SNARE binding is not a checkpoint during CCP/CCV formation.

To substantiate our findings, we assessed the relative dynamics of AP2 and CALM during CCP formation in living cells with TIRF microscopy. These data showed that AP2 levels begin to decrease 15–20 s prior to the sharp decrease in the CALM levels that corresponds to the point when CCV scission occurs (Figures 2C–2F; see Cocucci et al., 2012; Loerke et al., 2009; Taylor et al., 2011; Traub, 2011). Within these 20 s prior scission, net membrane curvature must still increase to produce a necked and bulbous structure; hence, it is the level of CALM and not that of AP2 that correlates best with the increasing amount of positive curvature during CCP/CCV formation. CALM’s binding partners FCHO, NECAP, and clathrin (Ritter et al., 2013; Tebar et al., 1999; Umasankar et al., 2012) also bind AP2 and so should be present in CCPs even when CALM is absent. This suggests that CALM’s effects on CCP/CCV formation are not indirect through the action of these binding partners, but are consistent with a direct effect of CALM itself.

### The N-Terminal 18 Residues of CALM Form an Amphipathic Helix

The impact of CALM on the size and shape of CCPs/CCVs led us to re-examine the structure of CALM to determine how it could directly affect membrane curvature. Inspection of the N-terminal residues 1–18 of CALM with the Heliquest server (http://heliquest.ipmc.cnrs.fr) suggested that this region could, in fact, assume an amphipathic helix (AH) (Figure 2G). Membrane-interacting AHs adopt their helical conformations when they attach to phospholipid membranes (Bhatia et al., 2009; Drin et al., 2007; Gallop et al., 2006); and indeed, liposome-based surface plasmon resonance (SPR) measurements showed that this potential N-terminal AH of CALM (we now term residues 1–18 as AH0, to be consistent with previous literature on other clathrin adaptors) contributed to the membrane binding of CALM. CALM ANTH bound to PtdIns4,5P_2_ liposomes at physiological salt concentration (170 mM) with a K_D_ of 1.5 μM, whereas deletion of AH0 (CALM ANTH[ΔH0]), or the mutation of the key hydrophobic AH0 residues Leu6, Ile10, and Val17 to serines (CALM ANTH[H0mut]) both led to a decrease in affinity by ∼8-fold (Figures 2H, 2I, and S2A). Furthermore, in the presence of PtdIns4,5P_2_-containing liposomes, WT CALM ANTH showed a small but reproducible increase in α-helical content by circular dichroism (CD) (Figure S2B).

**Figure 3:**
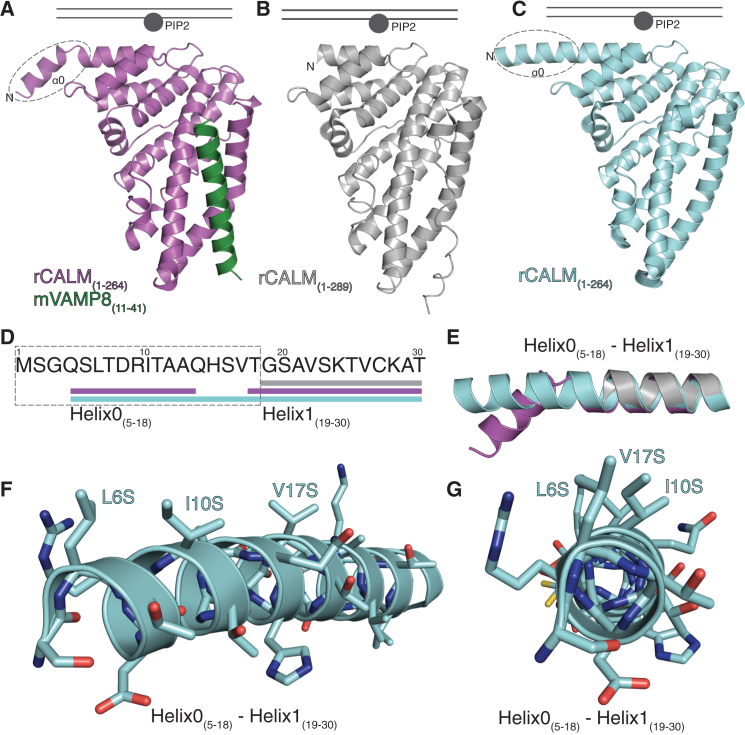
Structure of the CALM N-Terminal Amphipathic Helix (AH0) CALM ANTH domain is in gray, truncated CALM ANTH_(1–264)_ in pale blue, and the CALM ANTH:VAMP8 complex in purple/green. (A–C) Three different structures of the first 289 residues of CALM with the binding site for PtdIns4,5P_2_ and the proposed position of the membrane indicated. AH0 is indicated with a dashed ellipse. (D and E) Positions (D) and ribbon representation (E) of α helices in the N-terminal 30 residues of the various structures. AH0 is indicated with a dashed rectangle. (F and G) Angled (F) and face on (G) ribbon representations of the N terminus of CALM ANTH_(1–264)_, highlighting the positions of the key AH0 hydrophobic residues Leu6, Ile10, and Val17 mutated to serines.

Comparison of our CALM ANTH:VAMP8 complex structure (Miller et al., 2011) with previous structures of the human and *Drosophila* CALM ANTH domains (Ford et al., 2001; Mao et al., 2001; Miller et al., 2011) showed that residues 5–14 can indeed form a short N-terminal helix that is connected to helix1 by a linker with the central axes of the helices diverging by ∼40° at residues 14–17 (Figures 3A, 3E, S3A, and S3B). The structure of a C-terminally truncated form of the ANTH domain of CALM (CALM ANTH_(1–264)_) (Table S1; Figures 3C, S3A, and S3C) although lacking the SNARE binding site, again showed residues 5–18 form a helix but this time extending helix1 by an additional four turns (Figures 3C, 3E, S3A, and S3C). The increased stabilizing energy that would arise from the continued α-helical H-bonding pattern seen in the structure of CALM ANTH_(1–264)_ suggests that this extension of helix1 is the most likely conformation for residues 5–18 when the helix formation is induced by either membrane insertion or by fortuitous crystal packing. The failure to detect the existence and effects of this helix, in previous in vitro studies, likely results from this region’s high proteolytic sensitivity.

### AH0 of CALM Displays Membrane Curvature Sensing and Drives Membrane Deformation In Vitro

In other peripheral membrane proteins such as ArfGAP1, membrane-interacting amphipathic helices show membrane curvature sensing (MCS) and are referred to as ALPS (amphipathic lipid packing sensing) helices (Antonny, 2011; Bigay et al., 2003; Drin et al., 2007; Hatzakis et al., 2009). MCS arises from a combination of physicochemical effects including the insertion of the hydrophobic residue side chains of the helix into curvature-induced defects in membrane lipid packing and the interactions between the hydrophilic amino acid side chains of the helix and membrane phospholipid headgroups (Jensen et al., 2011).

**Figure 4:**
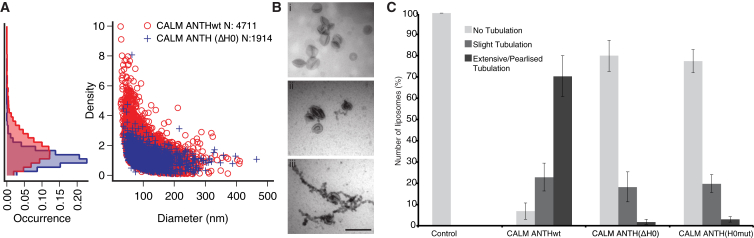
The AH0 of the CALM ANTH Domain Senses Membrane Curvature and Promotes Membrane Deformation (A) Single liposome membrane curvature sensing assay showing CALM ANTHwt and CALM ANTH(**Δ**H0) density versus liposome diameter for PtdIns4,5P_2_ containing liposomes. Protein concentration = 500 nM. The marginal histograms (left) reveal the occurrence of protein densities for CALM ANTH-WT and CALM ANTH(ΔH0). (B) Tubulation of PtdIns4,5P_2_-containing liposomes by 0.5 μM CALM ANTH proteins for 1 min. Sample electron micrographs showing (Bi) no, (Bii) slight, and (Biii) extensive pearlized tubulation typically seen. Scale bar represents 500 nm. (C) CALM ANTH-WT causes extensive tubulation in ∼70% of micrographs, whereas CALM ANTH(ΔH0) CALM ANTH(H0mut) do so only in ∼2% and ∼3%, respectively. Error bars show the SEM.

When used in a sensitive microscope-based MCS assay on individual fluorescent liposomes (Lohr et al., 2009), CALM ANTH showed a preference for binding to small liposomes <100 nm diameter (around the size of closing CCPs), demonstrated by the enlarged population of smaller liposomes with higher protein densities (Figures 4A and S4A–S4G). CALM ANTH(ΔH0) showed a significantly reduced ability to sense curvature (Figures 4A and S4A–S4G), with a tendency to populate markedly lower protein densities. Taken together, our in vitro data indicate that CALM indeed possesses MCS capability, which is largely mediated by its AH0.

The deformation/tubulation of liposomes is often used as an indicator of membrane sculpting activity in vitro (Itoh et al., 2005; Peter et al., 2004; Stachowiak et al., 2012; van Weering et al., 2012). CALM ANTH-WT at 0.5 μM was extremely efficient at extensively tubulating 200 nm PtdIns4,5P_2_-containing liposomes within 1 minute (Figures 4B, 4C, S4H, and S4I). CALM ANTH(ΔH0) and CALM ANTH(H0mut) at 0.5 μM were largely unable to deform the liposomes despite binding to them (Figure 4C). At concentrations of <0.1 μM, CALM ANTH-WT was unable to tubulate liposomes. The observed tubulation was not, however, reminiscent of the straight-sided tubules produced by BAR domains (Ford et al., 2002; Itoh et al., 2005; Peter et al., 2004; van Weering et al., 2012). It was instead “pearlized” (Stachowiak et al., 2012), with the pearls having approximately the same diameter (40–50 nm) (Figures 4B, S4H, and S4I) as the smallest endocytic CCVs found in neuronal synapses, which are enriched for CALM and AP180 (Blondeau et al., 2004). In previous liposome tubulation assays, CALM ANTH was used at concentrations of 4–20 μM (Ford et al., 2002; Stachowiak et al., 2012). At the concentration of CALM used here, we calculate a resulting surface coverage of ∼16% (see Supplemental Experimental Procedures), a value close to that calculated for epsin, which forms smaller (17 nm diameter) tubules from liposomes (Kozlov et al., 2014). The value for CALM surface coverage in vitro resembles our estimate of its coverage in vivo of an endocytic CCV’s surface area of ∼17%. Our calculation (see Supplemental Experimental Procedures) shows the value for CALM will be much greater than that for epsin because it has been estimated that epsin constitutes less than 1% of clathrin adaptor content in endocytic CCVs (Borner et al., 2012). We conclude that the ANTH domain of CALM can drive pearlized liposome tubulation using its AH0.

We propose, therefore, that in vivo the targeting of CALM into a forming CCP through its preferential association with curved membranes should have two major effects: CALM will stabilize existing membrane curvature (by effectively shifting the thermodynamic equilibrium in favor of curved structures (Baumgart et al., 2011) and will also actively drive further membrane deformation/curvature toward that of endocytic CCVs (a diameter of ∼90 nm, which corresponds to CALM’s preferred binding curvature). Although we cannot definitively prove insertion of an unmodified AH0 of CALM into a membrane, the biochemical and biophysical properties of CALM’s AH0 are consistent with it undergoing membrane insertion, as is accepted for similar AHs in other proteins (Antonny, 2011; Boucrot et al., 2012; Kozlov et al., 2014). This role for CALM is also consistent with its recruitment profile (Figures 2C–2F and Taylor et al., 2011) and as such would also help to concentrate CALM-binding endocytic R-SNAREs in CCVs.

In addition to helix insertion or wedging the other main contributor to protein driven membrane curvature comes from molecular crowding i.e., the lateral pressure exerted by colliding proteins that are attached on one side of a membrane (Boucrot et al., 2012; Kozlov et al., 2014; Stachowiak et al., 2012). The degree of membrane curvature driven by molecular crowding depends on protein size, concentration, and membrane affinity. CALM, due to its ∼70 kDa molecular weight in combination with its >300 residue C-terminal unstructured region, should therefore contribute significantly to driving membrane curvature. The AH0 of CALM would also affect molecular crowding effects because AH0 not only increases the general membrane affinity of CALM, but also preferentially targets CALM to and thus concentrates it on the forming curved CCPs. CALM can thus initiate a positive amplification cascade of increasing membrane curvature starting from its initially low level in very early i.e., recently initiated CCPs to the final value in a CCV (corresponding to ∼90 nm diameter) through multiple functions of its AH0. Taken together with the observation that CALM is highly abundant in CCVs (30%–35% of the clathrin adaptors (Borner et al., 2012)), these data allow us to propose that the AH0 of CALM controls the efficiency of endocytic CCP/CCV maturation and size by both sensing and driving membrane deformation.

### CALM’s AH0 Plays a Critical Role in Endocytic CCP Formation In Vivo

**Figure 5:**
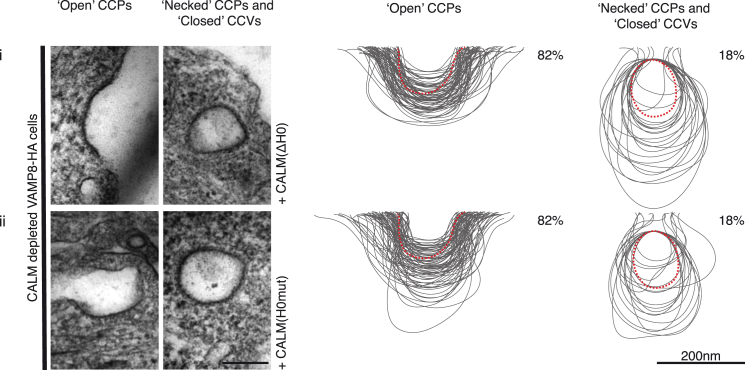
CALM with a Non-Functional AH0 Cannot Rescue CALM-Depleted CCP/CCV Profile Phenotypes The size variation of clathrin-coated structures, as a consequence of CALM depletion with re-expression of mutant versions of (i) CALM(ΔH0) and (ii) CALM(H0mut) compared to those found in control cells as indicated by the dotted red line. Left-hand columns show representative images of open and necked/closed clathrin-coated structures for each cell line. Right-hand columns show the traces of 100 randomly selected clathrin-coated structures and the percentage (∼150 images) of each type of structure adjacent to the traces. Scale bars represent 200 nm.

To confirm this hypothesis, we examined the effect of deleting AH0 or mutating AH0 on the ability of CALM to influence CCP/CCV properties in vivo by expressing endogenous levels of full-length, siRNA-resistant CALM(ΔH0) or CALM(H0mut) following endogenous CALM depletion (Figure S1B). Immunofluorescence indicated no obvious reduction in the number of punctate endocytic CCPs/CCVs (Figure S5A). However, despite CALM(ΔH0) or CALM(H0mut) being present in CCPs/CCVs and able to mediate normal trafficking of VAMP8 (Figure S5A), EM analysis showed that CCP/CCV morphology and the ratio of open to necked/closed structures resembled that of cells depleted of CALM (Figures 1A, 5, S1C, and S1D). That is, expression of CALM(ΔH0) or CALM(H0mut) failed to rescue the enlarged size phenotype and the normal ratio of open versus necked/closed clathrin-coated structures.

### CALM Requires a Functional AH0 to Rescue the Reduction in Endocytic Rate Caused by CALM Depletion

**Figure 6:**
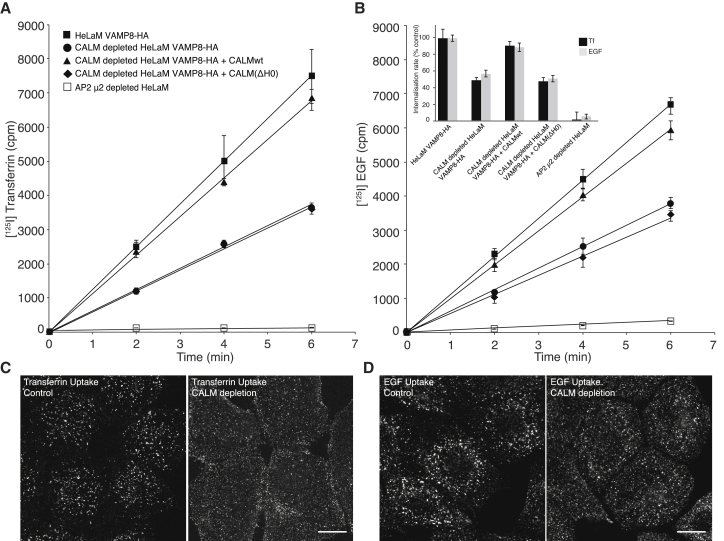
CALM’s AH0 Directly Affects Rates of Endocytosis (A and B) Internalization of (A) [^125^I] transferrin (Tf) and (B) [^125^I] EGF in HeLaM VAMP8-HA-expressing cells were measured for 2, 4, and 6 minutes. The amount of surface bound and internalized radioactivity was quantified and plotted against time. Each experiment was performed at least twice with triplicates for each time point. Error bars represent the SD of triplicates. Cells depleted of CALM showed an ∼50% reduction in endocytosis of both Tf and EGF compared to that of control cells. This endocytic defect is rescued with the expression of siRNA resistant CALMwt, but not with CALM(ΔH0). Cells depleted of AP2 showed an ∼90% reduction in endocytosis of both Tf and EGF compared to that of control cells. The bar chart (insert) shows a comparison of the internalization rate constants (k_e_), which represent the linear regression coefficients. Those for the internalization by control HeLaM VAMP8-HA cells were set to 100%; all other values were put in relation to it. Error bars represent the SD. (C and D) Immunofluorescence of cells after 6 min endocytosis of Tf-Alexa568 (C) or EGF-Alexa488 (D) by VAMP8-HA control and VAMP8-HA CALM-depleted cells. The images are maximum projections of ten focal z planes covering the whole volume of the imaged cells. Scale bar represents 15 μm.

In light of the major changes in CCP:CCV ratio, the time taken for CCP maturation and CCP/CCV size, we investigated the effect of CALM depletion on receptor/ligand internalization rates by measuring the steady state uptake rates at 37°C of radio-iodinated Tf and EGF. As shown in Figures 6A and 6B, CALM depletion caused ∼50% reduction of both Tf and EGF uptake. This endocytosis defect was restored to normal in cells expressing siRNA-resistant CALM-WT, but not rescued in cells expressing CALM(ΔH0). Immunofluorescence correspondingly showed intracellular accumulation of Tf and EGF in control cells after 6 min endocytosis, whereas both ligands remain in punctae associated with the plasma membrane in cells depleted of endogenous CALM (Figures 6C and 6D). We thus conclude that the absence of CALM that possesses a functional AH0 delays CCV maturation (Figures 2A and 2B), resulting in a significant endocytic defect.

### Depletion of AP2 and CALM Produces Opposite Effects on CCP/CCV Size In Vivo

**Figure 7:**
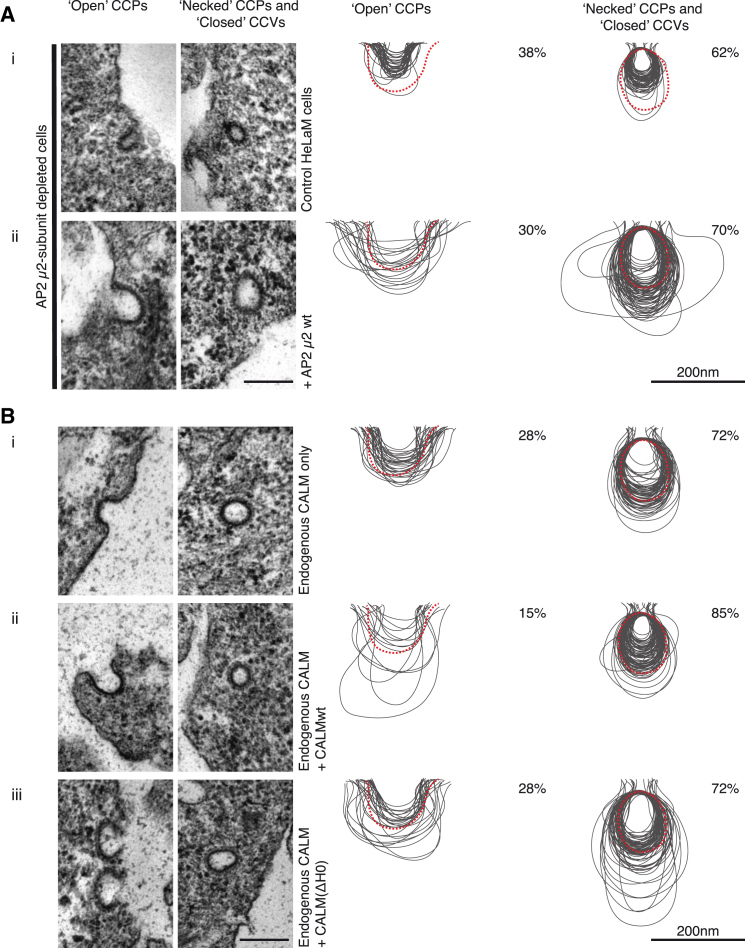
AP2 Depletion and CALM Overexpression Alter CCP/CCV Profile Phenotypes Electron micrographs (left-hand columns) show a typical representation of an open and a necked/closed clathrin-coated structure for each cell line. Traces (right-hand columns) were made from 100 images and counts represented in percentages are of ∼150 images for each condition. Dotted red line represents average size of CCPs/CCVs in control cells. (A) The size variation of clathrin-coated structures are shown, and quantified AP2 (μ2 subunit)-depleted cells (Ai) show an obvious reduction in CCP/CCV size that can be rescued with the expression of an siRNA-resistant AP2-μ2 subunit (Aii). (B) The size variation of clathrin-coated structures as a consequence of expressing siRNA-resistant CALM variants in addition to endogenous CALM (without siRNA treatment) (Bii and Biii). The levels of CALM were approximately double that in control cells as shown by western blotting (Figure S1). Attempts to create cell lines expressing higher levels of CALM expression failed presumably because higher levels of CALM expression are toxic. (Bi) As before, control cells show a greater percentage of necked/closed clathrin-coated structures (∼70%) than open structures, and all profiles are within the size range previously obtained. Clathrin-coated structures in cells expressing endogenous and siRNA-resistant CALM-WT (Bii) were noticeably smaller than profiles of control profiles (note the size of the central cavity) (Bi) or of cells expressing endogenous and siRNA-resistant CALM(ΔH0) (Biii). In (Bii), there is also an increase in the percentage of necked/closed structures (85% as opposed to ∼70% for Bi and Biii). Scale bars represent 200 nm.

CALM and AP2 depletion both caused a major reduction in Tf and in EGF internalization rates (Figures 6A and 6B; see also Huang et al., 2004). EM analysis however, showed in marked contrast to CALM depletion, AP2 depletion (of μ2) resulted in an ∼12-fold decrease in the number of endocytic CCPs/CCVs (see also Motley et al., 2003) and a striking ∼2-fold reduction in the diameter of the remaining clathrin-coated endocytic structures (note, this reduction in diameter is equivalent to a reduction in volume by ∼90%) (Figures 1B, 7A, S1C–S1E). These AP2 depletion phenotypes were completely rescued by restoring the AP2 levels with re-expression of siRNA-resistant μ2 (Figure 7A). The clathrin adaptor complement of AP2-depleted endocytic structures should be dominated by CALM (the major PtdIns4,5P_2_-binding endocytic adaptor remaining), suggesting that increasing amounts of CALM-WT in CCPs/CCVs results in them being very small (Figures 1B, 7A, and S1C). Unfortunately, we have found it impossible to accurately quantify the relative amount of CALM and clathrin in AP2-depleted cells because the number and size of endocytic CCPs/CCVs was so drastically reduced. In support of our proposal that the absence one of the two major clathrin adaptors causes the other one to dominate, quantification of STED images showed that depletion of CALM leads to a 2- to 3-fold increase in the AP2:clathrin ratio in clathrin-coated endocytic structures (data not shown).

In agreement with the proposal that CALM drives increasing membrane curvature, we also find that increased CALM expression (that is, endogenous CALM levels combined with similar levels of expression of siRNA-resistant CALM-WT) reduced CCP/CCV size and caused an increase in the number of necked/closed structures (Figures 7B, S1C, and S1D), whereas a combination of endogenous CALM and expression of CALM(ΔH0) expression did not (Figures 7B, S1C, and S1D). Unfortunately, it was not possible to create clonal cell lines stably expressing any higher levels of CALM (probably due to toxicity through inhibition of CME; Tebar et al., 1999) or to overexpress AP2 (which would require simultaneous overexpression of all four subunits). In conclusion, we propose that in vivo despite both being important for clathrin-mediated endocytosis, AP2 and CALM have antagonistic effects on CCP/CCV size and the ratio of open versus necked/closed structures, hence their relative levels are a major determinant in defining various CCP/CCV properties.

## Discussion

The initiation of CCP formation is generally believed to most often involve a pioneer complex of AP2/FCHO/Eps15 being recruited to a region of elevated PtdIns4,5P_2_ (Cocucci et al., 2012; Hollopeter et al., 2014; Traub, 2011; Umasankar et al., 2014), which is then stabilized on the membrane by AP2 cargo being drawn in (Kelly et al., 2014; Loerke et al., 2009) and clathrin recruited and polymerized. Addition of more clathrin adaptors, including CALM, and of clathrin itself drives maturation of the CCP. Because CALM preferentially binds to curved membranes and CCP shape is a dynamic/equilibrium system, an increased amount of CALM in a CCP would tend to shift the net steady state of the structure toward increased membrane curvature; that is, CALM effectively drives membrane curvature. The increasing CCP curvature will result in CALM having an increased affinity for that region of the membrane and thus more CALM will be recruited to it i.e., a positive amplification cascade is established. Positive membrane curvature will increase until the CCP can be readily necked and then undergoes scission. The necked structure would likely resemble a single pearl such as those we see spontaneously produced on PtdIns4,5P_2_-containing liposomes. The increase in the number of open CCPs and concomitant enlargement both resulting from the absence of fully functional CALM suggests a defect in the formation of late stage necked, bulbous CCPs. This defect would most likely be caused by the increased difficulty in necking a larger open CCP. Hence, it will take longer to neck/close the resulting larger CCP under these, suggesting that in the absence of CALM, CCP necking/scission tends toward being rate limiting. This proposal is supported by the observation that depletion of CALM caused a doubling of the time taken to reach a point at which a neck is formed that is small enough to allow vesicle scission to occur. That the AH0 of CALM plays a principle role in controlling of CCP/CCV size, curvature, and maturation can be inferred from the observation that neither expression of CALM(ΔH0) nor of CALM(H0mut) rescued the enlarged CCP/CCV phenotype, the CCV formation efficiency and the reduced endocytic rate. Intriguingly a similar increase in CCP size is seen when AP2 is replaced by AP2 lacking its α-appendage, the domain responsible for concentrating many CME proteins, including CALM, in CCPs (Aguet et al., 2013). These data suggest that a failure to recruit enough CALM into these AP2 positive CCPs could be the explanation for this phenotype.

Why should the depletion of CALM or AP2 have opposite effects on CCP/CCV morphology? Why should the presence of larger CCPs/CCVs in CALM depleted cells with little reduction in the number of CCPs/CCVs reduce overall endocytic rate? We believe that both issues are linked to CALM’s and AP2′s different structural/biophysical properties and the type of cargo they recognize. First, CALM possesses a curvature sensing/driving AH0 whereas AP2, as far as we know, does not. Second, the transmembrane cargoes bound by AP2 often have large extracellular portions (including TfR, EGFR) that, upon collision, will generate lateral pressure (Scheve et al., 2013; Stachowiak et al., 2012) causing membrane curvature in the opposite direction to that of inward budding favored by adaptor-mediated molecular crowding on the membrane’s cytoplasmic face. This is analogous to a model put forward to explain the effect of GPI-linked cargo in COPII-coated vesicle formation (Copic et al., 2012). In a normal CCV, CALM will oppose this effect and so drive CCV closure. However, in endocytic CCPs where CALM has been depleted and therefore AP2 is predominant, there will be a need for a reduction in large cargo molecule density to reduce cargo collisions and so allow CCP closure. This can be achieved if no more cargo packed into these larger CCPs than is packed into ordinary sized CCPs. That this occurs is suggested by the similar and even slightly reduced intensity of TfR staining of the diffraction limited spots at the surface of control and CALM-depleted cells shown in Figure 6. However, it should also be noted that if the situation is such that in control cells most TfR is in internal compartments and any TfR delivered to the plasma membrane is immediately incorporated into CCPs as suggested (Hopkins et al., 1985), then larger CCPs resulting from CALM depletion cannot take up any more TfR than CCPs in control cells because no more TfR can be available to be taken up. In this case, endocytic rate will be determined by the rate at which the CCPs mature.

In contrast, the VAMP cargo of CALM has a negligible extracellular/luminal portion that does not oppose the positive membrane curvature driven by adaptor molecular crowding and the effect of CALM AH0 insertion. Thus, when CALM is the predominant adaptor (i.e., AP2 is depleted), the result is greatly reduced CCV size exacerbated by the fact that there is no large luminal domain cargo (AP2 binding) actively sorted into these structures to oppose CCP positive membrane curvature.

As CCP curvature approaches its final value and the bud neck forms, to avoid steric clashing/molecular crowding on the luminal side of the membrane, large YxxΦ and ExxxLL motif containing cargoes and their bound AP2 will tend to be excluded from this area and concentrate in the crown of the bulb (Saffarian and Kirchhausen, 2008). This would explain why AP2 levels appear to decline prior to scission when analyzed by TIRF microscopy (Aguet et al., 2013; Rappoport et al., 2003; Taylor et al., 2011). As a result of its sensing and driving positive membrane curvature and it not binding cargo that is excluded from the neck region of a CCP, CALM should continue to be recruited to the curved CCP membrane and should also not be partitioned into the crown. Hence, CALM’s levels appear to continue to increase until scission. CALM may actually also aid in removing AP2 from near the neck as in the absence of AP2 cargo that has been excluded for steric reasons but with the positive curvature preferred by CALM, CALM should be able to more effectively compete with AP2 for binding of PtdIns4,5P_2_ in this region (Honing et al., 2005).

Because an alteration in the clathrin adaptor complement alters the size and curvature of a CCP/CCV, we conclude that the presence of clathrin adaptors with the ability to drive membrane curvature by both insertion/wedging and molecular crowding plays a major role in defining CCP/CCV morphology. However, we cannot say from our studies what the contributions of membrane composition (Pinot et al., 2014) and the clathrin lattice (Dannhauser and Ungewickell, 2012; Heuser, 1980; Kelly et al., 2014; Meyerholz et al., 2005) are in driving and/or stabilizing CCP/CCV membrane curvature.

The coats of other transport vesicles contain and are attached to the membrane via small GTPases: Sar1:GTP (in COPII vesicles) or Arf1:GTP (in other CCVs and COPI vesicles), both of which possess AHs that display MCS, drive membrane curvature (Beck et al., 2008; Krauss et al., 2008; Lee et al., 2005) and can reduce membrane rigidity, thus facilitating membrane deformation (Settles et al., 2010). The only possible GTPase or Arf candidate in endocytic CCPs/CCVs is Arf6, but this is not found in significant amounts (Borner et al., 2012) and depletion of Arf6 only marginally affects endocytosis (Montagnac et al., 2011). Hence, we propose that in endocytic CCPs, the AH0 of the abundant adaptor CALM assumes the role of a curvature sensor/driver similar to the AHs of small GTPases in other transport vesicles.

If one considers that nonsense mutations in CALM cause severe anemia and reduced life-span in mice (Klebig et al., 2003; Scotland et al., 2012), that complete deletion of the gene additionally causes other severe phenotypes resulting in >90% pre-weaning mortality (Ishikawa et al., 2014; Suzuki et al., 2012), that CALM is implicated in alterations in cognitive function with increasing age (Mengel-From et al., 2011), and finally that mutations in CALM are a risk factor for Alzheimer disease (Harold et al., 2009), the apparent inability of CALM depletion to affect endocytic rate in cultured cells previously reported in several studies was difficult to understand. However, by measuring the more physiological steady-state endocytic uptake rate at 37°C rather than using a 4°C block (Miller et al., 2011; Motley et al., 2003, 2006), we show that in fact CALM depletion does cause a marked reduction in endocytic rate of both EGF and transferrin. This is in at least partial agreement with a very recent study showing that a conditional CALM knockout specifically caused reduced transferrin uptake in mouse erythroblasts, although the authors suggest that CALM may be involved in a specialized endocytic mechanism in these cells (Ishikawa et al., 2014).

In conclusion, we have demonstrated that the N-terminal 18 residues of CALM form a previously unsuspected amphipathic helix (AH0) and that this structural element plays a central role in the ability of CALM to strongly influence CCP/CCV size, formation, rate of maturation, and thus endocytic uptake. We propose that it is the disruption of these properties in combination with effects on CALM’s ability to drive the internalization of small R-SNAREs (Koo et al., 2011; Miller et al., 2011) that explains the pathophysiological effects caused by mutations in the CALM gene.

## Experimental Procedures

For additional information, including protein chemistry, reagent preparation, and constructs used, see the Supplemental Experimental Procedures.

### Cell Culture

HeLaM-VAMP8-HA cells were selected under 0.5 mg/ml G418 and additionally transduced with myc-tagged, siRNA-resistant WT or mutant versions of CALM for the add-back experiments, using the retrovirus Phoenix system. The derived cells were selected with 0.2 mg/ml hygromycin B and 0.5 mg/ml G418. The highest level of myc-CALM expression was similar to the levels of endogenous CALM. Endogenous CALM was depleted with siRNA ACAGTTGGCAGACAGTTTA at 20 nM for 72 hr (Miller et al., 2011). Endogenous AP2-μ2 was depleted with siRNA AGTGGATGCCTTTCGGGGTCA (Motley et al., 2003) at 20 nM for 96 hr.

### Immunofluorescence and Light Microscopy Imaging

Indirect immunofluorescence was performed after paraformaldehyde fixation and permeabilization with 0.1% Triton X-100 or 0.5% saponin. For imaging of transferrin endocytosis, the living cells were first serum-starved for 30 min, followed by exchange for medium supplemented with Alexa Fluor-conjugated transferrin (Invitrogen) for the indicated times. For TIRF microscopy, HEK293 cells were cultured and transfected using CALM-GFP and AP2 μ2-mCherry as detailed previously (Gage et al., 2005; Taylor et al., 2011). Cells were plated on clean coverslips on the same day as transfection and imaged 48 hr later to allow several cell divisions and to give transfected cells time to adapt moderate protein overexpression (Taylor et al., 2011, 2012). To determine CCP maturation time in CALM knockdown cells, HT1080 cells were transfected with either CALM or control siRNA followed by transfection with CALM-GFP and AP2 μ2-mCherry. The maturation time of CCPs was determined as described previously. For STED super resolution microscopy, samples were fixed and processed as described above. For microscopy, a Leica TCS SP8 gSTED equipped with a 100×/1.4 Oil STED Orange lens and a 592 nm depletion laser was used. Further details on STED imaging and quantification are summarized in the Supplemental Experimental Procedures.

### Surface Plasmon Resonance-Based Analysis of Membrane Binding

Binding of recombinant CALM ANTH to membranes was determined with a BIAcore 3000 SPR biosensor (GE Healthcare). Details of the method are described elsewhere (Honing et al., 2005) and in the Supplemental Experimental Procedures.

### Single Liposome Curvature Assay

Liposomes for single liposome curvature assay were prepared using a previously described lipid hydration method (Bhatia et al., 2009; Hatzakis et al., 2009). The size of liposomes was calibrated by combining confocal fluorescence microscopy and dynamic light-scattering measurement (Kunding et al., 2008). Single isolated liposomes of different diameters labeled by DOPE-Atto 655 were immobilized on a Neutravidin functionalized microscope glass surface. A total of 500 nM CALM ANTH-WT and CALM ANTH(ΔH0) labeled with Alexa 488 were allowed to bind the surface attached liposomes from solution. The fluorescently labeled protein and lipid were sequentially imaged, and the integrated intensities extracted and used to quantify the density of bound protein and the size of the individual liposomes.

All experiments were performed in a 10 mM Tris (pH 7.4), 170 mM NaCl, 0.05 mM TCEP buffer. For all samples, liposomes were imaged before the addition of protein to ensure that the binding molecule did not affect liposome morphology or fluorescence. Images were analyzed as described elsewhere (Hatzakis et al., 2009). We have previously shown that recruitment by membrane curvature follows a power law and quantified the recruitment potency as the slope of a straight-line fit to a log-log representation of protein density versus liposome diameter. In this representation of the data, a more negative slope represents a stronger recruitment by membrane curvature. Technical details are explained in full in the Supplemental Experimental Procedures.

### Liposome Preparation and In Vitro Liposome Tubulation Assay

Brain polar lipid extract (BPLE) and PtdIns4,5P_2_ (Avanti Polar Lipids) were hydrated in chloroform:methanol (4:1) supplemented with HCl; 0.5% PtdIns4,5P_2_ BPLE was dried down with argon and rehydrated in 20 mM Tris (pH 7.4), 170 mM NaCl. The solution was extruded through a 200 nm polycarbonate membrane (Nuclepore) 21 times; 10 μl of 1 mg/ml 200 nm liposomes was used in a 100 μl reaction. Control samples contained no protein. CALM proteins (0.5 μM) were incubated for 1 min at 20°C. A formvar carbon-coated/glow-discharged EM grid was immediately placed on the surface of a 50:50 mix of the liposome protein solution and 9:1 2% methyl cellulose:2% uranyl acetate (to prevent air-drying artifacts of delicate structures as routinely used in the preparation of ultrathin frozen sections for immunolabelling; Slot and Geuze, 2007), incubated for 1 min, removed, wicked, and dried overnight. The grids were viewed (TEM, Phillips) and images were captured (Megaview III; Olympus). A new batch of liposomes and protein was used for six repeated experiments, resulting in final counts of several hundred liposomes for each condition. Liposome tubulation was categorized, counted, and plotted (Excel, Microsoft).

### Transmission Electron Microscopy

Cells were fixed with 2.5% glutaraldehyde/2% paraformaldehyde, post-fixed with 1% osmium tetroxide, dehydrated, and embedded in epoxy resin. Randomly cut and orientated sections were stained (Reynolds, 1963) and CCPs were recorded with a charge-coupled device (CCD) camera (Megaview III; Olympus) on a CM100 transmission electron microscope (TEM; Philips). One hundred profiles were traced with a Wacom Bamboo tablet and overlaid using Adobe Illustrator CS6.

### Immunogold Electron Microscopy

Cells were fixed with 0.1% glutaraldehyde / 4% paraformaldehyde, infused with 1.7 M sucrose/15% polyvinyl pyrolidone, and frozen in liquid nitrogen. Sections were cut in a cryo-ultramicrotome (Ultracut UCT/EM FCS; Leica), collected with 1% methyl cellulose/1.15 M sucrose, and immunolabelled with mouse anti-myc (Upstate) followed by rabbit anti-mouse (DAKO) and protein A: 15 nm colloidal gold (Utrecht University, The Netherlands). Images were captured using a CCD camera (Eagle 4K) on a Tecnai G2 Spirit BioTWIN TEM (FEI).

### Internalization of [^125^I] Transferrin and [^125^I] Epidermal Growth Factor

The internalization of iodinated transferrin (Tf) and EGF was determined according to (Jiang et al., 2003) and (Huang et al., 2004). In brief, triplicates of cells were grown on 12-well dishes and incubated in either 2 μg/ml ^125^I-labeled Tf or 1 ng/ml ^125^I-labeled EGF at 37°C for 2, 4, and 6 minutes. Subsequently, the cells were placed on ice and washed. Thereafter, the cells were incubated twice in 0.2 M acetic acid (pH 2.6), 0.5 M NaCl for 5 min to remove the surface-bound ligand. Finally, the cells were solubilized and collected in 1 M NaOH. The acid-releasable radioactivity represented surface-bound ligands, whereas the acid-resistant fraction solubilized in NaOH represented the internalized fraction. The latter was plotted against time. The resulting linear regression coefficients represent the endocytic rate constants k_e_.

## Supplemental Information

Document S1. Supplemental Experimental Procedures, Figures S1–S5, and Table S1

Document S2. Article plus Supplemental Information
