# Supplementary material for: CALM Regulates Clathrin-Coated Vesicle Size and Maturation by Directly Sensing and Driving Membrane Curvature
Source: Dev Cell. 2015 Apr 20;33(2):163–75. doi: 10.1016/j.devcel.2015.03.002 (PMC4406947; doi:10.1016/j.devcel.2015.03.002)
Supplement: Document S1. Supplemental Experimental Procedures, Figures S1–S5, and Table S1 [file mmc1.pdf]

Developmental Cell

Supplemental Information

CALM Regulates Clathrin-Coated Vesicle Size and Maturation by Directly Sensing and Driving Membrane Curvature

Sharon E. Miller, Signe Mathiasen, Nicholas A. Bright, Fabienne Pierre, Bernard T. Kelly, Nikolay Kladt, Astrid Schauss, Christien J. Merrifield, Dimitrios Stamou, Stefan Höning, and David J. Owen

Supplementary Figure 1 linked to Figure 1

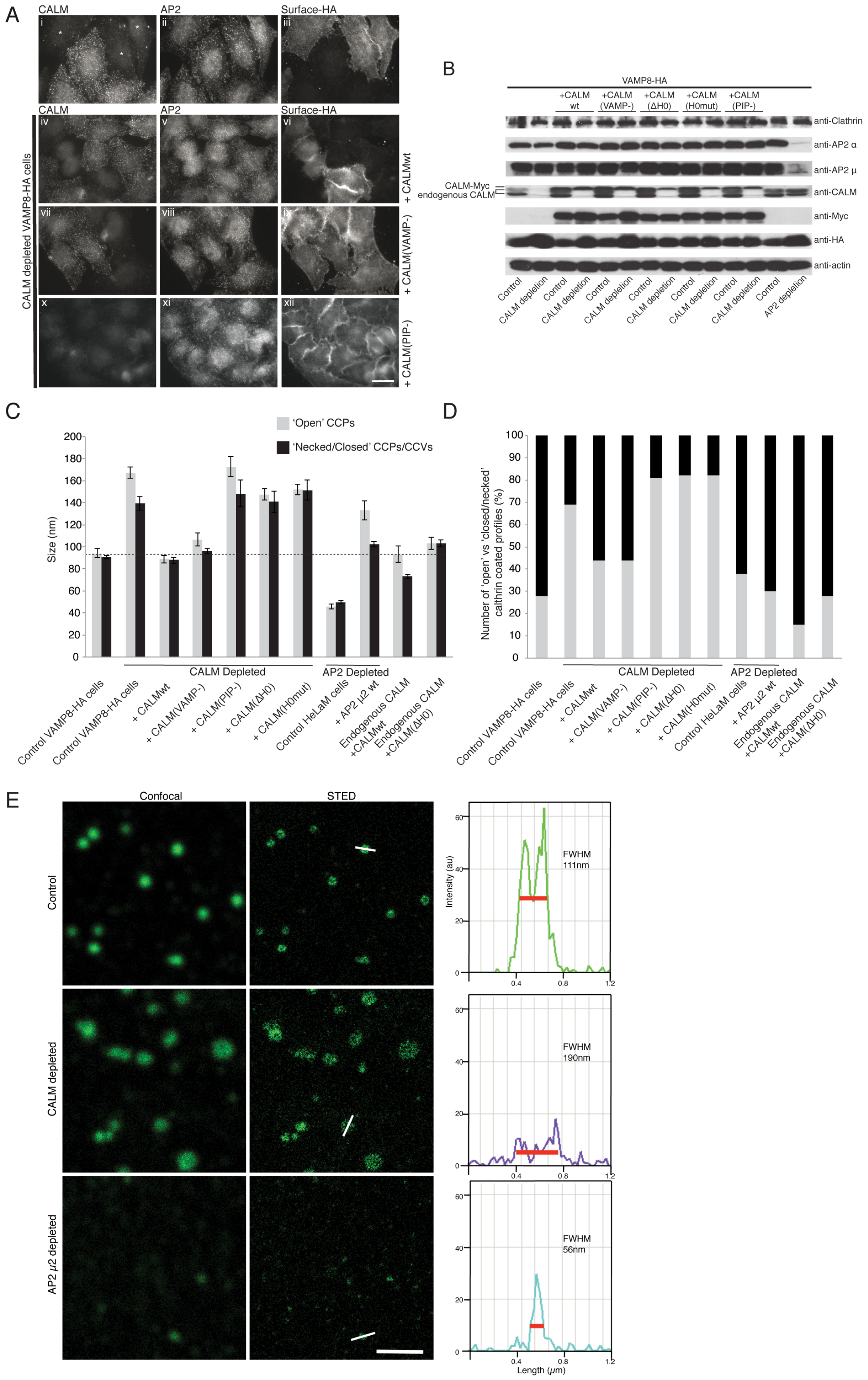

(A) All cells express VAMP8-HA and were labelled for CALM, AP2 and anti-HA (VAMP8-HA). (i-iii) Cells depleted of endogenous CALM (marked with *) were mixed with non CALM-depleted control cells. All other images (iv- xii) show cells depleted of endogenous CALM, and those with CALM positive punctae express full-length human siRNA-resistant CALM variants. CALM depletion or CALM add back variants do not apparently affect the localisation of AP2 (ii,v,viii,xi). In control cells VAMP8-HA is internalised by CALM but remains trapped on the plasma membrane when CALM is depleted (iii). In CALMwt rescue cells (iv-vi) CALM co-localises with AP2 punctae and VAMP8-HA is internalized. (vii-ix) CALM (VAMP-) add back cells show CALM and AP2 colocalisation, but this SNARE binding mutant does not endocytose VAMP8. (x-xii) CALM(PIP-) add back cells do not endocytose VAMP8 due to the inability of the construct to bind PtdIns4,5P_2 and therefore localise to the plasma membrane. Scale bar represents 20μm. (B) Western blots of cell lines showing the specificity of the CALM and AP2 μ2-subunit depletion. Extracts of cells expressing VAMP8-HA alone or expressing VAMP8-HA and myc-tagged siRNA-resistant CALMwt, CALM(VAMP-), CALM(ΔH0), CALM(H0mut) or CALM(PIP-) were probed with anti-clathrin, anti-AP2 α-subunit, anti-AP2 μ2-subunit, anti-CALM, anti-Myc, anti-HA or anti-actin (loading control). The blots show that each myc-tagged CALM construct is expressed at a similar level to one another and to endogenous CALM. In addition the CALM depletion specifically depletes the endogenous CALM but not the si-RNA resistant CALM. The specificity of the AP2 knockdown is also shown. Depletion of either of the major components in CCVs does not affect the level of the other. (C) Bar graph showing the average size of 'open' CCPs and 'necked'/'closed' CCPs/CCVs for control cells, CALM depleted cells

without or with re-expression of various CALM constructs. AP2 depletion and rescue is also represented as well as the overexpression of CALMwt and CALM(ΔH0). The width of 'open' profiles is measured at an average half depth of the CCP and shown in grey. The diameter of CCVs is averaged from two perpendicular measurements of the vesicle and is shown in black. The dotted line shows the average control size of CCPs/CCVs. Cells deleted of CALM without or with expression of CALM(PIP-), CALM(ΔH0) and CALM(H0mut) show a large increase in CCP/CCV size (~1.6x). Cells overexpressing CALMwt show a decrease in CCV size (~1.3x) not observed for overexpression of CALM(ΔH0) . In addition clathrin coated profiles of cells depleted of AP2 are ~2x smaller than those found in control cells. Error bars represent the standard error of >100 measurements). (D) A bar graph showing the number of 'open' CCPs (grey) versus 'necked'/'closed' CCPs/CCVs (black) for each cell line. In control cells the ratio of 'open' to 'necked'/'closed' profiles is 30:70, this ratio is conserved in cells depleted of CALM and expressing CALMwt or CALM(VAMP-). The ratio is reversed to 70:30 in CALM depleted cells and in those cell lines expressing CALM(PIP-), CALM(ΔH0) and CALM(H0mut) after CALM depletion. In those cells overexpressing CALMwt, there is an increase in the number of 'necked'/'closed' structures compared to 'open' (85:15) indicating an increase in CALM expression increases the number of CCVs, and therefore contributes to increased curvature and subsequent closure/budding of CCVs. (E) Clathrin-stained CCPs/CCVs were imaged by confocal and STED microscopy. The resulting STED image was then used to measure the diameter of the coated structures using the full-width at half maximum method. An example of one such measurement is shown for control, CALM depleted and AP2 μ2 depleted cells (graphs). Scale bar represents 0.5 μm

Supplementary Figure 2 linked to Figure 2

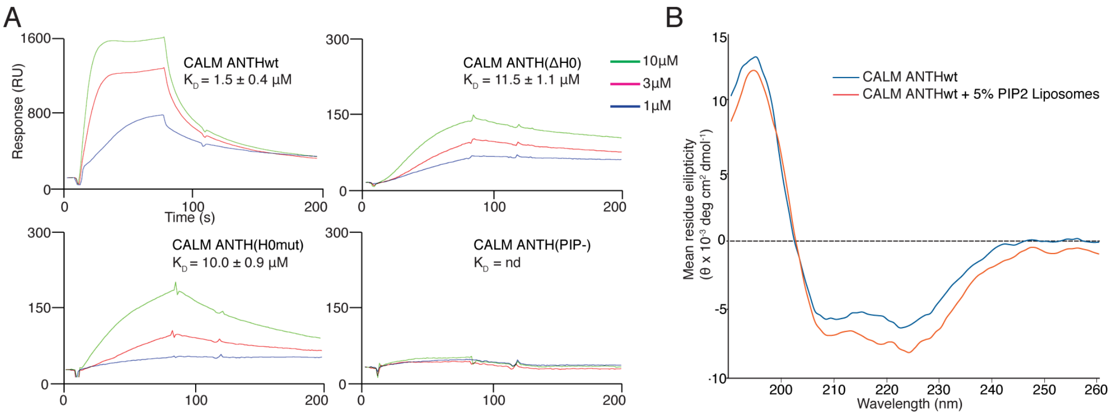

(A) Representative sensorgrams of liposome-based SPR traces for wt and mutant forms of CALM binding to PtdIns4,5P2-containing liposomes at protein concentrations of 10μM 3μM and 1μM. (B) CD traces of CALM ANTHwt domain at the same concentration in the presence (red line) and absence (blue line) of PtdIns4,5P2-containing liposomes. In the presence of liposomes there is a clear increase in α-helical content, indicated by the markedly enhanced features at both 222nm and 208nm.

Supplementary Figure 3 linked to Figure 3

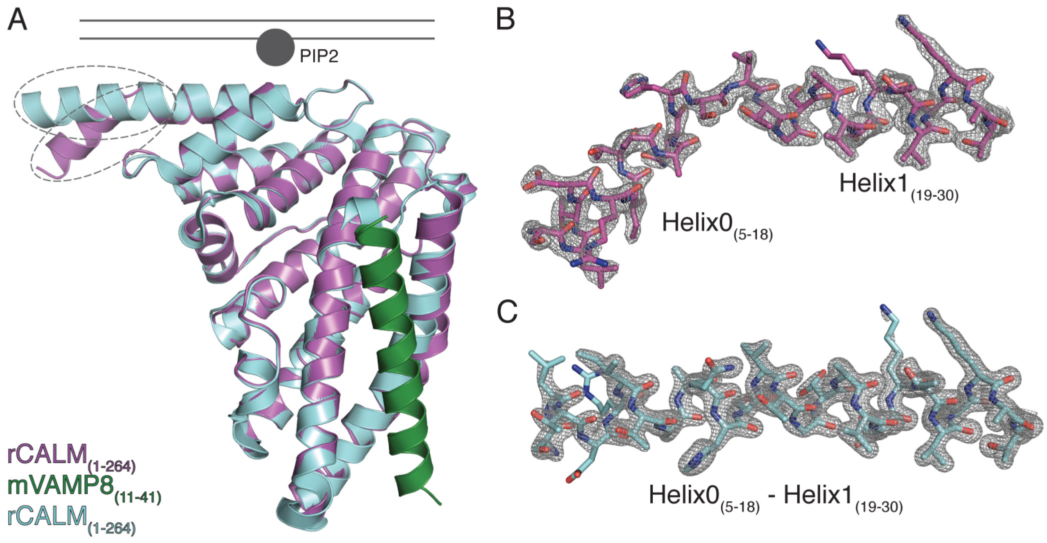

(A) Overlay of the ribbon representation of the CALM ANTH$_{(1\text{-}264)}$ (blue) and CALM ANTH:VAMP (purple/green) structures. (B) The electron density of the N-terminus of CALM in the CALM ANTH:VAMP structure at 2Å resolution and (C) the same region in the CALM ANTH$_{(1\text{-}264)}$ structure at 1.7Å resolution.

Supplementary Figure 4 linked to Figure 4

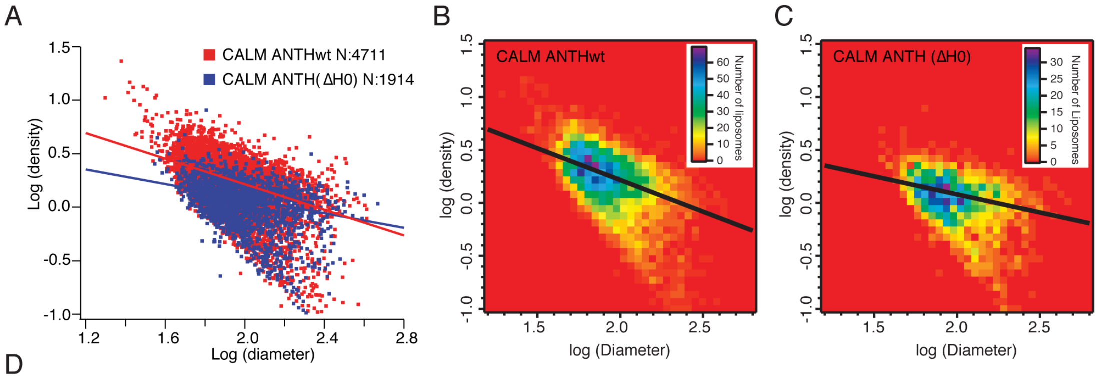

D

	Linear fitting slope ± SD
CALM ANTHwt	-0.64 ± 0.04
CALM ANTH(ΔH0)	-0.34 ± 0.04

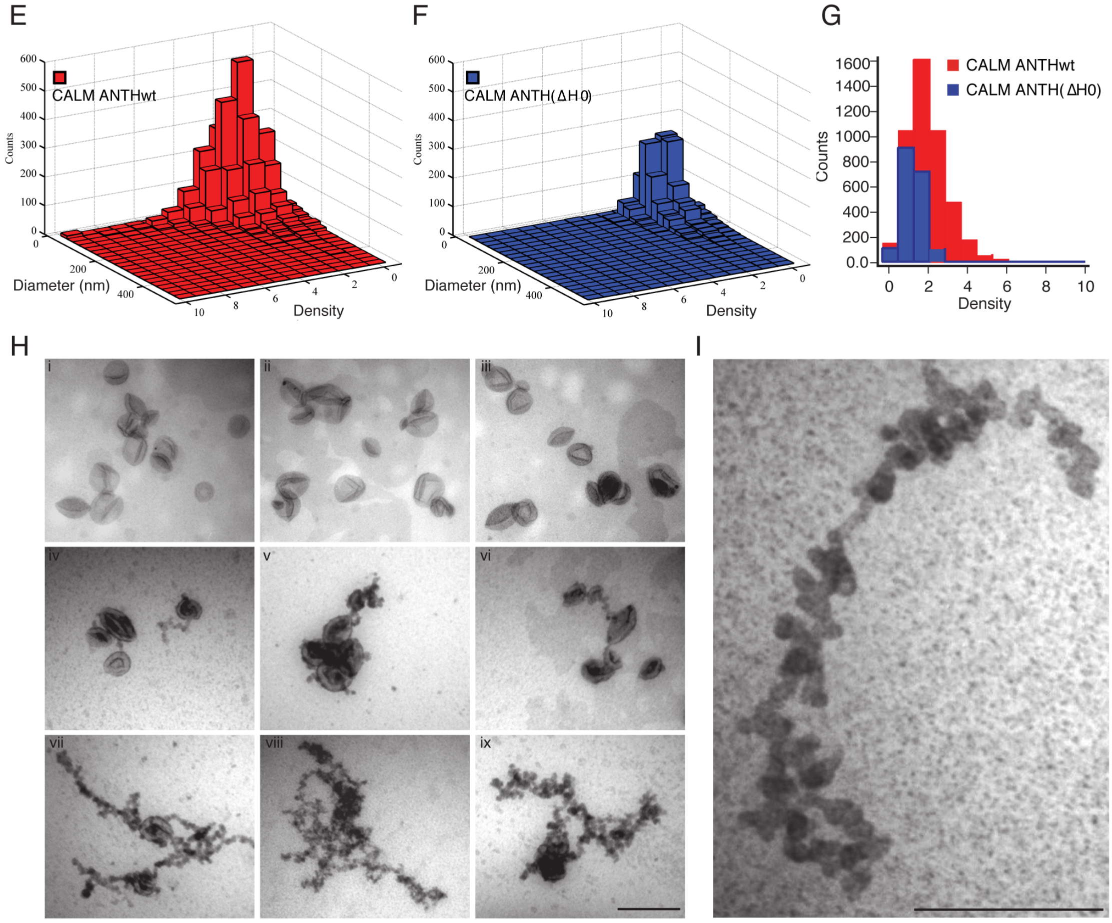

(A) Log-log representation of the data presented in Figure 4A. Because membrane recruitment follows a power law, the recruitment potency can be described by the slope of a straight-line fit to log-log representation of protein density versus liposome diameter. Red line, straight line fit to CALM ANTHwt

dataset. Blue line, straight-line fit to CALM ANTH(ΔH0) dataset. (B-C) CALM ANTHwt and CALM ANTH(ΔH0) heat map of the log-log representation in A. Colour scale, number of single liposomes. Black lines, CALM ANTHwt and CALM ANTH(ΔH0) straight-line fits from A. Both fits clearly follow the bins with the highest number of liposomes, demonstrating that the most frequently measured densities dictate the linear fits. (D) Average slope of straight-line fit (as in A) and standard deviation obtained from three independent measurements of CALM ANTHwt and CALM ANTH(ΔH0) to PtdIns4,5P_2 containing liposomes. (E-F) Bivariate histograms of CALM ANTHwt and CALM ANTH(**Δ**H0) density versus liposome diameter. The histograms reveal the sampling frequency and distribution of single liposome protein densities for CALM ANTHwt and CALM ANTH(**Δ**H0). The two protein samples display distinct density distributions, with CALM ANTHwt populating markedly higher densities that CALM ANTH(**Δ**H0). (G) Marginal histograms of the collapsed density histograms data in E and F. A comparison of the two histograms reveal that CALM ANTHwt and CALM ANTH(ΔH0) populate markedly different densities. (H) Typical examples of *in vitro* liposome tubulation with CALM ANTHwt. Control liposomes (i-iii) do not show any tubulation. (iv-vi) Liposomes that have been categorised as exhibiting slight tubulation are sometimes seen when incubated with 0.5μM CALM ANTHwt for one minute. More often liposome tubulation seen when incubated with 0.5μM CALM ANTHwt for one minute is 'pearlised' (vii-ix) and classified as extensive. Scale bar represents 500nm. (I) Zoom of the typical 'pearlised' tubulation by CALM ANTHwt. Scale bar represents 500nm.

Supplementary Figure 5 linked to Figure 5

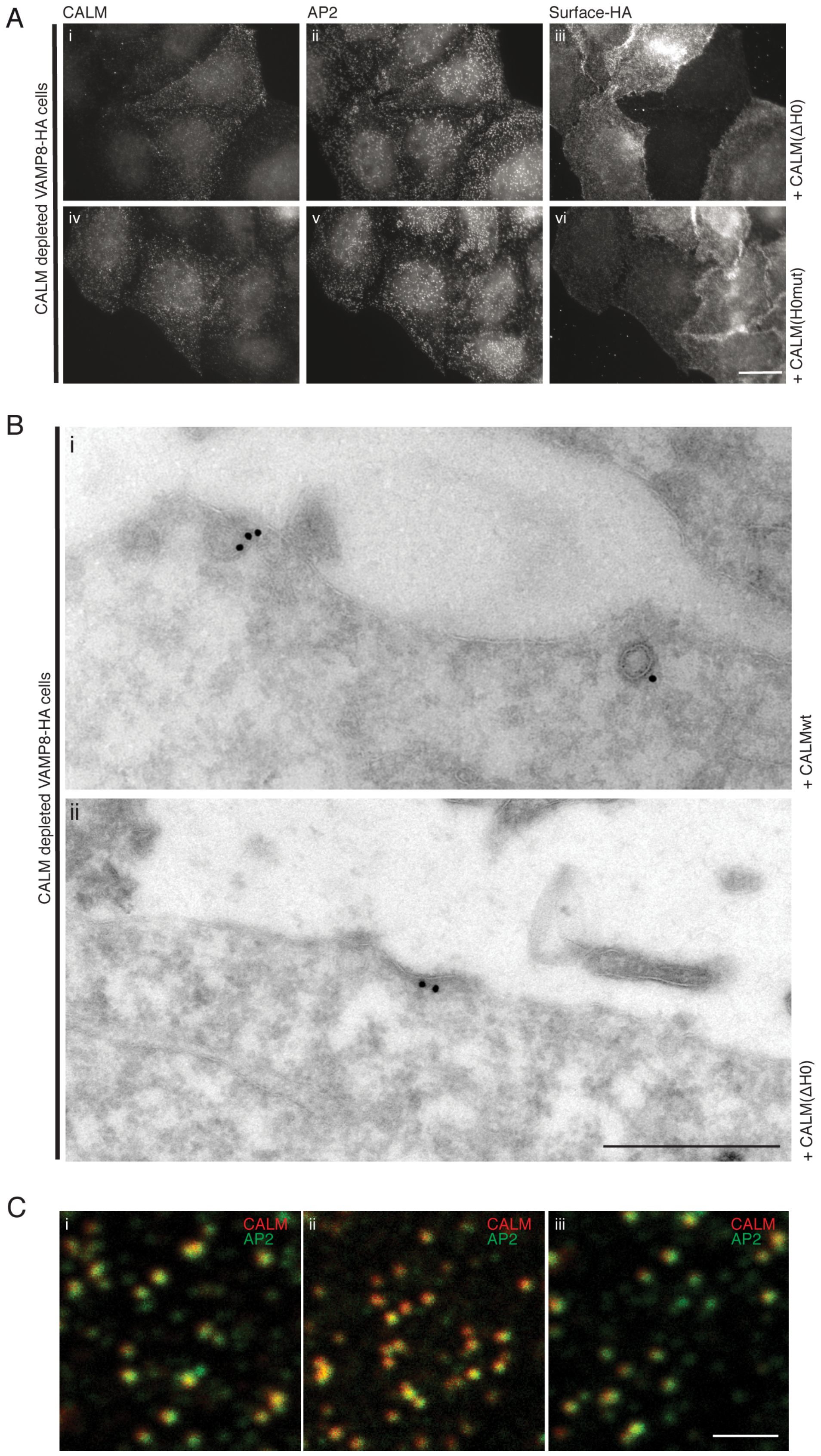

(A) All cells express an HA-tagged version of the endocytic SNARE VAMP8. For immunofluorescence microscopy cells were labelled for CALM, AP2 and anti-HA (VAMP8-HA). All cells were depleted of endogenous CALM, those cells with CALM punctae stably express full length human siRNA-resistant CALM(ΔH0) (i-iii) or CALM(H0mut) (iv-vi). CALM depletion without or with re-expression does not affect the localisation of AP2 (ii, v). When CALM is absent VAMP8-HA is trapped on the plasma membrane (iii, vi). CALMwt, CALM(ΔH0) and CALM(H0mut) co-localise with AP2 positive punctae (ii, v) and support internalisation of VAMP8-HA (iii, vi). Scale bar represents 20μm.

(B) Immunogold labelling of (i) myc-tagged CALMwt and (ii) CALM(ΔH0) reveal that the epitope-tagged constructs are correctly targeted to clathrin-coated structures in CALM depleted cells. Scale bar represents 200nm. (C) The co-localisation of AP2 (green) and CALM (red) punctae is shown in (i) control cells (ii) CALM depleted cells expressing CALM ANTHwt and (iii) CALM depleted cells expressing CALM(ΔH0). Scale bar represents 1μm.

Supplementary Table1 – linked to Figure 3.

Data collection and refinement statistics

Numbers in parentheses refer to the appropriate outer shell

	**CALM$_{\mathbf{ANTH(1\text{-}264)}}$**	**CALM$_{\mathbf{ANTH(1\text{-}264)}}$:VAMP8$_{\mathbf{(11\text{-}41)}}$**
**Data collection**		
Beamline	Diamond I03	Diamond I03
Detector	ADSC Q315 CCD	ADSC Q315 CCD
Wavelength (Å)	0.9763	0.9763
Resolution limits (Å)	60.5–1.7 (1.74–1.70)	91.1–2.0 (2.08–2.03)
Space group	*C*2	*C*2
Unit cell dimensions (Å, °)	*a* = 95.9<br>*b* = 121.1<br>*c* = 62.4<br>ß = 110.7	*a* = 161.1<br>*b* = 100.3<br>*c* = 104.0<br>ß = 118.9
Unique reflections	72,568 (5,397)	93,133 (6,910)
Redundancy	4.3 (4.3)	3.7 (3.7)
Completeness (%)	99.4 (99.9)	99.7 (99.8)
<*I*/s*I*)>	9.8 (2.1)	10.0 (1.9)
R_{merge}[a]	0.071 (0.484)	0.079 (0.731)
R_{meas}[b]	0.081 (0.553)	0.093 (0.855)
R_{pim}[c]	0.038 (0.264)	0.048 (0.440)
**Refinement**		
Resolution limits (Å)	35.5–1.7 (1.72–1.70)	45.6–2.0 (2.05–2.03)
Reflections in working set	72,462 (2,670)	93,106 (2,933)
Reflections in test set	3,652 (138)	4,667 (162)
R_{work} (%)[d]	0.170 (0.288)	0.182 (0.294)
R_{free} (%)[e]	0.191 (0.300)	0.199 (0.365)
Number of molecules/complexes per asymmetric unit	2	3
Number of atoms (protein/water/other[f])	4,225/444/0	6,844/473/8
Residues in Ramachandran favored region (%)	98.3	99.2
Ramachandran outliers (%)	0.0	0.0
r.m.s.d bond lengths (Å)[g]	0.010	0.008
r.m.s.d bond angles (°)[g]	1.110	0.911
Average *B* factors (Å^2) (protein/solvent)	31.1/40.68	41.2/46.8

[a]$R_{merge} = \Sigma_{hkl} \Sigma_i |I(hkl;i) - \langle I(hkl) \rangle| / \Sigma_{hkl} \Sigma_i I(hkl;i)$, where *I(hkl;i)* is the intensity of an individual measurement of a reflection and <*I(hkl)*> is the average intensity of that reflection

[b]R_{meas} is the redundancy-independent merging *R* factor (Diederichs and Karplus, 1997)

[c]R_{pim} is the precision-indicating merging *R* factor (Weiss et al., 1998)

[d]$R_{work} = \Sigma_{hkl} ||F_{obs}| - |F_{calc}|| / \Sigma_{hkl} |F_{obs}|$, where $|F_{obs}|$ and $|F_{calc}|$ are the observed and calculated structure factor amplitudes, respectively

[e]R_{free} equals *R* of the cross-validation (test) set, 5% of the data removed prior to refinement

[f]Other indicates PO_4^{3-} ions

[g]r.m.s.d. is root mean square deviation from ideal geometry

Supplementary Materials and Methods

Constructs used in this study

pBMN-CALM (human) and mutants thereof, pBMN-CALM Met244Lys, pBMN-CALM ΔH0 $_{(19-652)}$, pBMN-CALM$_{(1-652)}$ Leu6Ser Ile10Ser Val17Ser and pBMN-CALM$_{(1-652)}$ Lys28Glu Lys38Glu Lys40Glu. pGEX-4T2 CALM ANTH$_{(1-289)}$ (rat) and mutants thereof, pGEX-4T2 CALM ANTH$_{(20-289)}$ (ΔH0), pGEX-4T2 CALM ANTH$_{(1-289)}$ Met244Lys (VAMP-), pGEX-4T2 CALM ANTH$_{(1-289)}$ Leu6Ser Ile10Ser Val17Ser (H0mut), pGEX-4T2 CALM ANTH$_{(1-289)}$ Lys28Glu Lys38Glu Lys40Glu (PIP-), pGEX-4T2 CALM ANTH Cys27Ser + C-Term CysGlyCys tag and pGEX-4T2 CALM ANTH(ΔH0) Cys27Ser + C-Term CysGlyCys tag. pCALM-GFP and pμ2-mCherry (Taylor et al., 2011)

Antibodies and ligands used in this study

Polyclonal rabbit anti-clathrin (gift of Margaret Robinson), polyclonal goat anti-CALM (Santa Cruz), polyclonal rabbit anti-AP2 β2 (gift of Margaret Robinson), monoclonal mouse anti-AP2 α subunit (Abcam), monoclonal mouse anti-AP2 μ2-subunit (BD Transduction Laboratories), monoclonal mouse anti-Myc (Millipore), monoclonal mouse anti-HA (Covance) and monoclonal mouse anti-actin (Abcam). Secondary antibodies appropriate to primary antibodies as well as EGF-Alexa488 and Tf-Alexa565 as IF probes for endocytosis via their respective receptors were all from Invitrogen.

Western blotting

Levels of protein expression and depletion efficiency were analysed by western blots probed with appropriate antibodies.

Protein expression and purification

All recombinant proteins were expressed in BL21(DE3) pLysS cells for 16hr at 22°C after induction with 0.2mM IPTG at 37°C. Following lysis, all GST-CALM fusion proteins were purified on Glutathione Sepharose 4B in 20mM Tris pH 7.4, 170mM NaCl, 4mM DTT. The N-terminal GST tag was cleaved with thrombin overnight at 20°C. The cleaved proteins were then eluted and the thrombin cleavage was halted by the addition of AEBSF. All proteins were then further purified by S200 gel filtration in 20mM Tris pH 7.4, 170mM NaCl, 0.5mM TCEP.

Surface plasmon resonance (SPR) based analysis of membrane binding

Liposomes for SPR studies were composed of synthetic 1-palmitoyl-2-oleoyl-sn-glycero-3-phosphocholine (POPC), 1-palmitoyl-2-oleoyl-sn-glycero-3-phosphoethanolamine (POPE) and $PtdIns4,5P_2$ at a ratio of 85%:20%:5%. Liposomes (300µM final concentration) were captured on the four flow cells of a L1 surface in 5min injections at a flow rate of 10µl/min using 20mM Tris pH 7.4, 170mM NaCl, 0.5mM TCEP as buffer. Subsequently the flow rate was set to 30µl/min and the surfaces were stabilized with two injections of 50mM NaOH for 10sec. Proteins were then injected for 60sec followed by a dissociation period of 120sec. Protein that remained bound to liposomes was removed by a 5sec pulse injection of NaOH. Such an experimental cycle was repeated at least five times with different concentrations ranging from 0.5µM–20µM. The resulting sensorgrams were used to calculate the K_D values after background

subtraction (binding to PC/PE liposomes) using the manufacturers evaluation software (version 4.1.1) assuming a 1:1 interaction.

Calculating % coverage of pearlised PtdIns4,5P_2 containing liposomes by CALM

In our liposome tubulation system we can estimate the % membrane coverage of the CALM ANTH that results in the production of endocytic CCV sized 'pearlised' tubes. We estimate the % coverage of CALM to be ~16%, close to the value of ~30% calculated for the surface occupation by epsin proposed to generate narrower flat-sided tubes from synthetic liposomes (Kozlov et al., 2014)

For this calculation we have had to make some assumptions similar to those used in (Kozlov et al., 2014) such as that projecting the structure down onto the membrane will give a representative protein footprint. Our value may be an underestimation of the amount of CALM ANTH surrounding the 'pearls' as the affinity of CALM for the membrane will increase as curvature ie pearlising increases since CALM shows increased/preferred binding to membranes of <100nm curvature (see data in Figure 4). Further PtdIns4,5P_2 local concentrations may be higher than the 2.5μM overall estimate as it has been shown that PtdIns4,5P_2 tends to self aggregate into patches (Stachowiak et al., 2012)

CALM concentration 0.5μM and PtdIns4,5P_2 concentration estimated at 2.5μM (from PtdIns4,5P_2 already present in brain lipid extract from Avanti and

supplemented).

and

K_D for 200nm liposomes 1.5μM at 170mM salt used in tubulation experiments derived by SPR

Solving resulting quadratic equation, concentration of CALM on membrane 0.3μM (probably an underestimate see above)

Assuming an overall lipid concentration of 100uM based on the amount of lipids used and an average MW of 1000Da, there are ~6 x 10^{19} lipid headgroups present in a reaction.

From the membrane model (Heller et al., 1993) there are ~900 lipid headgroups packed in a square 262Å x 262Å so the total amount of lipid gives a total lipid surface area of 2.3 x 10^{21} $Å^2$

Each CALM molecule has a footprint of ~2000$Å^2$ and as the concentration of bound CALM is 0.3μM there will be 1.8 x 10^{17} molecules of CALM bound which will take up ~3.6 x 10^{20} $Å^2$.

So fraction of membrane surface covered by CALM is 3.6x10^{20} / 2.3x10^{21} = ~16%

Calculation of CALM coverage on endocytic CCVs

CALM membrane footprint derived from structure ~2000Å2

AP2 membrane footprint derived from structure ~8500Å2

Surrounding a 50nm (500Å) diameter vesicle (Surface area 775000Å2) there are 60 clathrin triskelia and by quantitative proteomic data (Borner et al., 2012) around 60AP2s (SA ~510000Å2) and 60-70 CALMs (SA~130000Å2).

Hence around 17% of an endocytic vesicle membrane is covered by CALM.

We now refer briefly to this value in the discussion section of the paper, although as with any modeling calculations there will undoubtedly be assumptions made that are not entirely valid, especially in vivo where membrane composition is complicated and other factors are at work. Also it should be noted that this value will be much higher than the value for surface coverage of endocytic CCVs by epsin *in vivo* as quantitative proteomics (Borner et al., 2012) indicates that there is little epsin (<1% of the adaptor complement) in endocytic CCVs.

Circular dichroism

Two identical 0.5ml samples of 1mg/ml CALM in 50mM sodium phosphate pH7.4 were added to either 500µl 50mM sodium phosphate pH7.4 or 500µl BPLE 5% PtdIns4,5P_2 liposomes that had been resuspended in 50mM sodium phosphate pH7.4. CD spectra were collected in a Jasco J-810 and 20 runs averaged using manufacturer's software. Buffer only or buffer+liposome background traces were subtracted as appropriate and the resulting spectra

overlayed in Savitsky-Golay convolution width 15. All experiments were repeated at least 4 times.

Single liposome curvature (SLiC) assay

Materials

1,2-Dioleoyl-sn-Glycero-3-Phosphocholine (DOPC); 1,2-Dioleoyl-sn-Glycero-3-Phosphatidylethanolamine-N-(cap biotinyl) (DOPE-Biotin); 1,2-Dioleoyl-sn-Glycero-3-Phosphoethanolamine (DOPE); 1,2-dioleoyl-*sn*-glycero-3-phospho-(1'-myo-inositol-4',5'-bisphosphate) (DOPE-PI(4,5)P_2) were all acquired from Avanti Polar Lipids Inc (Alabaster, AL). 1,2-Dioleoyl-sn-Glycero-3-Phosphatidylethanolamine-Atto655 (DOPE-Atto655) was from Atto-Tec. Liposomes for SLiC were prepared using a previously described lipid hydration method (Bhatia et al., 2009; Hatzakis et al., 2009). In brief, lipids dissolved in chloroform were mixed in a glass vial, at the molar ratios DOPC;DOPE;DOPE-biotin;DOPE-Atto655;DOPE-PI(4,5)P_2 (69.3;20;0.2;0.5;10). The solution was dried under nitrogen flow and incubated in vacuum for 4hr. Liposomes were rehydrated by adding a 290mM D-Sorbitol solution to the lipid film, for a final lipid concentration of 0.5g/l. The mixture was incubated overnight at room temperature. The liposomes were then subjected to 20 freeze-thaw cycles to minimize multilamellarity and extruded once through a single polycarbonate filter with a pore size of 400nm in pressurized extruder (Avanti Polar Lipids Inc). The liposomes where flash-frozen in liquid nitrogen and stored at -21°C.

Labelling of CALM ANTH proteins

Alexa Fluor 488 C_5 maleimide (Life Technologies) was dissolved at 50mg/ml in DMSO and added dropwise to give a twofold molar excess over 2.8mg/ml of CALM ANTHwt or CALM ANTH(ΔH0) in 20mM Tris pH 7.4, 170mM NaCl, 0.5mM TCEP. The reaction was incubated for 2hr at 20°C with continuous gentle inversion (~10rpm) in a foil-wrapped tube, quenched with the addition of 4mM DTT, dialyzed against 20mM Tris pH 7.4, 170mM NaCl, 4mM DTT to remove the bulk of the unincorporated dye, and, finally, purified by size exclusion chromatography on an S200 column (equilibrated in 20mM Tris pH 7.4, 170mM NaCl, 4mM DTT) in the dark.

Functionalization of microscope glass surfaces

Microscope chamber parts and glass slides were cleaned according to previously published procedures (Christensen et al., 2012). Glass slides were dried in nitrogen, plasma etched for 120sec., mounted in a microscope chamber and incubated with a mixture of 1000:6 PLL-g-PEG:PLL-g-PEG-Biotin (SuSoS AG, Switzerland) (1g/l) in surface buffer (15mM HEPES pH 5.6) for 30min. After consecutive washing steps x10 with buffer (10mM Tris pH 7.4, 170mM NaCl, 0.05mM TCEP) the passivated surfaces were incubated with 0.1g/l Neutravidin in surface buffer for 10min, followed by additional washing x10.

Liposome size calibration

We previously described a methodology for calibrating liposome diameter by combining confocal fluorescence microscopy and dynamic light scattering (DLS) measurements (Kunding et al., 2008). In brief, the integrated intensity of a liposome labelled in its membrane (here by DOPE-Atto 655) is proportional to

the square of its diameter. In brief, the integrated intensity of a liposome labeled in its membrane (here by DOPE-Atto 655) is proportional to the total number of lipids in the liposome, therefore the total liposome surface area, and the square of its diameter assuming a spherical shape. We were able to confirm that liposomes with diameters below the diffraction limited retained a spherical shape upon tethering on a surface using fluorescence energy transfer at the contact area between liposome and surface (Bendix et al., 2009). A conversion from liposome membrane intensity to physical size was therefore possible using a calibration sample of extruded small unilamellar vesicles with a controlled and narrow size distribution whose mean size was obtained by DLS (Kunding et al., 2008).

Data analysis, log-log representation and quantification of curvature sensing abilities

Images were acquired on a Leica TCS-SP5 inverted confocal microscope using a HCX PL APO oil immersion objective (100x magnification, 1.46 NA) (Leica, Wetzlar, Germany). CALM ANTH-Alexa488 and DOPE-Atto655 were excited by a 488nm and 655nm laserline, and emissions were filtered out in the range of 498nm – 600nm and 643nm – 750nm respectively. Images were acquired in the format 1024x1024 pixels, each pixel corresponding to 50.5nm sample length, bit-depth of 16, and a scanspeed of 400Hz. All experiments were performed in a 10mM Tris pH 7.4, 170mM NaCl, 0.05mM TCEP buffer. For all samples, liposomes were imaged before the addition of protein to ensure that the binding molecule did not affect liposome morphology or fluorescence.

Images were analyzed using a specifically developed routine in Igor Pro 5.05 (Wavemetrics) for extracting single particle positions, background

corrected integrated fluorescence intensities and identifying co-localised protein and liposome signals, as described previously (Hatzakis et al., 2009). We previously showed that recruitment by membrane curvature follows a power law, and quantified the recruitment potency as the slope of a straight-line fit to a log-log representation of protein density vs. liposome diameter. In this representation of the data a more negative slope represents a stronger recruitment by membrane curvature.

Transmission electron microscopy

Cells were fixed by addition of 5% glutaraldehyde (GA) / 4% paraformaldehyde (PFA) in 0.1M sodium cacodylate, pH 7.2 to an equal volume of tissue culture medium for 2min at 37°C. This solution was replaced with 2.5% GA / 2% PFA in 0.1M sodium cacodylate buffer, pH 7.2, and the cells were scraped from the dish, pelleted and fixed for 1hr at 20°C. The cell pellet was washed with 0.1M sodium cacodylate buffer, pH 7.2, and post-fixed with 1% osmium tetroxide in 0.1M sodium cacodylate buffer, pH 7.2, for 1hr. The pellet was washed with 0.05M sodium maleate buffer, pH 5.2, and stained *en bloc* with 0.5% uranyl acetate in 0.05M sodium maleate buffer for 1hr. The cell pellets were dehydrated in ethanol, exchanged into 1,2-epoxy propane and embedded in Araldite CY212 resin (Agar Scientific). Ultrathin sections were cut using a diamond knife mounted on an ultramicrotome (Reichert Ultracut S; Leica), collected onto formvar/carbon-coated EM grids and stained with uranyl acetate and lead citrate (Reynolds, 1963). The sections were observed in a CM 100 TEM (Philips) at an operating voltage of 80kV. For analysis of clathrin coated profiles, cell pellets were randomly sectioned and oriented in the electron microscope. Grids

were systematically scanned and on average 150 images of each cell line were captured using a CCD camera (Megaview III; Olympus). These images were used to trace 100 individual clathrin coated profiles with a Wacom® Bamboo™ tablet and overlayed in Adobe® Illustrator® CS6.

Immunogold electron microscopy

Cells were fixed by addition of 8% PFA / 0.2% gluteraldehyde in 0.1M sodium cacodylate buffer, pH 7.2 to an equal volume of cell culture medium at 37°C. After 2min, the solution was removed and cells were fixed in 4% PFA/0.1% glutaraldehyde in 0.1M Sodium Cacodylate buffer, pH 7.2, for 1hr at 20°C. Cells were scraped, pelleted, embedded in 10% gelatin at 37°C, cooled on ice and infused with 1.7M sucrose/15% polyvinyl pyrolidone overnight at 4°C. The blocks were then frozen onto aluminium stubs in liquid nitrogen and ultrathin frozen sections were cut with a diamond knife in a cryo ultramicrotome (Ultracut UCT /EM FCS; Leica), collected with 1% methyl cellulose / 1.15M sucrose and mounted on formvar/carbon-coated TEM grids. Sections were immunolabelled with an anti-myc antibody (Upstate) followed by rabbit anti-mouse (DAKO) and protein A: 15nm colloidal gold (Utrecht University, The Netherlands). Images were captured using a CCD camera (Eagle 4K) on a Tecnai G2 Spirit BioTWIN TEM (FEI, Eindhoven) at an operating voltage of 80kV.

Immunofluorescence Imaging

Conventional immunofluorescence microscopy was performed with an Axioplan fluorescence microscope (Carl Zeiss Ltd) equipped with a 63x oil immersion objective. Images were captured with a Hamamatsu Orca-R2 C10600 camera

(Hamamatsu Photonics). Confocal laser scanning microscopy was performed with a Leica TCS SP8 (Leica microsystems) and a 100x oil immersion objective. Images displayed in figure S5 represent single focal planes. The uptake of EGF-Alexa488 and Tf-Alexa565 as displayed in Figure 6 was analyzed in the total volume of the imaged cells by using z-stacks with 10 individual sections that covered a total thickness of 5-6µm. Subsequently, all sections were superimposed into one single maximum projection. To achieve optimal imaging, a frame averaging of 3 was used. No further image processing was applied.

STED (Stimulated Emission Depletion) super resolution microscopy.

Samples were prepared as described in main experimental procedures for standard immunofluorescent microscopy but to avoid non-specific staining due to high laser power excitation the nuclear staining was omitted. Prior to mounting, 0,2µm TetraSpeck fluorescent microspheres (Invitrogen) were diluted 1:500 in water, added to samples and incubated for 10 min. The spheres served as a size standard and as a control of fluorescence intensity among samples. For microscopy, a Leica TCS SP8 gSTED equipped with a 100x/1.4 Oil STED Orange lens and a 592 nm depletion laser was used. In brief, confocal and STED images were collected sequentially with a HyD detector at a scan speed of 400 lines/s, a pixel size of 20 nm (960x960 pixel), and a line average of 2. In the STED mode, a pulsed white light laser tuned to 488 nm was used for excitation and the emission signal was collected between 491 nm and 547 nm. To improve the lateral resolution gating between 0.6 ns and 6 ns was performed. The resulting images were imported into Photoshop (Adobe), adjusted equally for intensity

and background and mounted into the final figures without any further image correction.

For quantification, the STED images were first deconvolved with Huygens Professional 14.10 (Scientific Volume Imaging) using a theoretical PSF. The deconvolved STED image was then used to determine coated pit sizes. Coated pit size was quantified with custom code written in Python 2.7, using the scikit-image library (Stéfan van der Walt, Johannes L. Schönberger, Juan Nunez-Iglesias, François Boulogne, Joshua D. Warner, Neil Yager, Emmanuelle Gouillart, Tony Yu and the scikit-image contributors. scikit-image: Image processing in Python. PeerJ 2:e453 (2014) http://dx.doi.org/10.7717/peerj.453). In short, pits were first roughly detected with the Laplacian of Gaussian method (sigma and threshold values determined on control data and then kept constant). In the next step, a 2D circular Gaussian was fit on the pixel neighbourhood of each identified pit to determine its full width at half maximum (FWHM).

TIRF imaging of CALM-GFP and μ2-mCherry in live cells.

HEK293 cells were plated on clean coverslips on the same day as transfection and imaged 48hr later to allow several cell divisions and to give transfected cells time to adapt moderate protein overexpression (Taylor et al., 2012; Taylor et al., 2011). Only low expressing cells were imaged, the laser power at 488nm and 568nm and exposure times were fixed and only cells showing comparable similar signal to noise at coated pits for CALM-GFP and μ2-mCherry were analysed. To determine CCP maturation time in CALM knock-down cells HT1080 cells were transfected with either CALM (ACAGTTGGCAGACAGTTTA) or a non-targeting control siRNA followed by transfection with CALM-GFP and AP2 μ2-

mCherry. All image analysis used custom written scripts in Matlab (Taylor et al., 2011). To track fluorescently labelled clathrin coated pits the ‘a trous wavelet’ segmentation was used as described previously (Taylor et al., 2011) and track histories constructed using the nearest neighbour algorithm. To quantify the average dynamics of CALM-GFP and μ2-mCherry during clathrin coated pit nucleation the start of CALM-GFP track histories was used as a reference to align cohorts of fluorescence traces. This introduced a slight artefact (the ‘jump’ in the CALM-GFP fluorescence trace at t=0s, Figure 2A) since the first moment of detection of CALM-GFP will inevitably coincide with additive noise. However, the CALM-GFP and μ2-mCherry signals were well aligned for ~20sec preceding the first detection of CALM-GFP and there was no evidence to suggest that CALM-GFP arrived before or after μ2-Cherry at these time scales (1.0Hz) (the modal difference between first detection of CALM-GFP and μ2-mCherry was ‘0s’, 1963 events, 3 cells). The recruitment curves were well sampled and a faster acquisition rate would not have improved the analysis of clathrin coated pit nucleation. To align the fluorescence traces to the moment of budding the decrease in CALM-GFP was used as a reference. Briefly, the CALM-GFP fluorescence trace was convolved with a kernel adapted to detect the maximal decrease in fluorescence signal in CALM-GFP which was taken as ‘t = 0s’. Cohorts of CALM-GFP and μ2-mCherry fluorescence traces were then aligned and averaged.

References

Bendix, P.M., Pedersen, M.S., and Stamou, D. (2009). Quantification of nano-scale intermembrane contact areas by using fluorescence resonance energy transfer. P Natl Acad Sci USA *106*, 12341-12346.

Bhatia, V.K., Madsen, K.L., Bolinger, P.Y., Kunding, A., Hedegard, P., Gether, U., and Stamou, D. (2009). Amphipathic motifs in BAR domains are essential for membrane curvature sensing. EMBO J *28*, 3303-3314.

Borner, G.H.H., Antrobus, R., Hirst, J., Bhumbra, G.S., Kozik, P., Jackson, L.P., Sahlender, D.A., and Robinson, M.S. (2012). Multivariate proteomic profiling identifies novel accessory proteins of coated vesicles. J Cell Biol *197*, 141-160.

Christensen, S.M., Bolinger, P.Y., Hatzakis, N.S., Mortensen, M.W., and Stamou, D. (2012). Mixing subattolitre volumes in a quantitative and highly parallel manner with soft matter nanofluidics. Nat Nanotechnol *7*, 51-55.

Diederichs, K., and Karplus, P.A. (1997). Improved R-factors for diffraction data analysis in macromolecular crystallography. Nat Struct Biol *4*, 269-275.

Hatzakis, N.S., Bhatia, V.K., Larsen, J., Madsen, K.L., Bolinger, P.Y., Kunding, A.H., Castillo, J., Gether, U., Hedegard, P., and Stamou, D. (2009). How curved membranes recruit amphipathic helices and protein anchoring motifs. Nat Chem Biol *5*, 835-841.

Heller, H., Schaefer, M., and Schulten, K. (1993). Molecular-Dynamics Simulation of a Bilayer of 200 Lipids in the Gel and in the Liquid-Crystal Phases. J Phys Chem-Us *97*, 8343-8360.

Kozlov, M.M., Campelo, F., Liska, N., Chernomordik, L.V., Marrink, S.J., and McMahon, H.T. (2014). Mechanisms shaping cell membranes. Current opinion in cell biology *29*, 53-60.

Kunding, A.H., Mortensen, M.W., Christensen, S.M., and Stamou, D. (2008). A fluorescence-based technique to construct size distributions from single-object measurements: Application to the extrusion of lipid vesicles. Biophys J *95*, 1176-1188.

Reynolds, E.S. (1963). Use of Lead Citrate at High Ph as an Electron-Opaque Stain in Electron Microscopy. J Cell Biol *17*, 208-&.

Stachowiak, J.C., Schmid, E.M., Ryan, C.J., Ann, H.S., Sasaki, D.Y., Sherman, M.B., Geissler, P.L., Fletcher, D.A., and Hayden, C.C. (2012). Membrane bending by protein-protein crowding. Nat Cell Biol *14*, 944-+.

Taylor, M.J., Lampe, M., and Merrifield, C.J. (2012). A Feedback Loop between Dynamin and Actin Recruitment during Clathrin-Mediated Endocytosis. Plos Biology *10*.

Taylor, M.J., Perrais, D., and Merrifield, C.J. (2011). A high precision survey of the molecular dynamics of mammalian clathrin-mediated endocytosis. PLoS Biol *9*, e1000604.

Weiss, M.S., Metzner, H.J., and Hilgenfeld, R. (1998). Two non-proline cis peptide bonds may be important for factor XIII function. Febs Lett *423*, 291-296.
