# Supplementary material for: CALM Regulates Clathrin-Coated Vesicle Size and Maturation by Directly Sensing and Driving Membrane Curvature
Source: Dev Cell. 2015 Apr 20;33(2):163–75. doi: 10.1016/j.devcel.2015.03.002 (PMC4406947; doi:10.1016/j.devcel.2015.03.002)
Supplement: Document S2. Article plus Supplemental Information [file mmc2.pdf]

Developmental Cell

Article

CALM Regulates Clathrin-Coated Vesicle Size and Maturation by Directly Sensing and Driving Membrane Curvature

Graphical Abstract

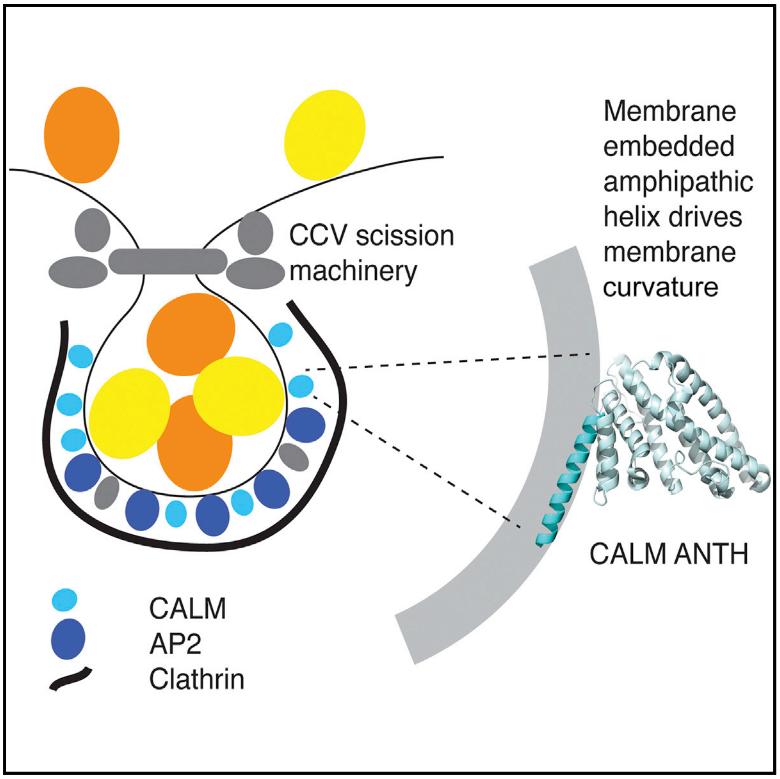

Highlights

- CALM loss increases size and frequency of early endocytic clathrin-coated structures
- Depletion of CALM slows endocytic clathrin-coated pit maturation and endocytic rate
- CALM possesses an N-terminal, membrane-curvature-sensing/driving amphipathic helix
- Clathrin-coated pit maturation is regulated by CALM's N-terminal amphipathic helix

Authors

Sharon E. Miller, Signe Mathiasen, ..., Stefan Höning, David J. Owen

Correspondence

sem50@cam.ac.uk (S.E.M.), djo30@cam.ac.uk (D.J.O.)

In Brief

Miller et al. demonstrate that depletion of the abundant endocytic protein CALM increases the diameter and percentage of early endocytic clathrin-coated structures (CCSs) while delaying CCS maturation and reducing endocytic rates. CALM's ability to influence these CCS properties depends on a membrane-inserting amphipathic helix, which senses and promotes membrane curvature.

Miller et al., 2015, Developmental Cell *33*, 163–175
April 20, 2015 ©2015 The Authors
http://dx.doi.org/10.1016/j.devcel.2015.03.002

Developmental Cell
Article

CellPress

CALM Regulates Clathrin-Coated Vesicle Size and Maturation by Directly Sensing and Driving Membrane Curvature

Sharon E. Miller,[1,*] Signe Mathiasen,[2] Nicholas A. Bright,[1] Fabienne Pierre,[3] Bernard T. Kelly,[1] Nikolay Kladt,[4] Astrid Schauss,[4] Christien J. Merrifield,[3] Dimitrios Stamou,[2] Stefan Höning,[5] and David J. Owen[1,*]
[1]Cambridge Institute for Medical Research and Department of Clinical Biochemistry, University of Cambridge, Cambridge Biomedical Campus, Wellcome Trust/MRC Building, Hills Road, Cambridge CB2 0XY, UK
[2]Bionanotechnology and Nanomedicine Laboratory, Department of Chemistry, University of Copenhagen, Universitetsparken 5, 2100 Copenhagen, Denmark
[3]Laboratoire d'Enzymologie et Biochimie Structurales, UPR3082 CNRS - Bat 34, Avenue de la Terrasse, 91198 Gif-sur-Yvette, France
[4]Cologne Excellence Cluster on Cellular Stress Responses in Aging-Associated Diseases (CECAD), Joseph-Stelzmann-Str. 26, 50931 Cologne, Germany
[5]Institute of Biochemistry I and Center for Molecular Medicine Cologne, University of Cologne, Joseph-Stelzmann-Str. 52, 50931 Cologne, Germany
*Correspondence: sem50@cam.ac.uk (S.E.M.), djo30@cam.ac.uk (D.J.O.)
http://dx.doi.org/10.1016/j.devcel.2015.03.002
This is an open access article under the CC BY license (http://creativecommons.org/licenses/by/4.0/).

SUMMARY

The size of endocytic clathrin-coated vesicles (CCVs) is remarkably uniform, suggesting that it is optimized to achieve the appropriate levels of cargo and lipid internalization. The three most abundant proteins in mammalian endocytic CCVs are clathrin and the two cargo-selecting, clathrin adaptors, CALM and AP2. Here we demonstrate that depletion of CALM causes a substantial increase in the ratio of "open" clathrin-coated pits (CCPs) to "necked"/"closed" CCVs and a doubling of CCP/CCV diameter, whereas AP2 depletion has opposite effects. Depletion of either adaptor, however, significantly inhibits endocytosis of transferrin and epidermal growth factor. The phenotypic effects of CALM depletion can be rescued by re-expression of wild-type CALM, but not with CALM that lacks a functional N-terminal, membrane-inserting, curvature-sensing/driving amphipathic helix, the existence and properties of which are demonstrated. CALM is thus a major factor in controlling CCV size and maturation and hence in determining the rates of endocytic cargo uptake.

INTRODUCTION

During clathrin-mediated endocytosis (CME), clathrin-coated pits (CCPs) are most frequently initiated by the formation of a small patch of the heterotetrameric AP2 clathrin adaptor complex and clathrin at sites of high PtdIns4,5P_2 concentration (Cocucci et al., 2012; Traub, 2011). If not aborted due to a lack of sufficient PtdIns4,5P_2 and/or cargo (Loerke et al., 2009), local membrane curvature steadily increases as more clathrin and a variety of additional clathrin adaptors are recruited until a bulbous membrane structure of ~80–100 nm diameter is formed on top of a membrane stalk that can undergo scission to generate a clathrin-coated vesicle (CCV) (Traub, 2011). The most abundant clathrin adaptors in endocytic CCVs isolated from tissue culture cells are CALM (clathrin assembly lymphoid myeloid leukemia protein) and AP2, each of which account for 30%–35% of the adaptors in a CCV (Blondeau et al., 2004; Borner et al., 2012). AP2 binds to and sorts general, often large, transmembrane cargo such as the transferrin receptor (TfR) into CCVs. CALM binds to and sorts the small R-SNAREs VAMPs 2, 3, and 8 (Koo et al., 2011; Miller et al., 2011). CALM and its neuronal specific homolog AP180, possess large, natively unstructured, C-terminal tails containing clathrin, AP2 α- and β2-appendage binding sites (Ford et al., 2001; Tebar et al., 1999; Traub, 2011), and a membrane-proximal, PtdIns4,5P_2-binding N-terminal ANTH stacked-helical domain, defined as residues 19–289 (Ford et al., 2001).

Several studies have shown that depletion of CALM in tissue culture cells and vertebrate neurons or of its orthologs in *D. melanogaster* and *C. elegans* causes an increase in CCP/CCV size and probably also a decrease in their uniformity of shape (Bao et al., 2005; Meyerholz et al., 2005; Nonet et al., 1999; Petralia et al., 2013; Zhang et al., 1998). These morphological alterations most likely result from changes in membrane curvature/sculpting. However, structural investigations had shown that CALM possessed neither a BAR domain nor an amphipathic helix that could influence membrane curvature, which was consistent with CALM apparently not affecting membrane curvature in biophysical assays (Boucrot et al., 2012; Ford et al., 2002; Stahelin et al., 2003). In an attempt to reconcile these contradictory observations of a key cellular process, we set out to determine whether, and if so, how CALM could directly affect CCP/CCV size and thus the cargo-carrying capacity because these are two key features in understanding the mechanism of endocytic CCP/CCV formation. Here we show that CALM possesses an N-terminal amphipathic helix

that regulates CCP/CCV size and maturation and hence endocytic rate.

RESULTS

Effect of CALM Depletion In Vivo

Endogenous CALM was depleted by siRNA and subsequent quantitated ultrastructural studies by electron microscopy (EM) and stimulated-emission depletion (STED) microscopy showed that CALM depletion results in substantially enlarged CCPs and CCVs compared to that of control cells (Figures 1, S1A, S1C, and S1E; see also Meyerholz et al., 2005), but no discernable change in the number of clathrin-coated structures (Figures 1B and S1A). The CCPs/CCVs in control cells have an average diameter of ~90 nm (Heuser, 1980). This value increased by ~2-fold upon depletion of CALM producing an ~4-fold increase in surface area, hence an ~8-fold increase in volume (Figures 1, S1C, and S1E). Importantly, our quantitation of CCP/CCV profiles of EM micrographs showed that "necked" CCPs/"closed" CCVs were the predominant fraction in control cells (~70%), whereas the shallow "open" CCPs accounted for only ~30% (Figures 1A and S1D). This was reversed in CALM-depleted cells, strongly suggestive of an alteration in the efficiency of CCP/CCV maturation, likely resulting from a reduced ability to curve/sculpt the plasma membrane (Figures 1A and S1D). In agreement with this, total internal reflection fluorescence (TIRF) microscopy revealed that the time taken to proceed from initiation of a CCP to its scission approximately doubles when CALM is depleted (Figures 2A and 2B).

We then compared the effects of expressing endogenous levels of full-length, siRNA-resistant CALM-wild-type (WT), CALM(PIP−), a mutant which has no PtdIns4,5P_2 binding due to the mutations Lys28Glu, Lys38Glu, and Lys40Glu (Ford et al., 2001) and is therefore cytosolic or CALM(VAMP−), which has no R-SNARE binding due to the mutation Met244Lys (Miller et al., 2011) in conjunction with endogenous CALM depletion (Figures S1A and S1B). Immunofluorescence indicated no obvious reduction in the number of punctate endocytic CCPs/CCVs (Figure S1A), whereas EM analysis of the CCP/CCV profiles showed that the expression of CALM-WT or CALM(VAMP−) rescued the relative ratios of open versus closed and sizes of CCPs/CCVs, whereas re-expression of CALM(PIP−) did not (Figures 1A and S1D). These data therefore indicate that CALM is not a major factor in CCP initiation, but rather that its presence strongly affects aspects of early to middle stages of CCP/CCV formation, and consequently influences the final CCV size. Because both CALM-WT and CALM(VAMP−) rescue phenotypes similarly, we further suggest that CALM's SNARE binding is not a checkpoint during CCP/CCV formation.

To substantiate our findings, we assessed the relative dynamics of AP2 and CALM during CCP formation in living cells with TIRF microscopy. These data showed that AP2 levels begin to decrease 15–20 s prior to the sharp decrease in the CALM levels that corresponds to the point when CCV scission occurs (Figures 2C–2F; see Cocucci et al., 2012; Loerke et al., 2009; Taylor et al., 2011; Traub, 2011). Within these 20 s prior scission, net membrane curvature must still increase to produce a necked and bulbous structure; hence, it is the level of CALM and not that of AP2 that correlates best with the increasing amount of positive curvature during CCP/CCV formation. CALM's binding partners FCHO, NECAP, and clathrin (Ritter et al., 2013; Tebar et al., 1999; Umasankar et al., 2012) also bind AP2 and so should be present in CCPs even when CALM is absent. This suggests that CALM's effects on CCP/CCV formation are not indirect through the action of these binding partners, but are consistent with a direct effect of CALM itself.

The N-Terminal 18 Residues of CALM Form an Amphipathic Helix

The impact of CALM on the size and shape of CCPs/CCVs led us to re-examine the structure of CALM to determine how it could directly affect membrane curvature. Inspection of the N-terminal residues 1–18 of CALM with the Heliquest server (http://heliquest.ipmc.cnrs.fr) suggested that this region could, in fact, assume an amphipathic helix (AH) (Figure 2G). Membrane-interacting AHs adopt their helical conformations when they attach to phospholipid membranes (Bhatia et al., 2009; Drin et al., 2007; Gallop et al., 2006); and indeed, liposome-based surface plasmon resonance (SPR) measurements showed that this potential N-terminal AH of CALM (we now term residues 1–18 as AH0, to be consistent with previous literature on other clathrin adaptors) contributed to the membrane binding of CALM. CALM ANTH bound to PtdIns4,5P_2 liposomes at physiological salt concentration (170 mM) with a K_D of 1.5 μM, whereas deletion of AH0 (CALM ANTH[ΔH0]), or the mutation of the key hydrophobic AH0 residues Leu6, Ile10, and Val17 to serines (CALM ANTH [H0mut]) both led to a decrease in affinity by ~8-fold (Figures 2H, 2I, and S2A). Furthermore, in the presence of PtdIns4,5P_2-containing liposomes, WT CALM ANTH showed a small but reproducible increase in α-helical content by circular dichroism (CD) (Figure S2B).

Comparison of our CALM ANTH:VAMP8 complex structure (Miller et al., 2011) with previous structures of the human and *Drosophila* CALM ANTH domains (Ford et al., 2001; Mao et al., 2001; Miller et al., 2011) showed that residues 5–14 can indeed form a short N-terminal helix that is connected to helix1 by a linker with the central axes of the helices diverging by ~40° at residues 14–17 (Figures 3A, 3E, S3A, and S3B). The structure of a C-terminally truncated form of the ANTH domain of CALM (CALM $ANTH_{(1–264)}$) (Table S1; Figures 3C, S3A, and S3C) although lacking the SNARE binding site, again showed residues 5–18 form a helix but this time extending helix1 by an additional four turns (Figures 3C, 3E, S3A, and S3C). The increased stabilizing energy that would arise from the continued α-helical H-bonding pattern seen in the structure of CALM $ANTH_{(1–264)}$ suggests that this extension of helix1 is the most likely conformation for residues 5–18 when the helix formation is induced by either membrane insertion or by fortuitous crystal packing. The failure to detect the existence and effects of this helix, in previous in vitro studies, likely results from this region's high proteolytic sensitivity.

AH0 of CALM Displays Membrane Curvature Sensing and Drives Membrane Deformation In Vitro

In other peripheral membrane proteins such as ArfGAP1, membrane-interacting amphipathic helices show membrane curvature sensing (MCS) and are referred to as ALPS (amphipathic lipid packing sensing) helices (Antonny, 2011; Bigay et al.,

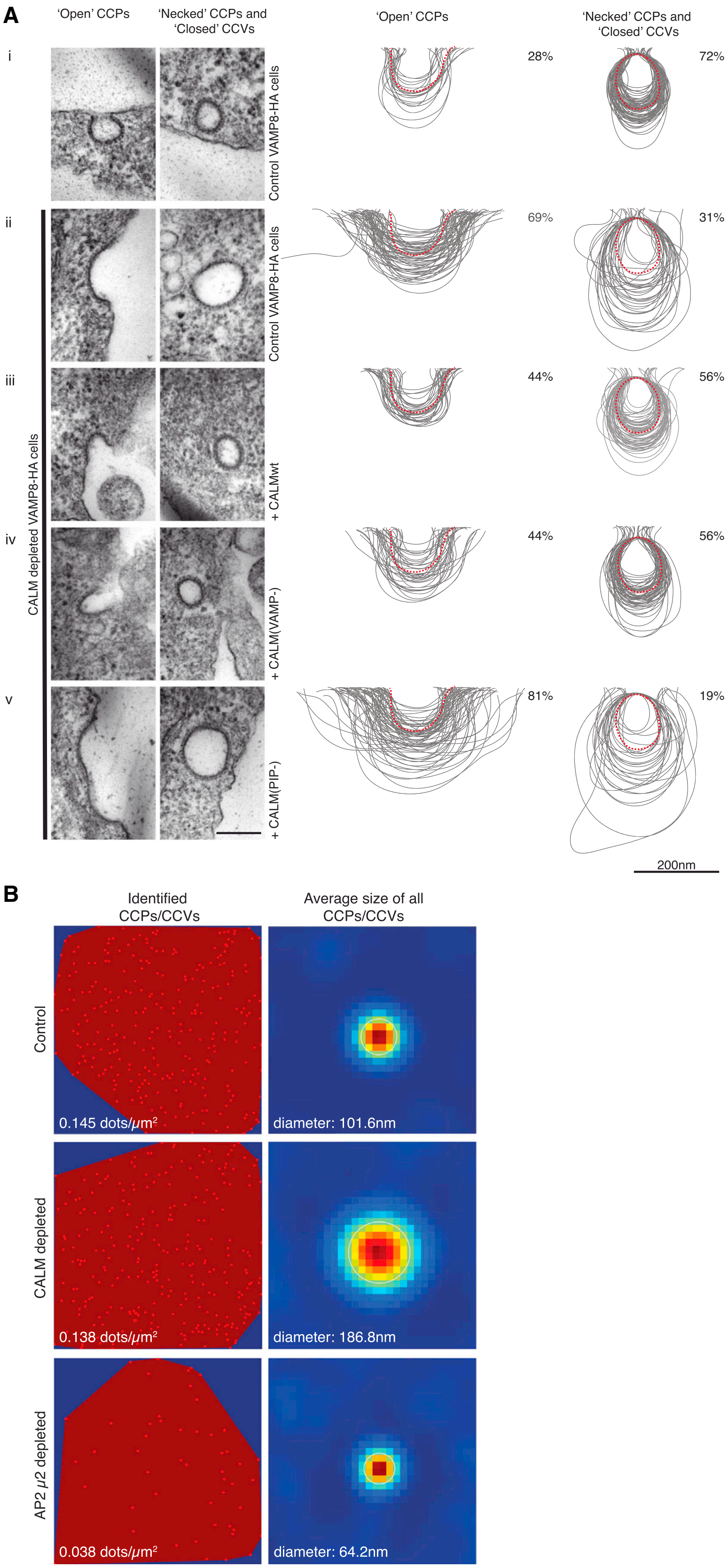

Figure 1. CALM Is Important for CCP and CCV Size Morphology

(A) All cells express an HA-tagged version of the endocytic SNARE VAMP8. The size variation of clathrin-coated structures, as a consequence of CALM depletion without and with re-expression of WT and mutant versions of CALM (ii–iv), compared to those found in control cells (i) are shown and quantified. Left-hand columns show representative images of open and necked/closed clathrin-coated structures for each cell line. Right-hand columns show the traces of 100 randomly selected clathrin-coated structures and the percentage (~150 images) of each type of structure adjacent to the traces. Dotted red line represents average size in control cells. Scale bars represent 200 nm.

(B) Cells were stained with antibodies for clathrin, AP2, and CALM and appropriate fluorochrome-conjugated secondary antibodies. After acquisition of confocal images, STED images were collected for clathin and processed for quantification, as described in Experimental Procedures. Images on the left display all identified clathrin-stained CCPs/CCVs in a polygonal area defined by the outermost objects and the calculated number/μm^2. Images on the right show a heat map of the overlay of all CCPs/CCVs identified (image on the left) and the calculated average diameter.

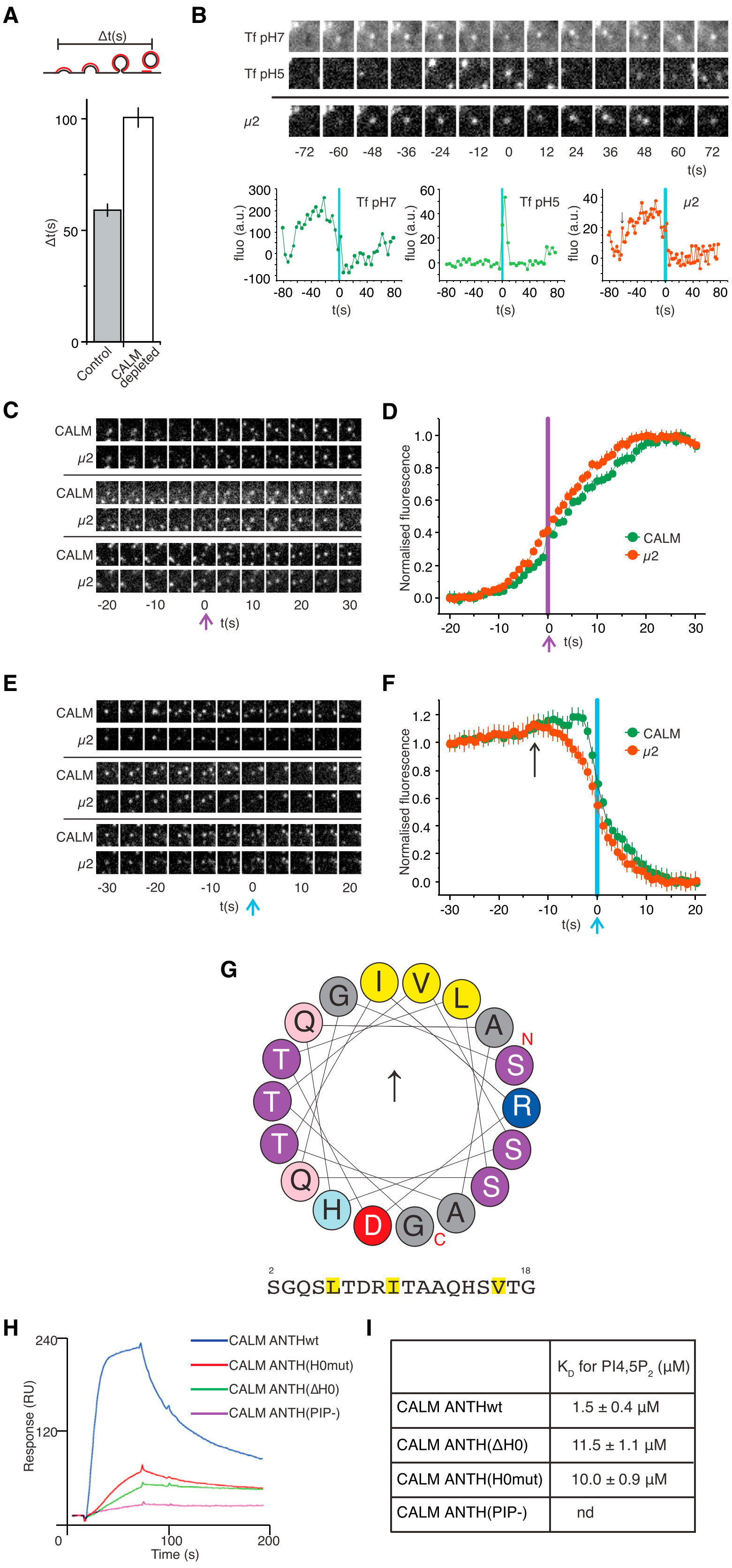

	K_D for PI4,5P$_2$ (μM)
CALM ANTHwt	1.5 ± 0.4 μM
CALM ANTH(ΔH0)	11.5 ± 1.1 μM
CALM ANTH(H0mut)	10.0 ± 0.9 μM
CALM ANTH(PIP-)	nd

Figure 2. CALM Affects CCV Maturation and Possesses an Amphipathic Helix that Contributes to Membrane Binding

(A) The time between first detection of a CCP and the first detected scission event at that CCP (Δts) was significantly extended in cells treated with CALM siRNA (control cells, Δt = 59 s, SEM = 3 s, 688 events, five cells; CALM siRNA, Δt = 100 s, SEM = 4 s, 590 events, five cells).

(B) An example CCP and scission event from a control cell. Images were acquired using TIRF microscopy and the pulsed pH protocol to detect single scission events. Images acquired at pH 7 (upper, Tf pH7) show a cluster of Transferrin-phluorin (Tf-phl) at a CCP. A scission event was detected when Tf-phl at the cluster became insulated from the externally imposed pH change (middle, Tf pH5, t = 0 s). Simultaneous detection of μ2-mCherry showed this CCP formed ~62 s before the detected scission event (lower, arrow in μ2-mCherry graph). The quantified fluorescence changes for this example CCP are plotted (graphs).

(C) Montages of example clathrin-coated pit nucleation events imaged using TIRF microscopy. The purple arrow indicates t = 0 s, the first frame in which the CALM-GFP (green) object was detected using automated tracking.

(D) Average CALM-GFP and μ2-mCherry (red) fluorescence traces of nucleating CCPs aligned to the first frame of detection of CALM-GFP (green). The average traces were normalized between the average of the first three fluorescence values and the peak fluorescence value for CALM and μ2, respectively. The fluorescence traces have a characteristic sigmoidal shape.

(E) Montages of example clathrin-coated pit budding events. The blue arrow indicates t = 0 s, the moment of maximum decrease in CALM-GFP fluorescence that corresponds to vesicle scission.

(F) Average CALM-GFP and μ2-mCherry at budding clathrin-coated pits. The average fluorescence traces were normalized to the average of the first three and last three fluorescence values for CALM and μ2, respectively. The μ2-mCherry fluorescence starts to decrease ~15 s before the CALM-GFP signal (black arrow).

(G) Heliquest server (http://heliquest.ipmc.cnrs.fr) helical wheel shows the orientation of the key hydrophobic residues (in yellow) and predicts the N terminus of CALM is an amphipathic helix with curvature-sensing properties. The hydrophobic moment points up toward the membrane and polar but mainly uncharged side chains are disposed laterally along where the surface the membrane would be. The negative charge points down away from the membrane.

(H) Liposome-based SPR sensorgrams showing binding of CALM ANTH-WT, CALM ANTH(ΔH0), CALM ANTH(H0mut), and CALM ANTH(PIP−) to PtdIns4,5P$_2$-containing liposomes at a protein concentration of 6 μM.

(I) K_D values for the binding of CALM ANTH domains, determined by liposome-based SPR (mean and SD of four independent measurements). Representative sensorgrams used for the measurements are shown in Figure S2A.

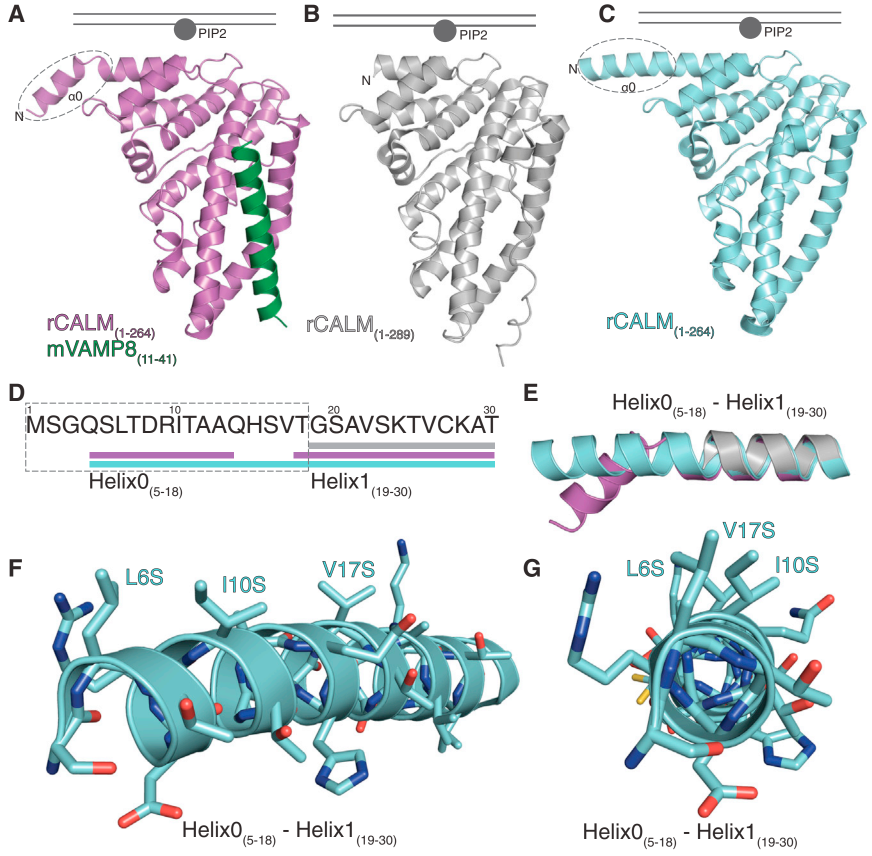

Figure 3. Structure of the CALM N-Terminal Amphipathic Helix (AH0)

CALM ANTH domain is in gray, truncated CALM $ANTH_{(1–264)}$ in pale blue, and the CALM ANTH: VAMP8 complex in purple/green.

(A–C) Three different structures of the first 289 residues of CALM with the binding site for $PtdIns4,5P_2$ and the proposed position of the membrane indicated. AH0 is indicated with a dashed ellipse.

(D and E) Positions (D) and ribbon representation (E) of α helices in the N-terminal 30 residues of the various structures. AH0 is indicated with a dashed rectangle.

(F and G) Angled (F) and face on (G) ribbon representations of the N terminus of CALM $ANTH_{(1–264)}$, highlighting the positions of the key AH0 hydrophobic residues Leu6, Ile10, and Val17 mutated to serines.

2003; Drin et al., 2007; Hatzakis et al., 2009). MCS arises from a combination of physicochemical effects including the insertion of the hydrophobic residue side chains of the helix into curvature-induced defects in membrane lipid packing and the interactions between the hydrophilic amino acid side chains of the helix and membrane phospholipid headgroups (Jensen et al., 2011).

When used in a sensitive microscope-based MCS assay on individual fluorescent liposomes (Lohr et al., 2009), CALM ANTH showed a preference for binding to small liposomes <100 nm diameter (around the size of closing CCPs), demonstrated by the enlarged population of smaller liposomes with higher protein densities (Figures 4A and S4A–S4G). CALM ANTH(ΔH0) showed a significantly reduced ability to sense curvature (Figures 4A and S4A–S4G), with a tendency to populate markedly lower protein densities. Taken together, our in vitro data indicate that CALM indeed possesses MCS capability, which is largely mediated by its AH0.

The deformation/tubulation of liposomes is often used as an indicator of membrane sculpting activity in vitro (Itoh et al., 2005; Peter et al., 2004; Stachowiak et al., 2012; van Weering et al., 2012). CALM ANTH-WT at 0.5 μM was extremely efficient at extensively tubulating 200 nm $PtdIns4,5P_2$-containing liposomes within 1 minute (Figures 4B, 4C, S4H, and S4I). CALM ANTH(ΔH0) and CALM ANTH(H0mut) at 0.5 μM were largely unable to deform the liposomes despite binding to them (Figure 4C). At concentrations of <0.1 μM, CALM ANTH-WT was unable to tubulate liposomes. The observed tubulation was not, however, reminiscent of the straight-sided tubules produced by BAR domains (Ford et al., 2002; Itoh et al., 2005; Peter et al., 2004; van Weering et al., 2012). It was instead "pearlized" (Stachowiak et al., 2012), with the pearls having approximately the same diameter (40–50 nm) (Figures 4B, S4H, and S4I) as the smallest endocytic CCVs found in neuronal synapses, which are enriched for CALM and AP180 (Blondeau et al., 2004). In previous liposome tubulation assays, CALM ANTH was used at concentrations of 4–20 μM (Ford et al., 2002; Stachowiak et al., 2012). At the concentration of CALM used here, we calculate a resulting surface coverage of ~16% (see Supplemental Experimental Procedures), a value close to that calculated for epsin, which forms smaller (17 nm diameter) tubules from liposomes (Kozlov et al., 2014). The value for CALM surface coverage in vitro resembles our estimate of its coverage in vivo of an endocytic CCV's surface area of ~17%. Our calculation (see Supplemental Experimental Procedures) shows the value for CALM will be much greater than that for epsin because it has been estimated that epsin constitutes less than 1% of clathrin adaptor content in endocytic CCVs (Borner et al., 2012). We conclude that the ANTH domain of CALM can drive pearlized liposome tubulation using its AH0.

We propose, therefore, that in vivo the targeting of CALM into a forming CCP through its preferential association with curved membranes should have two major effects: CALM will stabilize existing membrane curvature (by effectively shifting the thermodynamic equilibrium in favor of curved structures (Baumgart et al., 2011) and will also actively drive further membrane deformation/curvature toward that of endocytic CCVs (a diameter of ~90 nm, which corresponds to CALM's preferred binding curvature). Although we cannot definitively prove insertion of an unmodified AH0 of CALM into a membrane, the biochemical and biophysical properties of CALM's AH0 are consistent with it undergoing membrane insertion, as is accepted for similar AHs in other proteins (Antonny, 2011; Boucrot et al., 2012; Kozlov et al., 2014). This role for CALM is also consistent with its

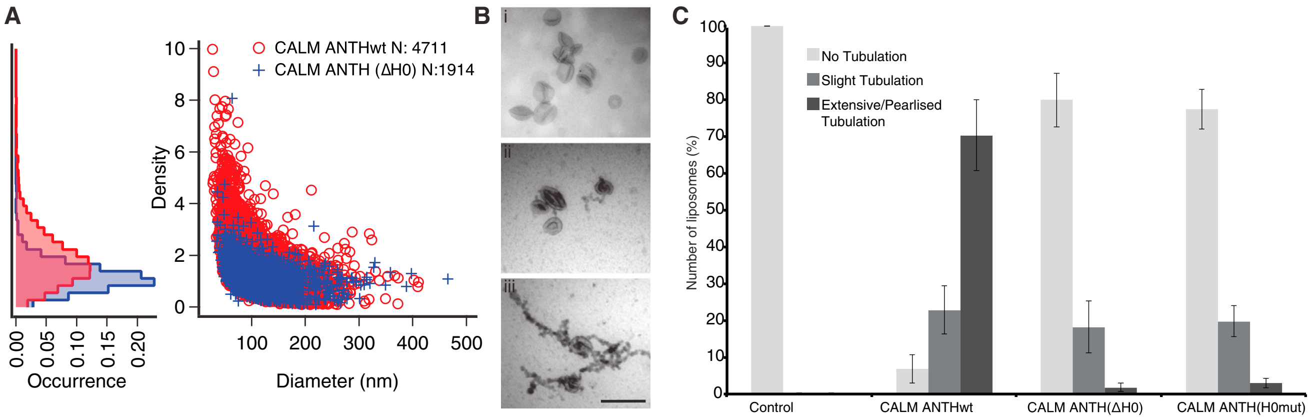

Figure 4. The AH0 of the CALM ANTH Domain Senses Membrane Curvature and Promotes Membrane Deformation

(A) Single liposome membrane curvature sensing assay showing CALM ANTHwt and CALM ANTH(ΔH0) density versus liposome diameter for PtdIns4,5P$_2$ containing liposomes. Protein concentration = 500 nM. The marginal histograms (left) reveal the occurrence of protein densities for CALM ANTH-WT and CALM ANTH(ΔH0).

(B) Tubulation of PtdIns4,5P$_2$-containing liposomes by 0.5 μM CALM ANTH proteins for 1 min. Sample electron micrographs showing (Bi) no, (Bii) slight, and (Biii) extensive pearlized tubulation typically seen. Scale bar represents 500 nm.

(C) CALM ANTH-WT causes extensive tubulation in ~70% of micrographs, whereas CALM ANTH(ΔH0) CALM ANTH(H0mut) do so only in ~2% and ~3%, respectively. Error bars show the SEM.

recruitment profile (Figures 2C–2F and Taylor et al., 2011) and as such would also help to concentrate CALM-binding endocytic R-SNAREs in CCVs.

In addition to helix insertion or wedging the other main contributor to protein driven membrane curvature comes from molecular crowding i.e., the lateral pressure exerted by colliding proteins that are attached on one side of a membrane (Boucrot et al., 2012; Kozlov et al., 2014; Stachowiak et al., 2012). The degree of membrane curvature driven by molecular crowding depends on protein size, concentration, and membrane affinity. CALM, due to its ~70 kDa molecular weight in combination with its >300 residue C-terminal unstructured region, should therefore contribute significantly to driving membrane curvature. The AH0 of CALM would also affect molecular crowding effects because AH0 not only increases the general membrane affinity of CALM, but also preferentially targets CALM to and thus concentrates it on the forming curved CCPs. CALM can thus initiate a positive amplification cascade of increasing membrane curvature starting from its initially low level in very early i.e., recently initiated CCPs to the final value in a CCV (corresponding to ~90 nm diameter) through multiple functions of its AH0. Taken together with the observation that CALM is highly abundant in CCVs (30%–35% of the clathrin adaptors (Borner et al., 2012)), these data allow us to propose that the AH0 of CALM controls the efficiency of endocytic CCP/CCV maturation and size by both sensing and driving membrane deformation.

CALM's AH0 Plays a Critical Role in Endocytic CCP Formation In Vivo

To confirm this hypothesis, we examined the effect of deleting AH0 or mutating AH0 on the ability of CALM to influence CCP/CCV properties in vivo by expressing endogenous levels of full-length, siRNA-resistant CALM(ΔH0) or CALM(H0mut) following endogenous CALM depletion (Figure S1B). Immunofluorescence indicated no obvious reduction in the number of punctate endocytic CCPs/CCVs (Figure S5A). However, despite CALM(ΔH0) or CALM(H0mut) being present in CCPs/CCVs and able to mediate normal trafficking of VAMP8 (Figure S5A), EM analysis showed that CCP/CCV morphology and the ratio of open to necked/closed structures resembled that of cells depleted of CALM (Figures 1A, 5, S1C, and S1D). That is, expression of CALM(ΔH0) or CALM(H0mut) failed to rescue the enlarged size phenotype and the normal ratio of open versus necked/closed clathrin-coated structures.

CALM Requires a Functional AH0 to Rescue the Reduction in Endocytic Rate Caused by CALM Depletion

In light of the major changes in CCP:CCV ratio, the time taken for CCP maturation and CCP/CCV size, we investigated the effect of CALM depletion on receptor/ligand internalization rates by measuring the steady state uptake rates at 37°C of radio-iodinated Tf and EGF. As shown in Figures 6A and 6B, CALM depletion caused ~50% reduction of both Tf and EGF uptake. This endocytosis defect was restored to normal in cells expressing siRNA-resistant CALM-WT, but not rescued in cells expressing CALM(ΔH0). Immunofluorescence correspondingly showed intracellular accumulation of Tf and EGF in control cells after 6 min endocytosis, whereas both ligands remain in punctae associated with the plasma membrane in cells depleted of endogenous CALM (Figures 6C and 6D). We thus conclude that the absence of CALM that possesses a functional AH0 delays CCV maturation (Figures 2A and 2B), resulting in a significant endocytic defect.

Depletion of AP2 and CALM Produces Opposite Effects on CCP/CCV Size In Vivo

CALM and AP2 depletion both caused a major reduction in Tf and in EGF internalization rates (Figures 6A and 6B; see also Huang et al., 2004). EM analysis however, showed in marked contrast to CALM depletion, AP2 depletion (of μ2) resulted in

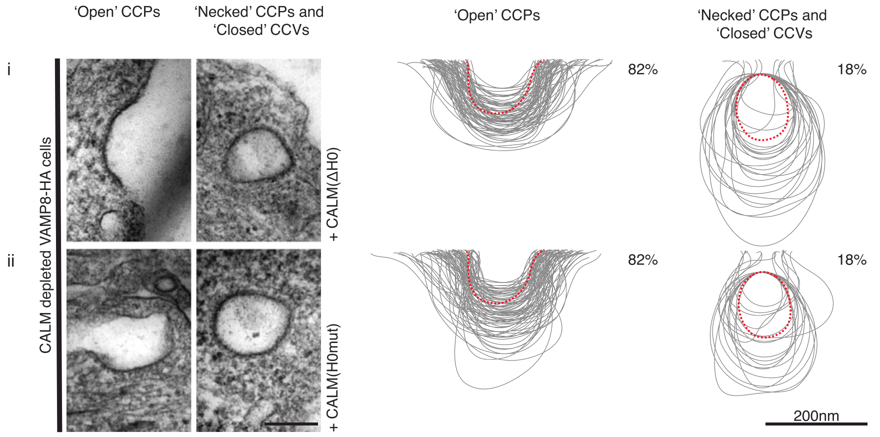

Figure 5. CALM with a Non-Functional AH0 Cannot Rescue CALM-Depleted CCP/CCV Profile Phenotypes

The size variation of clathrin-coated structures, as a consequence of CALM depletion with re-expression of mutant versions of (i) CALM(ΔH0) and (ii) CALM(H0mut) compared to those found in control cells as indicated by the dotted red line. Left-hand columns show representative images of open and necked/closed clathrin-coated structures for each cell line. Right-hand columns show the traces of 100 randomly selected clathrin-coated structures and the percentage (~150 images) of each type of structure adjacent to the traces. Scale bars represent 200 nm.

an ~12-fold decrease in the number of endocytic CCPs/CCVs (see also Motley et al., 2003) and a striking ~2-fold reduction in the diameter of the remaining clathrin-coated endocytic structures (note, this reduction in diameter is equivalent to a reduction in volume by ~90%) (Figures 1B, 7A, S1C–S1E). These AP2 depletion phenotypes were completely rescued by restoring the AP2 levels with re-expression of siRNA-resistant μ2 (Figure 7A). The clathrin adaptor complement of AP2-depleted endocytic structures should be dominated by CALM (the major PtdIns4,$5P_2$-binding endocytic adaptor remaining), suggesting that increasing amounts of CALM-WT in CCPs/CCVs results in them being very small (Figures 1B, 7A, and S1C). Unfortunately, we have found it impossible to accurately quantify the relative amount of CALM and clathrin in AP2-depleted cells because the number and size of endocytic CCPs/CCVs was so drastically reduced. In support of our proposal that the absence one of the two major clathrin adaptors causes the other one to dominate, quantification of STED images showed that depletion of CALM leads to a 2- to 3-fold increase in the AP2:clathrin ratio in clathrin-coated endocytic structures (data not shown).

In agreement with the proposal that CALM drives increasing membrane curvature, we also find that increased CALM expression (that is, endogenous CALM levels combined with similar levels of expression of siRNA-resistant CALM-WT) reduced CCP/CCV size and caused an increase in the number of necked/closed structures (Figures 7B, S1C, and S1D), whereas a combination of endogenous CALM and expression of CALM(ΔH0) expression did not (Figures 7B, S1C, and S1D). Unfortunately, it was not possible to create clonal cell lines stably expressing any higher levels of CALM (probably due to toxicity through inhibition of CME; Tebar et al., 1999) or to overexpress AP2 (which would require simultaneous overexpression of all four subunits). In conclusion, we propose that in vivo despite both being important for clathrin-mediated endocytosis, AP2 and CALM have antagonistic effects on CCP/CCV size and the ratio of open versus necked/closed structures, hence their relative levels are a major determinant in defining various CCP/CCV properties.

DISCUSSION

The initiation of CCP formation is generally believed to most often involve a pioneer complex of AP2/FCHO/Eps15 being recruited to a region of elevated PtdIns4,$5P_2$ (Cocucci et al., 2012; Hollopeter et al., 2014; Traub, 2011; Umasankar et al., 2014), which is then stabilized on the membrane by AP2 cargo being drawn in (Kelly et al., 2014; Loerke et al., 2009) and clathrin recruited and polymerized. Addition of more clathrin adaptors, including CALM, and of clathrin itself drives maturation of the CCP. Because CALM preferentially binds to curved membranes and CCP shape is a dynamic/equilibrium system, an increased amount of CALM in a CCP would tend to shift the net steady state of the structure toward increased membrane curvature; that is, CALM effectively drives membrane curvature. The increasing CCP curvature will result in CALM having an increased affinity for that region of the membrane and thus more CALM will be recruited to it i.e., a positive amplification cascade is established. Positive membrane curvature will increase until the CCP can be readily necked and then undergoes scission. The necked structure would likely resemble a single pearl such as those we see spontaneously produced on PtdIns4,$5P_2$-containing liposomes. The increase in the number of open CCPs and concomitant enlargement both resulting from the absence of fully functional CALM suggests a defect in the formation of late stage necked, bulbous CCPs. This defect would most likely be caused by the increased difficulty in necking a larger open CCP. Hence, it will take longer to neck/close the resulting larger CCP under these, suggesting that in the absence of CALM, CCP necking/scission tends toward being rate limiting. This proposal is supported by the observation that depletion of CALM caused a doubling of the time taken to reach a point at which a neck is formed that is small enough to allow vesicle scission to occur. That the AH0 of CALM plays a principle role in controlling of CCP/CCV size, curvature, and maturation can be inferred from the observation that neither expression of CALM(ΔH0) nor of CALM(H0mut) rescued the enlarged CCP/CCV phenotype, the CCV formation efficiency and the reduced endocytic rate. Intriguingly a similar increase in CCP size is seen when AP2 is replaced by AP2 lacking its α-appendage, the domain responsible for concentrating many CME proteins, including CALM, in CCPs (Aguet et al., 2013). These data suggest that a failure to recruit enough CALM into these AP2 positive CCPs could be the explanation for this phenotype.

Why should the depletion of CALM or AP2 have opposite effects on CCP/CCV morphology? Why should the presence of

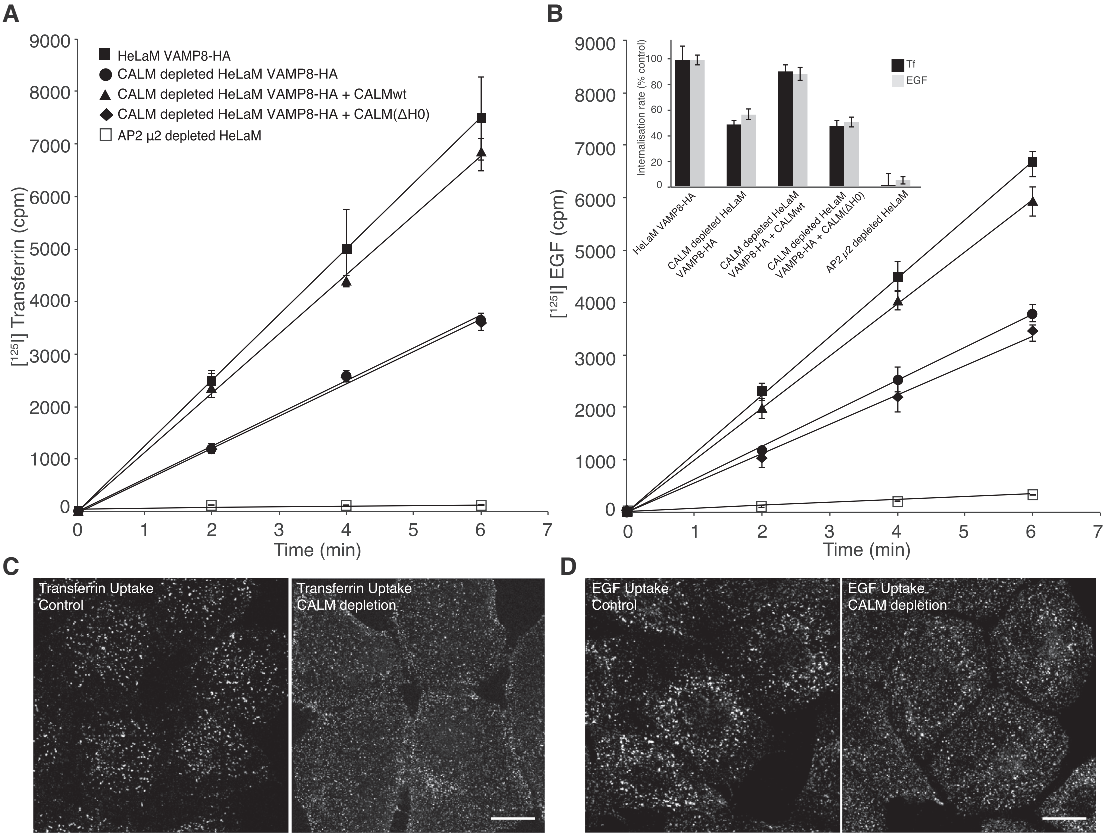

Figure 6. CALM's AH0 Directly Affects Rates of Endocytosis

(A and B) Internalization of (A) [^{125}I] transferrin (Tf) and (B) [^{125}I] EGF in HeLaM VAMP8-HA-expressing cells were measured for 2, 4, and 6 minutes. The amount of surface bound and internalized radioactivity was quantified and plotted against time. Each experiment was performed at least twice with triplicates for each time point. Error bars represent the SD of triplicates. Cells depleted of CALM showed an ~50% reduction in endocytosis of both Tf and EGF compared to that of control cells. This endocytic defect is rescued with the expression of siRNA resistant CALMwt, but not with CALM(ΔH0). Cells depleted of AP2 showed an ~90% reduction in endocytosis of both Tf and EGF compared to that of control cells. The bar chart (insert) shows a comparison of the internalization rate constants (k_e), which represent the linear regression coefficients. Those for the internalization by control HeLaM VAMP8-HA cells were set to 100%; all other values were put in relation to it. Error bars represent the SD.

(C and D) Immunofluorescence of cells after 6 min endocytosis of Tf-Alexa568 (C) or EGF-Alexa488 (D) by VAMP8-HA control and VAMP8-HA CALM-depleted cells. The images are maximum projections of ten focal z planes covering the whole volume of the imaged cells. Scale bar represents 15 µm.

larger CCPs/CCVs in CALM depleted cells with little reduction in the number of CCPs/CCVs reduce overall endocytic rate? We believe that both issues are linked to CALM's and AP2′s different structural/biophysical properties and the type of cargo they recognize. First, CALM possesses a curvature sensing/driving AH0 whereas AP2, as far as we know, does not. Second, the transmembrane cargoes bound by AP2 often have large extracellular portions (including TfR, EGFR) that, upon collision, will generate lateral pressure (Scheve et al., 2013; Stachowiak et al., 2012) causing membrane curvature in the opposite direction to that of inward budding favored by adaptor-mediated molecular crowding on the membrane's cytoplasmic face. This is analogous to a model put forward to explain the effect of GPI-linked cargo in COPII-coated vesicle formation (Copic et al., 2012). In a normal CCV, CALM will oppose this effect and so drive CCV closure. However, in endocytic CCPs where CALM has been depleted and therefore AP2 is predominant, there will be a need for a reduction in large cargo molecule density to reduce cargo collisions and so allow CCP closure. This can be achieved if no more cargo packed into these larger CCPs than is packed into ordinary sized CCPs. That this occurs is suggested by the similar and even slightly reduced intensity of TfR staining of the diffraction limited spots at the surface of control and CALM-depleted cells shown in Figure 6. However, it should also be noted that if the situation is such that in control cells most TfR is in internal compartments and any TfR delivered to the plasma membrane is immediately incorporated into CCPs as suggested (Hopkins et al., 1985), then larger CCPs resulting from CALM depletion cannot take up any more TfR than CCPs in control cells because no more TfR can be available to be taken up. In this case, endocytic rate will be determined by the rate at which the CCPs mature.

In contrast, the VAMP cargo of CALM has a negligible extracellular/luminal portion that does not oppose the positive membrane curvature driven by adaptor molecular crowding and the effect of CALM AH0 insertion. Thus, when CALM is the predominant adaptor (i.e., AP2 is depleted), the result is greatly reduced CCV size exacerbated by the fact that there is no large luminal

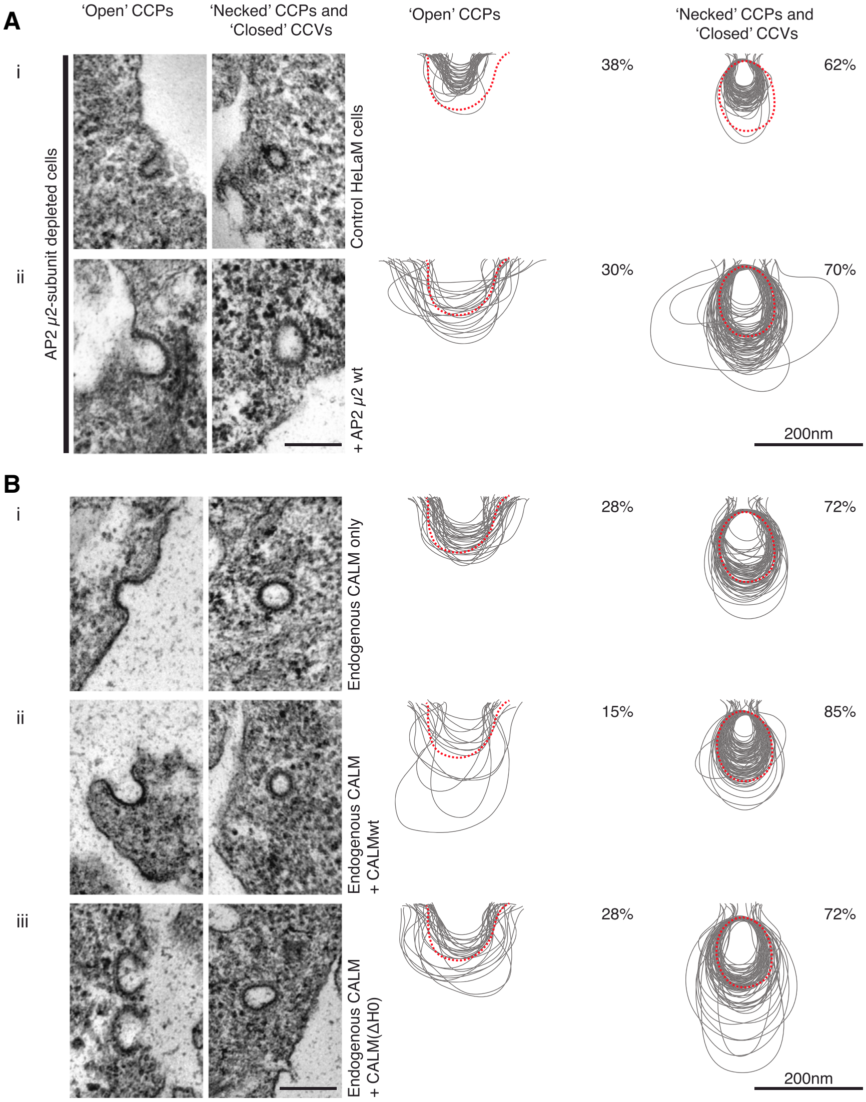

Figure 7. AP2 Depletion and CALM Overexpression Alter CCP/CCV Profile Phenotypes

Electron micrographs (left-hand columns) show a typical representation of an open and a necked/closed clathrin-coated structure for each cell line. Traces (right-hand columns) were made from 100 images and counts represented in percentages are of ~150 images for each condition. Dotted red line represents average size of CCPs/CCVs in control cells.

(A) The size variation of clathrin-coated structures are shown, and quantified AP2 (μ2 subunit)-depleted cells (Ai) show an obvious reduction in CCP/CCV size that can be rescued with the expression of an siRNA-resistant AP2-μ2 subunit (Aii).

(B) The size variation of clathrin-coated structures as a consequence of expressing siRNA-resistant CALM variants in addition to endogenous CALM (without siRNA treatment) (Bii and Biii). The levels of CALM were approximately double that in control cells as shown by western blotting (Figure S1). Attempts to create cell lines expressing higher levels of CALM expression failed presumably because higher levels of CALM expression are toxic. (Bi) As before, control cells show a greater percentage of necked/closed clathrin-coated structures (~70%) than open structures, and all profiles are within the size range previously obtained. Clathrin-coated structures in cells expressing endogenous and siRNA-resistant CALM-WT (Bii) were noticeably smaller than profiles of control profiles (note the size of the central cavity) (Bi) or of cells expressing endogenous and siRNA-resistant CALM(ΔH0) (Biii). In (Bii), there is also an increase in the percentage of necked/closed structures (85% as opposed to ~70% for Bi and Biii). Scale bars represent 200 nm.

domain cargo (AP2 binding) actively sorted into these structures to oppose CCP positive membrane curvature.

As CCP curvature approaches its final value and the bud neck forms, to avoid steric clashing/molecular crowding on the luminal side of the membrane, large YxxΦ and ExxxLL motif containing cargoes and their bound AP2 will tend to be excluded from this area and concentrate in the crown of the bulb (Saffarian and Kirchhausen, 2008). This would explain why AP2 levels appear to decline prior to scission when analyzed by TIRF microscopy (Aguet et al., 2013; Rappoport et al., 2003; Taylor et al., 2011). As a result of its sensing and driving positive membrane curvature and it not binding cargo that is excluded from the neck region of a CCP, CALM should continue to be recruited to the curved CCP membrane and should also not be partitioned into the crown. Hence, CALM's levels appear to continue to increase until scission. CALM may actually also aid in removing AP2 from near the neck as in the absence of AP2 cargo that has been excluded for steric reasons but with the positive curvature preferred by CALM, CALM should be able to more effectively compete with AP2 for binding of PtdIns4,5P_2 in this region (Honing et al., 2005).

Because an alteration in the clathrin adaptor complement alters the size and curvature of a CCP/CCV, we conclude that the presence of clathrin adaptors with the ability to drive membrane curvature by both insertion/wedging and molecular crowding plays a major role in defining CCP/CCV morphology. However, we cannot say from our studies what the contributions of membrane composition (Pinot et al., 2014) and the clathrin lattice (Dannhauser and Ungewickell, 2012; Heuser, 1980; Kelly et al., 2014; Meyerholz et al., 2005) are in driving and/or stabilizing CCP/CCV membrane curvature.

The coats of other transport vesicles contain and are attached to the membrane via small GTPases: Sar1:GTP (in COPII vesicles) or Arf1:GTP (in other CCVs and COPI vesicles), both of which possess AHs that display MCS, drive membrane curvature (Beck et al., 2008; Krauss et al., 2008; Lee et al., 2005) and can reduce membrane rigidity, thus facilitating membrane deformation (Settles et al., 2010). The only possible GTPase or Arf candidate in endocytic CCPs/CCVs is Arf6, but this is not found in significant amounts (Borner et al., 2012) and depletion

of Arf6 only marginally affects endocytosis (Montagnac et al., 2011). Hence, we propose that in endocytic CCPs, the AH0 of the abundant adaptor CALM assumes the role of a curvature sensor/driver similar to the AHs of small GTPases in other transport vesicles.

If one considers that nonsense mutations in CALM cause severe anemia and reduced life-span in mice (Klebig et al., 2003; Scotland et al., 2012), that complete deletion of the gene additionally causes other severe phenotypes resulting in >90% preweaning mortality (Ishikawa et al., 2014; Suzuki et al., 2012), that CALM is implicated in alterations in cognitive function with increasing age (Mengel-From et al., 2011), and finally that mutations in CALM are a risk factor for Alzheimer disease (Harold et al., 2009), the apparent inability of CALM depletion to affect endocytic rate in cultured cells previously reported in several studies was difficult to understand. However, by measuring the more physiological steady-state endocytic uptake rate at 37°C rather than using a 4°C block (Miller et al., 2011; Motley et al., 2003, 2006), we show that in fact CALM depletion does cause a marked reduction in endocytic rate of both EGF and transferrin. This is in at least partial agreement with a very recent study showing that a conditional CALM knockout specifically caused reduced transferrin uptake in mouse erythroblasts, although the authors suggest that CALM may be involved in a specialized endocytic mechanism in these cells (Ishikawa et al., 2014).

In conclusion, we have demonstrated that the N-terminal 18 residues of CALM form a previously unsuspected amphipathic helix (AH0) and that this structural element plays a central role in the ability of CALM to strongly influence CCP/CCV size, formation, rate of maturation, and thus endocytic uptake. We propose that it is the disruption of these properties in combination with effects on CALM's ability to drive the internalization of small R-SNAREs (Koo et al., 2011; Miller et al., 2011) that explains the pathophysiological effects caused by mutations in the CALM gene.

EXPERIMENTAL PROCEDURES

For additional information, including protein chemistry, reagent preparation, and constructs used, see the Supplemental Experimental Procedures.

Cell Culture

HeLaM-VAMP8-HA cells were selected under 0.5 mg/ml G418 and additionally transduced with myc-tagged, siRNA-resistant WT or mutant versions of CALM for the add-back experiments, using the retrovirus Phoenix system. The derived cells were selected with 0.2 mg/ml hygromycin B and 0.5 mg/ml G418. The highest level of myc-CALM expression was similar to the levels of endogenous CALM. Endogenous CALM was depleted with siRNA ACAGTTGGCAGACAGTTTA at 20 nM for 72 hr (Miller et al., 2011). Endogenous AP2-μ2 was depleted with siRNA AGTGGATGCCTTTCGGGGTCA (Motley et al., 2003) at 20 nM for 96 hr.

Immunofluorescence and Light Microscopy Imaging

Indirect immunofluorescence was performed after paraformaldehyde fixation and permeabilization with 0.1% Triton X-100 or 0.5% saponin. For imaging of transferrin endocytosis, the living cells were first serum-starved for 30 min, followed by exchange for medium supplemented with Alexa Fluor-conjugated transferrin (Invitrogen) for the indicated times. For TIRF microscopy, HEK293 cells were cultured and transfected using CALM-GFP and AP2 μ2-mCherry as detailed previously (Gage et al., 2005; Taylor et al., 2011). Cells were plated on clean coverslips on the same day as transfection and imaged 48 hr later to allow several cell divisions and to give transfected cells time to adapt moderate protein overexpression (Taylor et al., 2011, 2012). To determine CCP maturation time in CALM knockdown cells, HT1080 cells were transfected with either CALM or control siRNA followed by transfection with CALM-GFP and AP2 μ2-mCherry. The maturation time of CCPs was determined as described previously. For STED super resolution microscopy, samples were fixed and processed as described above. For microscopy, a Leica TCS SP8 gSTED equipped with a 100×/1.4 Oil STED Orange lens and a 592 nm depletion laser was used. Further details on STED imaging and quantification are summarized in the Supplemental Experimental Procedures.

Surface Plasmon Resonance-Based Analysis of Membrane Binding

Binding of recombinant CALM ANTH to membranes was determined with a BIAcore 3000 SPR biosensor (GE Healthcare). Details of the method are described elsewhere (Honing et al., 2005) and in the Supplemental Experimental Procedures.

Single Liposome Curvature Assay

Liposomes for single liposome curvature assay were prepared using a previously described lipid hydration method (Bhatia et al., 2009; Hatzakis et al., 2009). The size of liposomes was calibrated by combining confocal fluorescence microscopy and dynamic light-scattering measurement (Kunding et al., 2008). Single isolated liposomes of different diameters labeled by DOPE-Atto 655 were immobilized on a Neutravidin functionalized microscope glass surface. A total of 500 nM CALM ANTH-WT and CALM ANTH(ΔH0) labeled with Alexa 488 were allowed to bind the surface attached liposomes from solution. The fluorescently labeled protein and lipid were sequentially imaged, and the integrated intensities extracted and used to quantify the density of bound protein and the size of the individual liposomes.

All experiments were performed in a 10 mM Tris (pH 7.4), 170 mM NaCl, 0.05 mM TCEP buffer. For all samples, liposomes were imaged before the addition of protein to ensure that the binding molecule did not affect liposome morphology or fluorescence. Images were analyzed as described elsewhere (Hatzakis et al., 2009). We have previously shown that recruitment by membrane curvature follows a power law and quantified the recruitment potency as the slope of a straight-line fit to a log-log representation of protein density versus liposome diameter. In this representation of the data, a more negative slope represents a stronger recruitment by membrane curvature. Technical details are explained in full in the Supplemental Experimental Procedures.

Liposome Preparation and In Vitro Liposome Tubulation Assay

Brain polar lipid extract (BPLE) and PtdIns4,5P_2 (Avanti Polar Lipids) were hydrated in chloroform:methanol (4:1) supplemented with HCl; 0.5% PtdIns4,5P_2 BPLE was dried down with argon and rehydrated in 20 mM Tris (pH 7.4), 170 mM NaCl. The solution was extruded through a 200 nm polycarbonate membrane (Nuclepore) 21 times; 10 μl of 1 mg/ml 200 nm liposomes was used in a 100 μl reaction. Control samples contained no protein. CALM proteins (0.5 μM) were incubated for 1 min at 20°C. A formvar carbon-coated/glow-discharged EM grid was immediately placed on the surface of a 50:50 mix of the liposome protein solution and 9:1 2% methyl cellulose:2% uranyl acetate (to prevent air-drying artifacts of delicate structures as routinely used in the preparation of ultrathin frozen sections for immunolabelling; Slot and Geuze, 2007), incubated for 1 min, removed, wicked, and dried overnight. The grids were viewed (TEM, Phillips) and images were captured (Megaview III; Olympus). A new batch of liposomes and protein was used for six repeated experiments, resulting in final counts of several hundred liposomes for each condition. Liposome tubulation was categorized, counted, and plotted (Excel, Microsoft).

Transmission Electron Microscopy

Cells were fixed with 2.5% glutaraldehyde/2% paraformaldehyde, post-fixed with 1% osmium tetroxide, dehydrated, and embedded in epoxy resin. Randomly cut and orientated sections were stained (Reynolds, 1963) and CCPs were recorded with a charge-coupled device (CCD) camera (Megaview III; Olympus) on a CM100 transmission electron microscope (TEM; Philips). One hundred profiles were traced with a Wacom Bamboo tablet and overlaid using Adobe Illustrator CS6.

Immunogold Electron Microscopy

Cells were fixed with 0.1% glutaraldehyde / 4% paraformaldehyde, infused with 1.7 M sucrose/15% polyvinyl pyrolidone, and frozen in liquid nitrogen. Sections were cut in a cryo-ultramicrotome (Ultracut UCT/EM FCS; Leica), collected with 1% methyl cellulose/1.15 M sucrose, and immunolabelled with mouse anti-myc (Upstate) followed by rabbit anti-mouse (DAKO) and protein A: 15 nm colloidal gold (Utrecht University, The Netherlands). Images were captured using a CCD camera (Eagle 4K) on a Tecnai G2 Spirit BioTWIN TEM (FEI).

Internalization of [^{125}I] Transferrin and [^{125}I] Epidermal Growth Factor

The internalization of iodinated transferrin (Tf) and EGF was determined according to (Jiang et al., 2003) and (Huang et al., 2004). In brief, triplicates of cells were grown on 12-well dishes and incubated in either 2 μg/ml ^{125}I-labeled Tf or 1 ng/ml ^{125}I-labeled EGF at 37°C for 2, 4, and 6 minutes. Subsequently, the cells were placed on ice and washed. Thereafter, the cells were incubated twice in 0.2 M acetic acid (pH 2.6), 0.5 M NaCl for 5 min to remove the surface-bound ligand. Finally, the cells were solubilized and collected in 1 M NaOH. The acid-releasable radioactivity represented surface-bound ligands, whereas the acid-resistant fraction solubilized in NaOH represented the internalized fraction. The latter was plotted against time. The resulting linear regression coefficients represent the endocytic rate constants k_e.

SUPPLEMENTAL INFORMATION

Supplemental Information includes Supplemental Experimental Procedures, five figures, and one table and can be found with this article online at http://dx.doi.org/10.1016/j.devcel.2015.03.002.

ACKNOWLEDGMENTS

We thank the beamline scientists at Diamond Light Source and Stephen Graham for assistance with data collection. Nicole Liska for help and advice with liposomes. Tobias Bläske and Eva Cziudaj for expert technical assistance. Andrew Peden for the VAMP8-HA cell line, Margaret Robinson for the AP2-μ2 cell line, and Tim Dafforn for advice on CD. S.E.M. and D.J.O. are funded by a Wellcome Trust Fellowship (to D.J.O. no. 090909/Z). N.A.B. is funded by MRC grant MR/M010007/1, and S.H. is funded by a grant from the German Science Foundation (SFB 635, TP A3). D.S. and S.M. acknowledge financial support from the Lundbeck Foundation and the Danish Councils for Independent and Strategic Research. C.J.M. and F.P. were funded by the Fondation pour la Recherche Medicale.

Received: August 25, 2014
Revised: January 23, 2015
Accepted: March 1, 2015
Published: April 20, 2015

REFERENCES

Aguet, F., Antonescu, C.N., Mettlen, M., Schmid, S.L., and Danuser, G. (2013). Advances in analysis of low signal-to-noise images link dynamin and AP2 to the functions of an endocytic checkpoint. Dev. Cell *26*, 279–291.

Antonny, B. (2011). Mechanisms of membrane curvature sensing. Annu. Rev. Biochem. *80*, 101–123.

Bao, H., Daniels, R.W., MacLeod, G.T., Charlton, M.P., Atwood, H.L., and Zhang, B. (2005). AP180 maintains the distribution of synaptic and vesicle proteins in the nerve terminal and indirectly regulates the efficacy of Ca2+-triggered exocytosis. J. Neurophysiol. *94*, 1888–1903.

Baumgart, T., Capraro, B.R., Zhu, C., and Das, S.L. (2011). Thermodynamics and mechanics of membrane curvature generation and sensing by proteins and lipids. Annu. Rev. Phys. Chem. *62*, 483–506.

Beck, R., Sun, Z., Adolf, F., Rutz, C., Bassler, J., Wild, K., Sinning, I., Hurt, E., Brügger, B., Béthune, J., and Wieland, F. (2008). Membrane curvature induced by Arf1-GTP is essential for vesicle formation. Proc. Natl. Acad. Sci. USA *105*, 11731–11736.

Bhatia, V.K., Madsen, K.L., Bolinger, P.Y., Kunding, A., Hedegård, P., Gether, U., and Stamou, D. (2009). Amphipathic motifs in BAR domains are essential for membrane curvature sensing. EMBO J. *28*, 3303–3314.

Bigay, J., Gounon, P., Robineau, S., and Antonny, B. (2003). Lipid packing sensed by ArfGAP1 couples COPI coat disassembly to membrane bilayer curvature. Nature *426*, 563–566.

Blondeau, F., Ritter, B., Allaire, P.D., Wasiak, S., Girard, M., Hussain, N.K., Angers, A., Legendre-Guillemin, V., Roy, L., Boismenu, D., et al. (2004). Tandem MS analysis of brain clathrin-coated vesicles reveals their critical involvement in synaptic vesicle recycling. Proc. Natl. Acad. Sci. USA *101*, 3833–3838.

Borner, G.H.H., Antrobus, R., Hirst, J., Bhumbra, G.S., Kozik, P., Jackson, L.P., Sahlender, D.A., and Robinson, M.S. (2012). Multivariate proteomic profiling identifies novel accessory proteins of coated vesicles. J. Cell Biol. *197*, 141–160.

Boucrot, E., Pick, A., Çamdere, G., Liska, N., Evergren, E., McMahon, H.T., and Kozlov, M.M. (2012). Membrane fission is promoted by insertion of amphipathic helices and is restricted by crescent BAR domains. Cell *149*, 124–136.

Cocucci, E., Aguet, F., Boulant, S., and Kirchhausen, T. (2012). The first five seconds in the life of a clathrin-coated pit. Cell *150*, 495–507.

Copic, A., Latham, C.F., Horlbeck, M.A., D'Arcangelo, J.G., and Miller, E.A. (2012). ER cargo properties specify a requirement for COPII coat rigidity mediated by Sec13p. Science *335*, 1359–1362.

Dannhauser, P.N., and Ungewickell, E.J. (2012). Reconstitution of clathrin-coated bud and vesicle formation with minimal components. Nat. Cell Biol. *14*, 634–639.

Drin, G., Casella, J.F., Gautier, R., Boehmer, T., Schwartz, T.U., and Antonny, B. (2007). A general amphipathic α-helical motif for sensing membrane curvature. Nat. Struct. Mol. Biol. *14*, 138–146.

Ford, M.G., Pearse, B.M., Higgins, M.K., Vallis, Y., Owen, D.J., Gibson, A., Hopkins, C.R., Evans, P.R., and McMahon, H.T. (2001). Simultaneous binding of PtdIns(4,5)P2 and clathrin by AP180 in the nucleation of clathrin lattices on membranes. Science *291*, 1051–1055.

Ford, M.G.J., Mills, I.G., Peter, B.J., Vallis, Y., Praefcke, G.J.K., Evans, P.R., and McMahon, H.T. (2002). Curvature of clathrin-coated pits driven by epsin. Nature *419*, 361–366.

Gage, R.M., Matveeva, E.A., Whiteheart, S.W., and von Zastrow, M. (2005). Type I PDZ ligands are sufficient to promote rapid recycling of G protein-coupled receptors independent of binding to N-ethylmaleimide-sensitive factor. J. Biol. Chem. *280*, 3305–3313.

Gallop, J.L., Jao, C.C., Kent, H.M., Butler, P.J., Evans, P.R., Langen, R., and McMahon, H.T. (2006). Mechanism of endophilin N-BAR domain-mediated membrane curvature. EMBO J. *25*, 2898–2910.

Harold, D., Abraham, R., Hollingworth, P., Sims, R., Gerrish, A., Hamshere, M.L., Pahwa, J.S., Moskvina, V., Dowzell, K., Williams, A., et al. (2009). Genome-wide association study identifies variants at CLU and PICALM associated with Alzheimer's disease. Nat. Genet. *41*, 1088–1093.

Hatzakis, N.S., Bhatia, V.K., Larsen, J., Madsen, K.L., Bolinger, P.Y., Kunding, A.H., Castillo, J., Gether, U., Hedegård, P., and Stamou, D. (2009). How curved membranes recruit amphipathic helices and protein anchoring motifs. Nat. Chem. Biol. *5*, 835–841.

Heuser, J. (1980). Three-dimensional visualization of coated vesicle formation in fibroblasts. J. Cell Biol. *84*, 560–583.

Hollopeter, G., Lange, J.J., Zhang, Y., Vu, T.N., Gu, M., Ailion, M., Lambie, E.J., Slaughter, B.D., Unruh, J.R., Florens, L., and Jorgensen, E.M. (2014). The membrane-associated proteins FCHo and SGIP are allosteric activators of the AP2 clathrin adaptor complex. eLife *3*, 3.

Honing, S., Ricotta, D., Krauss, M., Spate, K., Spolaore, B., Motley, A., Robinson, M., Robinson, C., Haucke, V., and Owen, D.J. (2005). Phosphatidylinositol-(4,5)-bisphosphate regulates sorting signal recognition by the clathrin-associated adaptor complex AP2 (vol 19, pg 577, 2005). Mol. Cell *19*, 577–577.

Hopkins, C.R., Miller, K., and Beardmore, J.M. (1985). Receptor-mediated endocytosis of transferrin and epidermal growth factor receptors: a comparison of constitutive and ligand-induced uptake. J. Cell Sci. Suppl. *3*, 173–186.

Huang, F., Khvorova, A., Marshall, W., and Sorkin, A. (2004). Analysis of clathrin-mediated endocytosis of epidermal growth factor receptor by RNA interference. J. Biol. Chem. *279*, 16657–16661.

Ishikawa, Y., Maeda, M., Pasham, M., Aguet, F., Tacheva-Grigorova, S.K., Masuda, T., Yi, H., Lee, S.U., Xu, J., Teruya-Feldstein, J., et al. (2014). Role of the clathrin adaptor PICALM in normal hematopoiesis and polycythemia vera pathophysiology. Haematologica.

Itoh, T., Erdmann, K.S., Roux, A., Habermann, B., Werner, H., and De Camilli, P. (2005). Dynamin and the actin cytoskeleton cooperatively regulate plasma membrane invagination by BAR and F-BAR proteins. Dev. Cell *9*, 791–804.

Jensen, M.B., Bhatia, V.K., Jao, C.C., Rasmussen, J.E., Pedersen, S.L., Jensen, K.J., Langen, R., and Stamou, D. (2011). Membrane curvature sensing by amphipathic helices: a single liposome study using α-synuclein and annexin B12. J. Biol. Chem. *286*, 42603–42614.

Jiang, X., Huang, F., Marusyk, A., and Sorkin, A. (2003). Grb2 regulates internalization of EGF receptors through clathrin-coated pits. Mol. Biol. Cell *14*, 858–870.

Kelly, B.T., Graham, S.C., Liska, N., Dannhauser, P.N., Höning, S., Ungewickell, E.J., and Owen, D.J. (2014). Clathrin adaptors. AP2 controls clathrin polymerization with a membrane-activated switch. Science *345*, 459–463.

Klebig, M.L., Wall, M.D., Potter, M.D., Rowe, E.L., Carpenter, D.A., and Rinchik, E.M. (2003). Mutations in the clathrin-assembly gene Picalm are responsible for the hematopoietic and iron metabolism abnormalities in fit1 mice. Proc. Natl. Acad. Sci. USA *100*, 8360–8365.

Koo, S.J., Markovic, S., Puchkov, D., Mahrenholz, C.C., Beceren-Braun, F., Maritzen, T., Dernedde, J., Volkmer, R., Oschkinat, H., and Haucke, V. (2011). SNARE motif-mediated sorting of synaptobrevin by the endocytic adaptors clathrin assembly lymphoid myeloid leukemia (CALM) and AP180 at synapses. Proc. Natl. Acad. Sci. USA *108*, 13540–13545.

Kozlov, M.M., Campelo, F., Liska, N., Chernomordik, L.V., Marrink, S.J., and McMahon, H.T. (2014). Mechanisms shaping cell membranes. Curr. Opin. Cell Biol. *29*, 53–60.

Krauss, M., Jia, J.Y., Roux, A., Beck, R., Wieland, F.T., De Camilli, P., and Haucke, V. (2008). Arf1-GTP-induced tubule formation suggests a function of Arf family proteins in curvature acquisition at sites of vesicle budding. J. Biol. Chem. *283*, 27717–27723.

Kunding, A.H., Mortensen, M.W., Christensen, S.M., and Stamou, D. (2008). A fluorescence-based technique to construct size distributions from single-object measurements: application to the extrusion of lipid vesicles. Biophys. J. *95*, 1176–1188.

Lee, M.C., Orci, L., Hamamoto, S., Futai, E., Ravazzola, M., and Schekman, R. (2005). Sar1p N-terminal helix initiates membrane curvature and completes the fission of a COPII vesicle. Cell *122*, 605–617.

Loerke, D., Mettlen, M., Yarar, D., Jaqaman, K., Jaqaman, H., Danuser, G., and Schmid, S.L. (2009). Cargo and dynamin regulate clathrin-coated pit maturation. PLoS Biol. *7*, e57.

Lohr, C., Kunding, A.H., Bhatia, V.K., and Stamou, D. (2009). Constructing size distributions of liposomes from single-object fluorescence measurements. Methods Enzymol. *465*, 143–160.

Mao, Y., Chen, J., Maynard, J.A., Zhang, B., and Quiocho, F.A. (2001). A novel all helix fold of the AP180 amino-terminal domain for phosphoinositide binding and clathrin assembly in synaptic vesicle endocytosis. Cell *104*, 433–440.

Mengel-From, J., Christensen, K., McGue, M., and Christiansen, L. (2011). Genetic variations in the CLU and PICALM genes are associated with cognitive function in the oldest old. Neurobiol. Aging *32*, 554.e557–11.

Meyerholz, A., Hinrichsen, L., Groos, S., Esk, P.C., Brandes, G., and Ungewickell, E.J. (2005). Effect of clathrin assembly lymphoid myeloid leukemia protein depletion on clathrin coat formation. Traffic *6*, 1225–1234.

Miller, S.E., Sahlender, D.A., Graham, S.C., Höning, S., Robinson, M.S., Peden, A.A., and Owen, D.J. (2011). The molecular basis for the endocytosis of small R-SNAREs by the clathrin adaptor CALM. Cell *147*, 1118–1131.

Montagnac, G., de Forges, H., Smythe, E., Gueudry, C., Romao, M., Salamero, J., and Chavrier, P. (2011). Decoupling of activation and effector binding underlies ARF6 priming of fast endocytic recycling. Curr. Biol. *21*, 574–579.

Motley, A., Bright, N.A., Seaman, M.N., and Robinson, M.S. (2003). Clathrin-mediated endocytosis in AP-2-depleted cells. J. Cell Biol. *162*, 909–918.

Motley, A.M., Berg, N., Taylor, M.J., Sahlender, D.A., Hirst, J., Owen, D.J., and Robinson, M.S. (2006). Functional analysis of AP-2 alpha and mu2 subunits. Mol. Biol. Cell *17*, 5298–5308.

Nonet, M.L., Holgado, A.M., Brewer, F., Serpe, C.J., Norbeck, B.A., Holleran, J., Wei, L., Hartwieg, E., Jorgensen, E.M., and Alfonso, A. (1999). UNC-11, a Caenorhabditis elegans AP180 homologue, regulates the size and protein composition of synaptic vesicles. Mol. Biol. Cell *10*, 2343–2360.

Peter, B.J., Kent, H.M., Mills, I.G., Vallis, Y., Butler, P.J., Evans, P.R., and McMahon, H.T. (2004). BAR domains as sensors of membrane curvature: the amphiphysin BAR structure. Science *303*, 495–499.

Petralia, R.S., Wang, Y.X., Indig, F.E., Bushlin, I., Wu, F., Mattson, M.P., and Yao, P.J. (2013). Reduction of AP180 and CALM produces defects in synaptic vesicle size and density. Neuromolecular Med. *15*, 49–60.

Pinot, M., Vanni, S., Pagnotta, S., Lacas-Gervais, S., Payet, L.A., Ferreira, T., Gautier, R., Goud, B., Antonny, B., and Barelli, H. (2014). Lipid cell biology. Polyunsaturated phospholipids facilitate membrane deformation and fission by endocytic proteins. Science *345*, 693–697.

Rappoport, J.Z., Taha, B.W., Lemeer, S., Benmerah, A., and Simon, S.M. (2003). The AP-2 complex is excluded from the dynamic population of plasma membrane-associated clathrin. J. Biol. Chem. *278*, 47357–47360.

Reynolds, E.S. (1963). The use of lead citrate at high pH as an electron-opaque stain in electron microscopy. J. Cell Biol. *17*, 208–212.

Ritter, B., Murphy, S., Dokainish, H., Girard, M., Gudheti, M.V., Kozlov, G., Halin, M., Philie, J., Jorgensen, E.M., Gehring, K., and McPherson, P.S. (2013). NECAP 1 regulates AP-2 interactions to control vesicle size, number, and cargo during clathrin-mediated endocytosis. PLoS Biol. *11*, e1001670.

Saffarian, S., and Kirchhausen, T. (2008). Differential evanescence nanometry: live-cell fluorescence measurements with 10-nm axial resolution on the plasma membrane. Biophys. J. *94*, 2333–2342.

Scheve, C.S., Gonzales, P.A., Momin, N., and Stachowiak, J.C. (2013). Steric pressure between membrane-bound proteins opposes lipid phase separation. J. Am. Chem. Soc. *135*, 1185–1188.

Scotland, P.B., Heath, J.L., Conway, A.E., Porter, N.B., Armstrong, M.B., Walker, J.A., Klebig, M.L., Lavau, C.P., and Wechsler, D.S. (2012). The PICALM protein plays a key role in iron homeostasis and cell proliferation. PLoS ONE *7*, e44252.

Settles, E.I., Loftus, A.F., McKeown, A.N., and Parthasarathy, R. (2010). The vesicle trafficking protein Sar1 lowers lipid membrane rigidity. Biophys. J. *99*, 1539–1545.

Slot, J.W., and Geuze, H.J. (2007). Cryosectioning and immunolabeling. Nat. Protoc. *2*, 2480–2491.

Stachowiak, J.C., Schmid, E.M., Ryan, C.J., Ann, H.S., Sasaki, D.Y., Sherman, M.B., Geissler, P.L., Fletcher, D.A., and Hayden, C.C. (2012). Membrane bending by protein-protein crowding. Nat. Cell Biol. *14*, 944–949.

Stahelin, R.V., Long, F., Peter, B.J., Murray, D., De Camilli, P., McMahon, H.T., and Cho, W. (2003). Contrasting membrane interaction mechanisms of AP180 N-terminal homology (ANTH) and epsin N-terminal homology (ENTH) domains. J. Biol. Chem. *278*, 28993–28999.

Suzuki, M., Tanaka, H., Tanimura, A., Tanabe, K., Oe, N., Rai, S., Kon, S., Fukumoto, M., Takei, K., Abe, T., et al. (2012). The clathrin assembly protein PICALM is required for erythroid maturation and transferrin internalization in mice. PLoS ONE *7*, e31854.

Taylor, M.J., Perrais, D., and Merrifield, C.J. (2011). A high precision survey of the molecular dynamics of mammalian clathrin-mediated endocytosis. PLoS Biol. *9*, e1000604.

Taylor, M.J., Lampe, M., and Merrifield, C.J. (2012). A feedback loop between dynamin and actin recruitment during clathrin-mediated endocytosis. PLoS Biol. *10*, e1001302.

Tebar, F., Bohlander, S.K., and Sorkin, A. (1999). Clathrin assembly lymphoid myeloid leukemia (CALM) protein: localization in endocytic-coated pits, interactions with clathrin, and the impact of overexpression on clathrin-mediated traffic. Mol. Biol. Cell *10*, 2687–2702.

Traub, L.M. (2011). Regarding the amazing choreography of clathrin coats. PLoS Biol. *9*, e1001037.

Umasankar, P.K., Sanker, S., Thieman, J.R., Chakraborty, S., Wendland, B., Tsang, M., and Traub, L.M. (2012). Distinct and separable activities of the endocytic clathrin-coat components Fcho1/2 and AP-2 in developmental patterning. Nat. Cell Biol. *14*, 488–501.

Umasankar, P.K., Ma, L., Thieman, J.R., Jha, A., Doray, B., Watkins, S.C., and Traub, L.M. (2014). A clathrin coat assembly role for the muniscin protein central linker revealed by TALEN-mediated gene editing. eLife *3*, 3.

van Weering, J.R., Verkade, P., and Cullen, P.J. (2012). SNX-BAR-mediated endosome tubulation is co-ordinated with endosome maturation. Traffic *13*, 94–107.

Zhang, B., Koh, Y.H., Beckstead, R.B., Budnik, V., Ganetzky, B., and Bellen, H.J. (1998). Synaptic vesicle size and number are regulated by a clathrin adaptor protein required for endocytosis. Neuron *21*, 1465–1475.

Developmental Cell
Supplemental Information

CALM Regulates Clathrin-Coated Vesicle Size and Maturation by Directly Sensing and Driving Membrane Curvature

Sharon E. Miller, Signe Mathiasen, Nicholas A. Bright, Fabienne Pierre, Bernard T. Kelly, Nikolay Kladt, Astrid Schauss, Christien J. Merrifield, Dimitrios Stamou, Stefan Höning, and David J. Owen

Supplementary Figure 1 linked to Figure 1

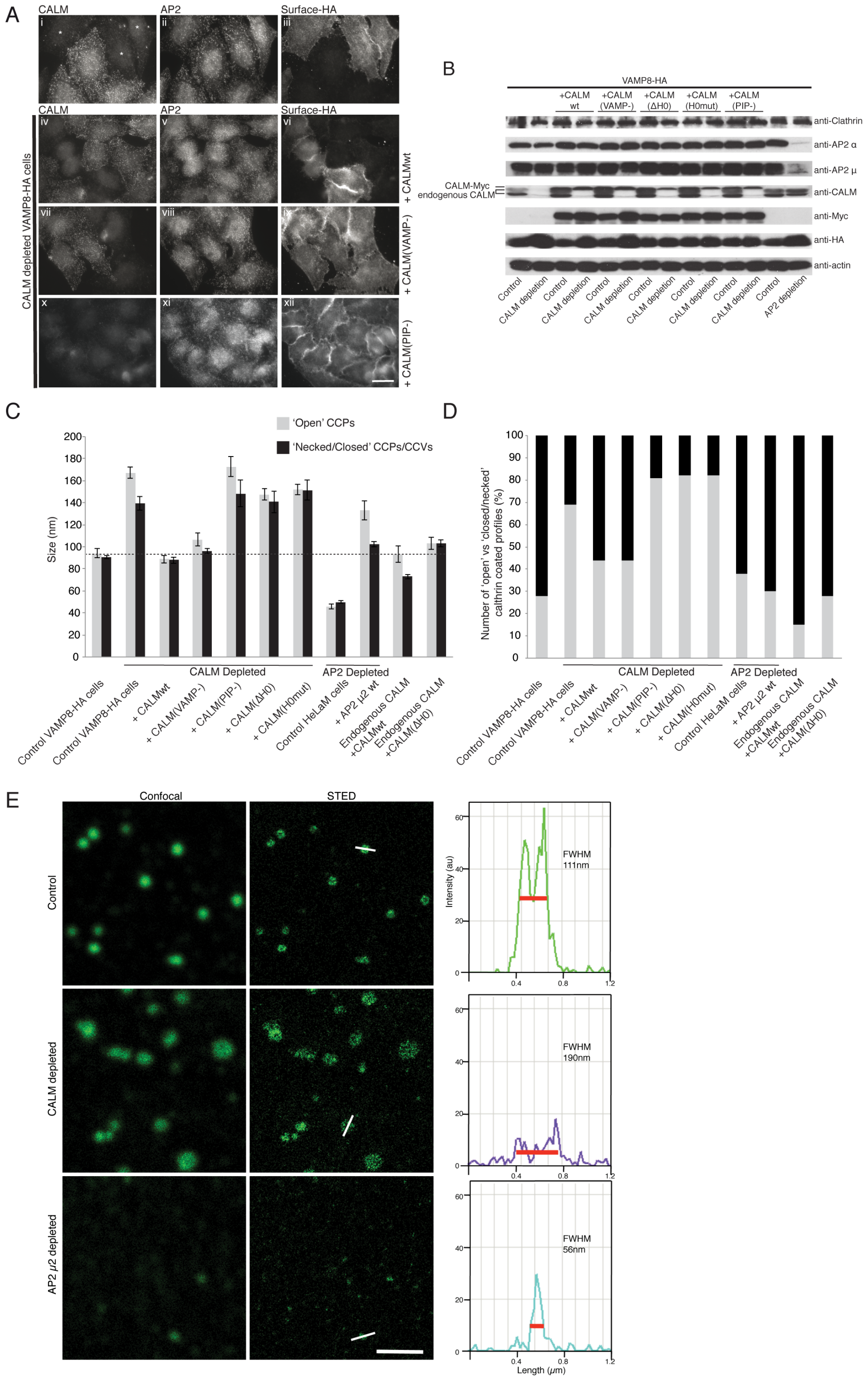

(A) All cells express VAMP8-HA and were labelled for CALM, AP2 and anti-HA (VAMP8-HA). (i-iii) Cells depleted of endogenous CALM (marked with *) were mixed with non CALM-depleted control cells. All other images (iv- xii) show cells depleted of endogenous CALM, and those with CALM positive punctae express full-length human siRNA-resistant CALM variants. CALM depletion or CALM add back variants do not apparently affect the localisation of AP2 (ii,v,viii,xi). In control cells VAMP8-HA is internalised by CALM but remains trapped on the plasma membrane when CALM is depleted (iii). In CALMwt rescue cells (iv-vi) CALM co-localises with AP2 punctae and VAMP8-HA is internalized. (vii-ix) CALM (VAMP-) add back cells show CALM and AP2 colocalisation, but this SNARE binding mutant does not endocytose VAMP8. (x-xii) CALM(PIP-) add back cells do not endocytose VAMP8 due to the inability of the construct to bind PtdIns4,5P_2 and therefore localise to the plasma membrane. Scale bar represents 20μm. (B) Western blots of cell lines showing the specificity of the CALM and AP2 μ2-subunit depletion. Extracts of cells expressing VAMP8-HA alone or expressing VAMP8-HA and myc-tagged siRNA-resistant CALMwt, CALM(VAMP-), CALM(ΔH0), CALM(H0mut) or CALM(PIP-) were probed with anti-clathrin, anti-AP2 α-subunit, anti-AP2 μ2-subunit, anti-CALM, anti-Myc, anti-HA or anti-actin (loading control). The blots show that each myc-tagged CALM construct is expressed at a similar level to one another and to endogenous CALM. In addition the CALM depletion specifically depletes the endogenous CALM but not the si-RNA resistant CALM. The specificity of the AP2 knockdown is also shown. Depletion of either of the major components in CCVs does not affect the level of the other. (C) Bar graph showing the average size of 'open' CCPs and 'necked'/'closed' CCPs/CCVs for control cells, CALM depleted cells

without or with re-expression of various CALM constructs. AP2 depletion and rescue is also represented as well as the overexpression of CALMwt and CALM(ΔH0). The width of 'open' profiles is measured at an average half depth of the CCP and shown in grey. The diameter of CCVs is averaged from two perpendicular measurements of the vesicle and is shown in black. The dotted line shows the average control size of CCPs/CCVs. Cells deleted of CALM without or with expression of CALM(PIP-), CALM(ΔH0) and CALM(H0mut) show a large increase in CCP/CCV size (~1.6x). Cells overexpressing CALMwt show a decrease in CCV size (~1.3x) not observed for overexpression of CALM(ΔH0) . In addition clathrin coated profiles of cells depleted of AP2 are ~2x smaller than those found in control cells. Error bars represent the standard error of >100 measurements). (D) A bar graph showing the number of 'open' CCPs (grey) versus 'necked'/'closed' CCPs/CCVs (black) for each cell line. In control cells the ratio of 'open' to 'necked'/'closed' profiles is 30:70, this ratio is conserved in cells depleted of CALM and expressing CALMwt or CALM(VAMP-). The ratio is reversed to 70:30 in CALM depleted cells and in those cell lines expressing CALM(PIP-), CALM(ΔH0) and CALM(H0mut) after CALM depletion. In those cells overexpressing CALMwt, there is an increase in the number of 'necked'/'closed' structures compared to 'open' (85:15) indicating an increase in CALM expression increases the number of CCVs, and therefore contributes to increased curvature and subsequent closure/budding of CCVs. (E) Clathrin-stained CCPs/CCVs were imaged by confocal and STED microscopy. The resulting STED image was then used to measure the diameter of the coated structures using the full-width at half maximum method. An example of one such measurement is shown for control, CALM depleted and AP2 μ2 depleted cells (graphs). Scale bar represents 0.5 μm

Supplementary Figure 2 linked to Figure 2

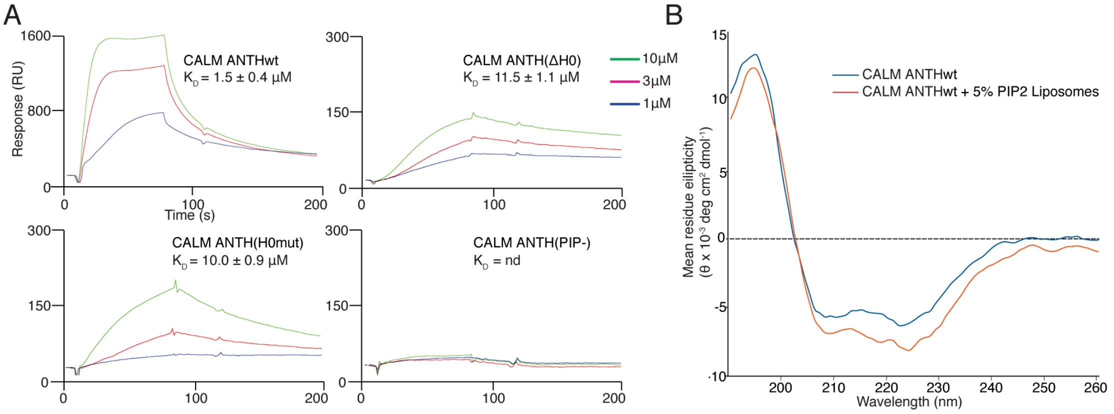

(A) Representative sensorgrams of liposome-based SPR traces for wt and mutant forms of CALM binding to PtdIns4,5P$_2$-containing liposomes at protein concentrations of 10μM 3μM and 1μM. (B) CD traces of CALM ANTHwt domain at the same concentration in the presence (red line) and absence (blue line) of PtdIns4,5P$_2$-containing liposomes. In the presence of liposomes there is a clear increase in α-helical content, indicated by the markedly enhanced features at both 222nm and 208nm.

Supplementary Figure 3 linked to Figure 3

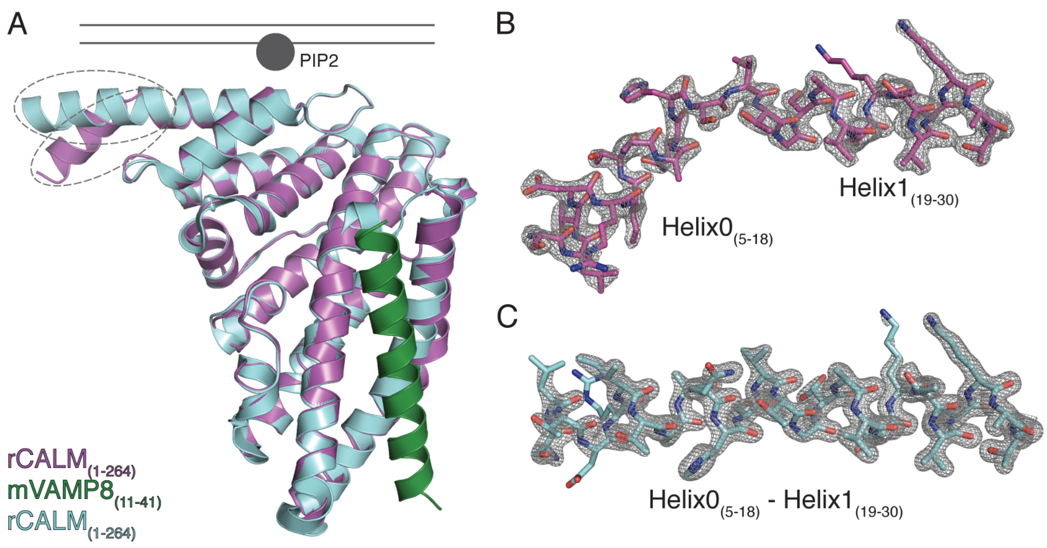

(A) Overlay of the ribbon representation of the CALM ANTH$_{(1-264)}$ (blue) and CALM ANTH:VAMP (purple/green) structures. (B) The electron density of the N-terminus of CALM in the CALM ANTH:VAMP structure at 2Å resolution and (C) the same region in the CALM ANTH$_{(1-264)}$ structure at 1.7Å resolution.

Supplementary Figure 4 linked to Figure 4

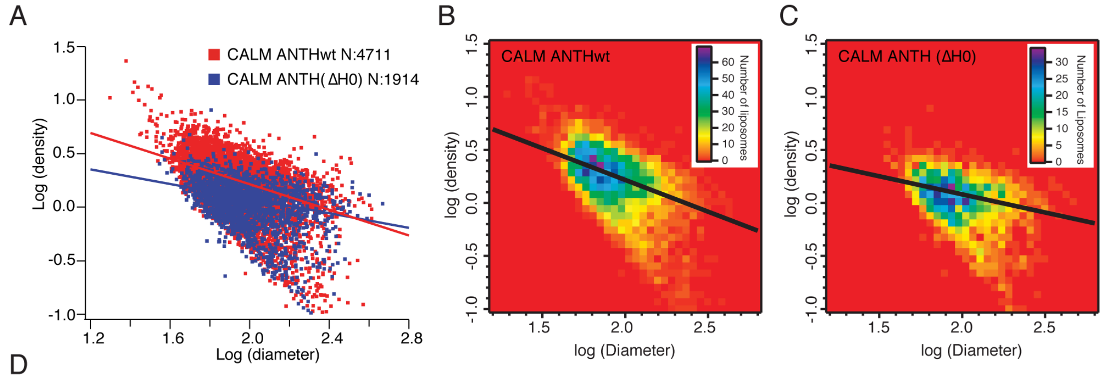

D

	Linear fitting slope ± SD
CALM ANTHwt	-0.64 ± 0.04
CALM ANTH(ΔH0)	-0.34 ± 0.04

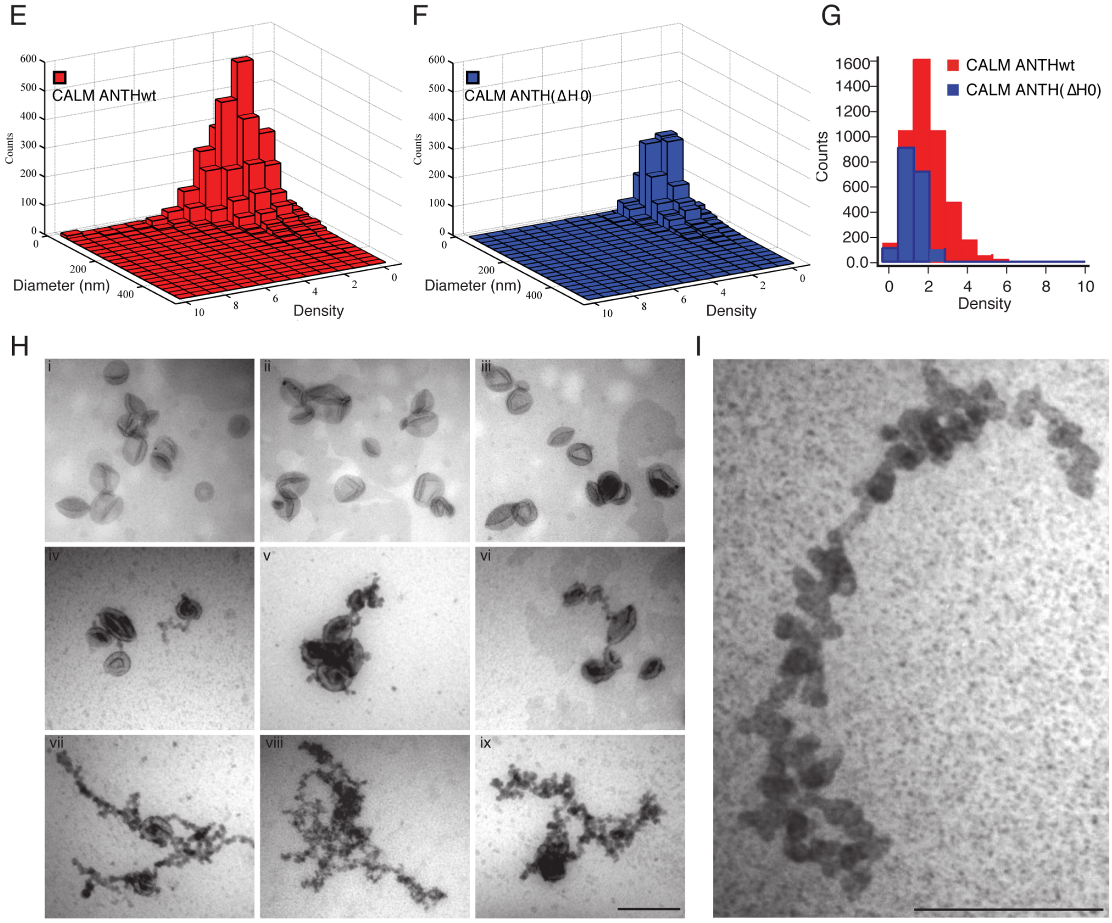

(A) Log-log representation of the data presented in Figure 4A. Because membrane recruitment follows a power law, the recruitment potency can be described by the slope of a straight-line fit to log-log representation of protein density versus liposome diameter. Red line, straight line fit to CALM ANTHwt

dataset. Blue line, straight-line fit to CALM ANTH(ΔH0) dataset. (B-C) CALM ANTHwt and CALM ANTH(ΔH0) heat map of the log-log representation in A. Colour scale, number of single liposomes. Black lines, CALM ANTHwt and CALM ANTH(ΔH0) straight-line fits from A. Both fits clearly follow the bins with the highest number of liposomes, demonstrating that the most frequently measured densities dictate the linear fits. (D) Average slope of straight-line fit (as in A) and standard deviation obtained from three independent measurements of CALM ANTHwt and CALM ANTH(ΔH0) to PtdIns4,5P_2 containing liposomes. (E-F) Bivariate histograms of CALM ANTHwt and CALM ANTH(**Δ**H0) density versus liposome diameter. The histograms reveal the sampling frequency and distribution of single liposome protein densities for CALM ANTHwt and CALM ANTH(**Δ**H0). The two protein samples display distinct density distributions, with CALM ANTHwt populating markedly higher densities that CALM ANTH(**Δ**H0). (G) Marginal histograms of the collapsed density histograms data in E and F. A comparison of the two histograms reveal that CALM ANTHwt and CALM ANTH(ΔH0) populate markedly different densities. (H) Typical examples of *in vitro* liposome tubulation with CALM ANTHwt. Control liposomes (i-iii) do not show any tubulation. (iv-vi) Liposomes that have been categorised as exhibiting slight tubulation are sometimes seen when incubated with 0.5μM CALM ANTHwt for one minute. More often liposome tubulation seen when incubated with 0.5μM CALM ANTHwt for one minute is 'pearlised' (vii-ix) and classified as extensive. Scale bar represents 500nm. (I) Zoom of the typical 'pearlised' tubulation by CALM ANTHwt. Scale bar represents 500nm.

Supplementary Figure 5 linked to Figure 5

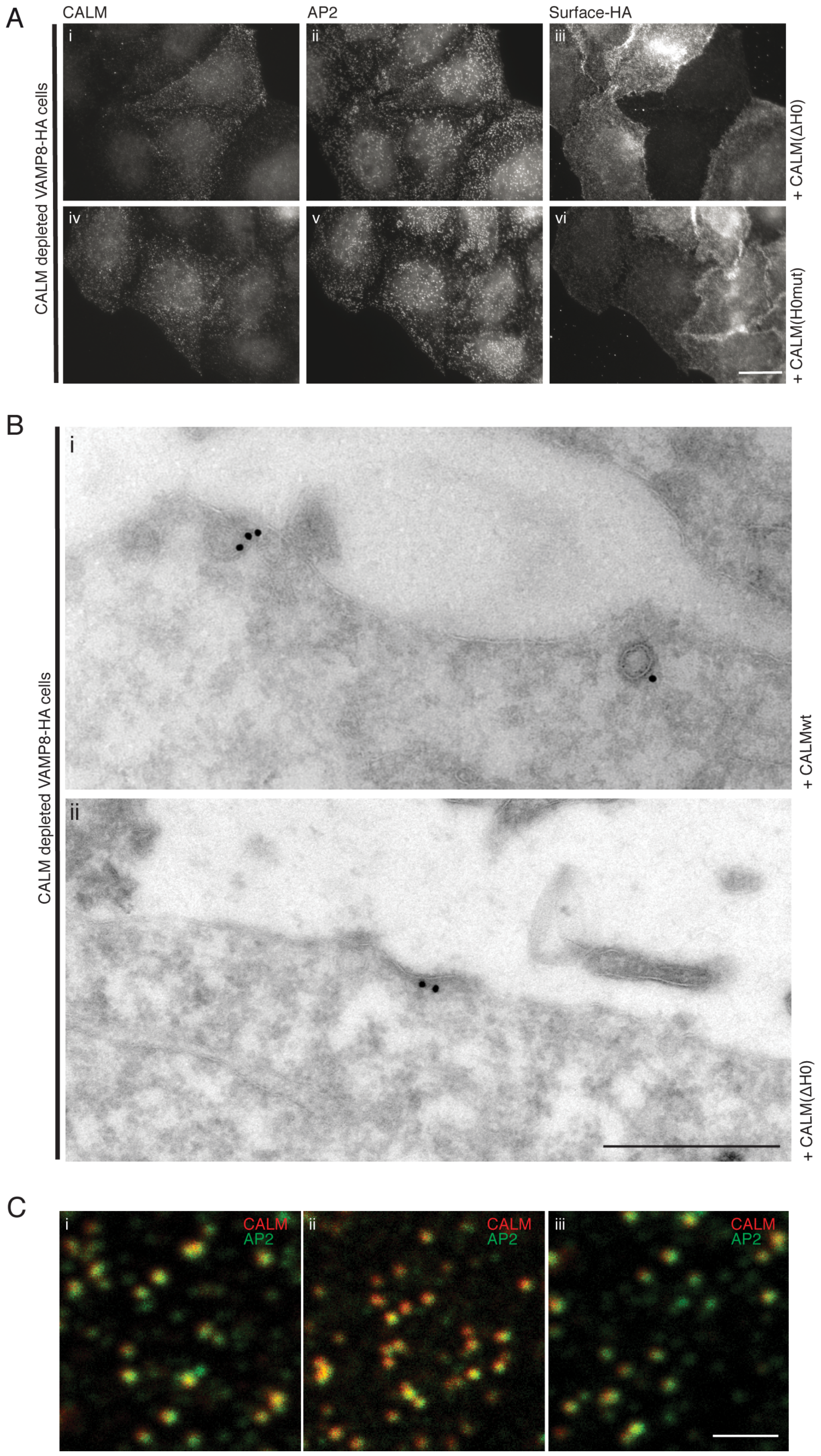

(A) All cells express an HA-tagged version of the endocytic SNARE VAMP8. For immunofluorescence microscopy cells were labelled for CALM, AP2 and anti-HA (VAMP8-HA). All cells were depleted of endogenous CALM, those cells with CALM punctae stably express full length human siRNA-resistant CALM(ΔH0) (i-iii) or CALM(H0mut) (iv-vi). CALM depletion without or with re-expression does not affect the localisation of AP2 (ii, v). When CALM is absent VAMP8-HA is trapped on the plasma membrane (iii, vi). CALMwt, CALM(ΔH0) and CALM(H0mut) co-localise with AP2 positive punctae (ii, v) and support internalisation of VAMP8-HA (iii, vi). Scale bar represents 20μm.

(B) Immunogold labelling of (i) myc-tagged CALMwt and (ii) CALM(ΔH0) reveal that the epitope-tagged constructs are correctly targeted to clathrin-coated structures in CALM depleted cells. Scale bar represents 200nm. (C) The co-localisation of AP2 (green) and CALM (red) punctae is shown in (i) control cells (ii) CALM depleted cells expressing CALM ANTHwt and (iii) CALM depleted cells expressing CALM(ΔH0). Scale bar represents 1μm.

Supplementary Table1 – linked to Figure 3.

Data collection and refinement statistics

Numbers in parentheses refer to the appropriate outer shell

	**$CALM_{ANTH(1\text{-}264)}$**	**$CALM_{ANTH(1\text{-}264)}$:$VAMP8_{(11\text{-}41)}$**
**Data collection**		
Beamline	Diamond I03	Diamond I03
Detector	ADSC Q315 CCD	ADSC Q315 CCD
Wavelength (Å)	0.9763	0.9763
Resolution limits (Å)	60.5–1.7 (1.74–1.70)	91.1–2.0 (2.08–2.03)
Space group	*C*2	*C*2
Unit cell dimensions (Å, °)	*a* = 95.9<br>*b* = 121.1<br>*c* = 62.4<br>ß = 110.7	*a* = 161.1<br>*b* = 100.3<br>*c* = 104.0<br>ß = 118.9
Unique reflections	72,568 (5,397)	93,133 (6,910)
Redundancy	4.3 (4.3)	3.7 (3.7)
Completeness (%)	99.4 (99.9)	99.7 (99.8)
<*I*/s*I*)>	9.8 (2.1)	10.0 (1.9)
R_{merge}[a]	0.071 (0.484)	0.079 (0.731)
R_{meas}[b]	0.081 (0.553)	0.093 (0.855)
R_{pim}[c]	0.038 (0.264)	0.048 (0.440)
**Refinement**		
Resolution limits (Å)	35.5–1.7 (1.72–1.70)	45.6–2.0 (2.05–2.03)
Reflections in working set	72,462 (2,670)	93,106 (2,933)
Reflections in test set	3,652 (138)	4,667 (162)
R_{work} (%)[d]	0.170 (0.288)	0.182 (0.294)
R_{free} (%)[e]	0.191 (0.300)	0.199 (0.365)
Number of molecules/complexes per asymmetric unit	2	3
Number of atoms (protein/water/other[f])	4,225/444/0	6,844/473/8
Residues in Ramachandran favored region (%)	98.3	99.2
Ramachandran outliers (%)	0.0	0.0
r.m.s.d bond lengths (Å)[g]	0.010	0.008
r.m.s.d bond angles (°)[g]	1.110	0.911
Average *B* factors (Å^2) (protein/solvent)	31.1/40.68	41.2/46.8

[a]$R_{merge} = \Sigma_{hkl} \Sigma_i |I(hkl;i) - \langle I(hkl)\rangle| / \Sigma_{hkl} \Sigma_i I(hkl;i)$, where *I(hkl;i)* is the intensity of an individual measurement of a reflection and <*I(hkl)*> is the average intensity of that reflection

[b]R_{meas} is the redundancy-independent merging *R* factor (Diederichs and Karplus, 1997)

[c]R_{pim} is the precision-indicating merging *R* factor (Weiss et al., 1998)

[d]$R_{work} = \Sigma_{hkl}||F_{obs}| - |F_{calc}||/\Sigma_{hkl} |F_{obs}|$, where $|F_{obs}|$ and $|F_{calc}|$ are the observed and calculated structure factor amplitudes, respectively

[e]R_{free} equals *R* of the cross-validation (test) set, 5% of the data removed prior to refinement

[f]Other indicates PO_4^{3-} ions

[g]r.m.s.d. is root mean square deviation from ideal geometry

Supplementary Materials and Methods

Constructs used in this study

pBMN-CALM (human) and mutants thereof, pBMN-CALM Met244Lys, pBMN-CALM ΔH0 $_{(19\text{-}652)}$, pBMN-CALM$_{(1\text{-}652)}$ Leu6Ser Ile10Ser Val17Ser and pBMN-CALM$_{(1\text{-}652)}$ Lys28Glu Lys38Glu Lys40Glu. pGEX-4T2 CALM ANTH$_{(1\text{-}289)}$ (rat) and mutants thereof, pGEX-4T2 CALM ANTH$_{(20\text{-}289)}$ (ΔH0), pGEX-4T2 CALM ANTH$_{(1\text{-}289)}$ Met244Lys (VAMP-), pGEX-4T2 CALM ANTH$_{(1\text{-}289)}$ Leu6Ser Ile10Ser Val17Ser (H0mut), pGEX-4T2 CALM ANTH$_{(1\text{-}289)}$ Lys28Glu Lys38Glu Lys40Glu (PIP-), pGEX-4T2 CALM ANTH Cys27Ser + C-Term CysGlyCys tag and pGEX-4T2 CALM ANTH(ΔH0) Cys27Ser + C-Term CysGlyCys tag. pCALM-GFP and pμ2-mCherry (Taylor et al., 2011)

Antibodies and ligands used in this study

Polyclonal rabbit anti-clathrin (gift of Margaret Robinson), polyclonal goat anti-CALM (Santa Cruz), polyclonal rabbit anti-AP2 β2 (gift of Margaret Robinson), monoclonal mouse anti-AP2 α subunit (Abcam), monoclonal mouse anti-AP2 μ2-subunit (BD Transduction Laboratories), monoclonal mouse anti-Myc (Millipore), monoclonal mouse anti-HA (Covance) and monoclonal mouse anti-actin (Abcam). Secondary antibodies appropriate to primary antibodies as well as EGF-Alexa488 and Tf-Alexa565 as IF probes for endocytosis via their respective receptors were all from Invitrogen.

Western blotting

Levels of protein expression and depletion efficiency were analysed by western blots probed with appropriate antibodies.

Protein expression and purification

All recombinant proteins were expressed in BL21(DE3) pLysS cells for 16hr at 22°C after induction with 0.2mM IPTG at 37°C. Following lysis, all GST-CALM fusion proteins were purified on Glutathione Sepharose 4B in 20mM Tris pH 7.4, 170mM NaCl, 4mM DTT. The N-terminal GST tag was cleaved with thrombin overnight at 20°C. The cleaved proteins were then eluted and the thrombin cleavage was halted by the addition of AEBSF. All proteins were then further purified by S200 gel filtration in 20mM Tris pH 7.4, 170mM NaCl, 0.5mM TCEP.

Surface plasmon resonance (SPR) based analysis of membrane binding

Liposomes for SPR studies were composed of synthetic 1-palmitoyl-2-oleoyl-sn-glycero-3-phosphocholine (POPC), 1-palmitoyl-2-oleoyl-sn-glycero-3-phosphoethanolamine (POPE) and PtdIns4,5P_2 at a ratio of 85%:20%:5%. Liposomes (300μM final concentration) were captured on the four flow cells of a L1 surface in 5min injections at a flow rate of 10μl/min using 20mM Tris pH 7.4, 170mM NaCl, 0.5mM TCEP as buffer. Subsequently the flow rate was set to 30μl/min and the surfaces were stabilized with two injections of 50mM NaOH for 10sec. Proteins were then injected for 60sec followed by a dissociation period of 120sec. Protein that remained bound to liposomes was removed by a 5sec pulse injection of NaOH. Such an experimental cycle was repeated at least five times with different concentrations ranging from 0.5μM–20μM. The resulting sensorgrams were used to calculate the K_D values after background

subtraction (binding to PC/PE liposomes) using the manufacturers evaluation software (version 4.1.1) assuming a 1:1 interaction.

Calculating % coverage of pearlised PtdIns4,5P_2 containing liposomes by CALM

In our liposome tubulation system we can estimate the % membrane coverage of the CALM ANTH that results in the production of endocytic CCV sized 'pearlised' tubes. We estimate the % coverage of CALM to be ~16%, close to the value of ~30% calculated for the surface occupation by epsin proposed to generate narrower flat-sided tubes from synthetic liposomes (Kozlov et al., 2014)

For this calculation we have had to make some assumptions similar to those used in (Kozlov et al., 2014) such as that projecting the structure down onto the membrane will give a representative protein footprint. Our value may be an underestimation of the amount of CALM ANTH surrounding the 'pearls' as the affinity of CALM for the membrane will increase as curvature ie pearlising increases since CALM shows increased/preferred binding to membranes of <100nm curvature (see data in Figure 4). Further PtdIns4,5P_2 local concentrations may be higher than the 2.5μM overall estimate as it has been shown that PtdIns4,5P_2 tends to self aggregate into patches (Stachowiak et al., 2012)

CALM concentration 0.5μM and PtdIns4,5P_2 concentration estimated at 2.5μM (from PtdIns4,5P_2 already present in brain lipid extract from Avanti and

supplemented).

and

K_D for 200nm liposomes 1.5μM at 170mM salt used in tubulation experiments derived by SPR

Solving resulting quadratic equation, concentration of CALM on membrane 0.3μM (probably an underestimate see above)

Assuming an overall lipid concentration of 100uM based on the amount of lipids used and an average MW of 1000Da, there are ~6 x 10^{19} lipid headgroups present in a reaction.

From the membrane model (Heller et al., 1993) there are ~900 lipid headgroups packed in a square 262Å x 262Å so the total amount of lipid gives a total lipid surface area of 2.3 x 10^{21} Å^2

Each CALM molecule has a footprint of ~2000Å^2 and as the concentration of bound CALM is 0.3μM there will be 1.8 x 10^{17} molecules of CALM bound which will take up ~3.6 x 10^{20} Å^2.

So fraction of membrane surface covered by CALM is 3.6x10^{20} / 2.3x10^{21} = ~16%

Calculation of CALM coverage on endocytic CCVs

CALM membrane footprint derived from structure ~2000Å2

AP2 membrane footprint derived from structure ~8500Å2

Surrounding a 50nm (500Å) diameter vesicle (Surface area 775000Å2) there are 60 clathrin triskelia and by quantitative proteomic data (Borner et al., 2012) around 60AP2s (SA ~510000Å2) and 60-70 CALMs (SA~130000Å2).

Hence around 17% of an endocytic vesicle membrane is covered by CALM.

We now refer briefly to this value in the discussion section of the paper, although as with any modeling calculations there will undoubtedly be assumptions made that are not entirely valid, especially in vivo where membrane composition is complicated and other factors are at work. Also it should be noted that this value will be much higher than the value for surface coverage of endocytic CCVs by epsin *in vivo* as quantitative proteomics (Borner et al., 2012) indicates that there is little epsin (<1% of the adaptor complement) in endocytic CCVs.

Circular dichroism

Two identical 0.5ml samples of 1mg/ml CALM in 50mM sodium phosphate pH7.4 were added to either 500μl 50mM sodium phosphate pH7.4 or 500μl BPLE 5% PtdIns4,$5P_2$ liposomes that had been resuspended in 50mM sodium phosphate pH7.4. CD spectra were collected in a Jasco J-810 and 20 runs averaged using manufacturer's software. Buffer only or buffer+liposome background traces were subtracted as appropriate and the resulting spectra

overlayed in Savitsky-Golay convolution width 15. All experiments were repeated at least 4 times.

Single liposome curvature (SLiC) assay

Materials

1,2-Dioleoyl-sn-Glycero-3-Phosphocholine (DOPC); 1,2-Dioleoyl-sn-Glycero-3-Phosphatidylethanolamine-N-(cap biotinyl) (DOPE-Biotin); 1,2-Dioleoyl-sn-Glycero-3-Phosphoethanolamine (DOPE); 1,2-dioleoyl-*sn*-glycero-3-phospho-(1'-myo-inositol-4',5'-bisphosphate) (DOPE-PI(4,5)P_2) were all acquired from Avanti Polar Lipids Inc (Alabaster, AL). 1,2-Dioleoyl-sn-Glycero-3-Phosphatidylethanolamine-Atto655 (DOPE-Atto655) was from Atto-Tec. Liposomes for SLiC were prepared using a previously described lipid hydration method (Bhatia et al., 2009; Hatzakis et al., 2009). In brief, lipids dissolved in chloroform were mixed in a glass vial, at the molar ratios DOPC;DOPE;DOPE-biotin;DOPE-Atto655;DOPE-PI(4,5)P_2 (69.3;20;0.2;0.5;10). The solution was dried under nitrogen flow and incubated in vacuum for 4hr. Liposomes were rehydrated by adding a 290mM D-Sorbitol solution to the lipid film, for a final lipid concentration of 0.5g/l. The mixture was incubated overnight at room temperature. The liposomes were then subjected to 20 freeze-thaw cycles to minimize multilamellarity and extruded once through a single polycarbonate filter with a pore size of 400nm in pressurized extruder (Avanti Polar Lipids Inc). The liposomes where flash-frozen in liquid nitrogen and stored at -21°C.

Labelling of CALM ANTH proteins

Alexa Fluor 488 C_5 maleimide (Life Technologies) was dissolved at 50mg/ml in DMSO and added dropwise to give a twofold molar excess over 2.8mg/ml of CALM ANTHwt or CALM ANTH(ΔH0) in 20mM Tris pH 7.4, 170mM NaCl, 0.5mM TCEP. The reaction was incubated for 2hr at 20°C with continuous gentle inversion (~10rpm) in a foil-wrapped tube, quenched with the addition of 4mM DTT, dialyzed against 20mM Tris pH 7.4, 170mM NaCl, 4mM DTT to remove the bulk of the unincorporated dye, and, finally, purified by size exclusion chromatography on an S200 column (equilibrated in 20mM Tris pH 7.4, 170mM NaCl, 4mM DTT) in the dark.

Functionalization of microscope glass surfaces

Microscope chamber parts and glass slides were cleaned according to previously published procedures (Christensen et al., 2012). Glass slides were dried in nitrogen, plasma etched for 120sec., mounted in a microscope chamber and incubated with a mixture of 1000:6 PLL-g-PEG:PLL-g-PEG-Biotin (SuSoS AG, Switzerland) (1g/l) in surface buffer (15mM HEPES pH 5.6) for 30min. After consecutive washing steps x10 with buffer (10mM Tris pH 7.4, 170mM NaCl, 0.05mM TCEP) the passivated surfaces were incubated with 0.1g/l Neutravidin in surface buffer for 10min, followed by additional washing x10.

Liposome size calibration

We previously described a methodology for calibrating liposome diameter by combining confocal fluorescence microscopy and dynamic light scattering (DLS) measurements (Kunding et al., 2008). In brief, the integrated intensity of a liposome labelled in its membrane (here by DOPE-Atto 655) is proportional to

the square of its diameter. In brief, the integrated intensity of a liposome labeled in its membrane (here by DOPE-Atto 655) is proportional to the total number of lipids in the liposome, therefore the total liposome surface area, and the square of its diameter assuming a spherical shape. We were able to confirm that liposomes with diameters below the diffraction limited retained a spherical shape upon tethering on a surface using fluorescence energy transfer at the contact area between liposome and surface (Bendix et al., 2009). A conversion from liposome membrane intensity to physical size was therefore possible using a calibration sample of extruded small unilamellar vesicles with a controlled and narrow size distribution whose mean size was obtained by DLS (Kunding et al., 2008).

Data analysis, log-log representation and quantification of curvature sensing abilities

Images were acquired on a Leica TCS-SP5 inverted confocal microscope using a HCX PL APO oil immersion objective (100x magnification, 1.46 NA) (Leica, Wetzlar, Germany). CALM ANTH-Alexa488 and DOPE-Atto655 were excited by a 488nm and 655nm laserline, and emissions were filtered out in the range of 498nm – 600nm and 643nm – 750nm respectively. Images were acquired in the format 1024x1024 pixels, each pixel corresponding to 50.5nm sample length, bit-depth of 16, and a scanspeed of 400Hz. All experiments were performed in a 10mM Tris pH 7.4, 170mM NaCl, 0.05mM TCEP buffer. For all samples, liposomes were imaged before the addition of protein to ensure that the binding molecule did not affect liposome morphology or fluorescence.

Images were analyzed using a specifically developed routine in Igor Pro 5.05 (Wavemetrics) for extracting single particle positions, background

corrected integrated fluorescence intensities and identifying co-localised protein and liposome signals, as described previously (Hatzakis et al., 2009). We previously showed that recruitment by membrane curvature follows a power law, and quantified the recruitment potency as the slope of a straight-line fit to a log-log representation of protein density vs. liposome diameter. In this representation of the data a more negative slope represents a stronger recruitment by membrane curvature.

Transmission electron microscopy

Cells were fixed by addition of 5% glutaraldehyde (GA) / 4% paraformaldehyde (PFA) in 0.1M sodium cacodylate, pH 7.2 to an equal volume of tissue culture medium for 2min at 37°C. This solution was replaced with 2.5% GA / 2% PFA in 0.1M sodium cacodylate buffer, pH 7.2, and the cells were scraped from the dish, pelleted and fixed for 1hr at 20°C. The cell pellet was washed with 0.1M sodium cacodylate buffer, pH 7.2, and post-fixed with 1% osmium tetroxide in 0.1M sodium cacodylate buffer, pH 7.2, for 1hr. The pellet was washed with 0.05M sodium maleate buffer, pH 5.2, and stained *en bloc* with 0.5% uranyl acetate in 0.05M sodium maleate buffer for 1hr. The cell pellets were dehydrated in ethanol, exchanged into 1,2-epoxy propane and embedded in Araldite CY212 resin (Agar Scientific). Ultrathin sections were cut using a diamond knife mounted on an ultramicrotome (Reichert Ultracut S; Leica), collected onto formvar/carbon-coated EM grids and stained with uranyl acetate and lead citrate (Reynolds, 1963). The sections were observed in a CM 100 TEM (Philips) at an operating voltage of 80kV. For analysis of clathrin coated profiles, cell pellets were randomly sectioned and oriented in the electron microscope. Grids

were systematically scanned and on average 150 images of each cell line were captured using a CCD camera (Megaview III; Olympus). These images were used to trace 100 individual clathrin coated profiles with a Wacom® Bamboo™ tablet and overlayed in Adobe® Illustrator® CS6.

Immunogold electron microscopy

Cells were fixed by addition of 8% PFA / 0.2% gluteraldehyde in 0.1M sodium cacodylate buffer, pH 7.2 to an equal volume of cell culture medium at 37°C. After 2min, the solution was removed and cells were fixed in 4% PFA/0.1% glutaraldehyde in 0.1M Sodium Cacodylate buffer, pH 7.2, for 1hr at 20°C. Cells were scraped, pelleted, embedded in 10% gelatin at 37°C, cooled on ice and infused with 1.7M sucrose/15% polyvinyl pyrolidone overnight at 4°C. The blocks were then frozen onto aluminium stubs in liquid nitrogen and ultrathin frozen sections were cut with a diamond knife in a cryo ultramicrotome (Ultracut UCT /EM FCS; Leica), collected with 1% methyl cellulose / 1.15M sucrose and mounted on formvar/carbon-coated TEM grids. Sections were immunolabelled with an anti-myc antibody (Upstate) followed by rabbit anti-mouse (DAKO) and protein A: 15nm colloidal gold (Utrecht University, The Netherlands). Images were captured using a CCD camera (Eagle 4K) on a Tecnai G2 Spirit BioTWIN TEM (FEI, Eindhoven) at an operating voltage of 80kV.

Immunofluorescence Imaging

Conventional immunofluorescence microscopy was performed with an Axioplan fluorescence microscope (Carl Zeiss Ltd) equipped with a 63x oil immersion objective. Images were captured with a Hamamatsu Orca-R2 C10600 camera

(Hamamatsu Photonics). Confocal laser scanning microscopy was performed with a Leica TCS SP8 (Leica microsystems) and a 100x oil immersion objective. Images displayed in figure S5 represent single focal planes. The uptake of EGF-Alexa488 and Tf-Alexa565 as displayed in Figure 6 was analyzed in the total volume of the imaged cells by using z-stacks with 10 individual sections that covered a total thickness of 5-6µm. Subsequently, all sections were superimposed into one single maximum projection. To achieve optimal imaging, a frame averaging of 3 was used. No further image processing was applied.

STED (Stimulated Emission Depletion) super resolution microscopy.

Samples were prepared as described in main experimental procedures for standard immunofluorescent microscopy but to avoid non-specific staining due to high laser power excitation the nuclear staining was omitted. Prior to mounting, 0,2µm TetraSpeck fluorescent microspheres (Invitrogen) were diluted 1:500 in water, added to samples and incubated for 10 min. The spheres served as a size standard and as a control of fluorescence intensity among samples. For microscopy, a Leica TCS SP8 gSTED equipped with a 100x/1.4 Oil STED Orange lens and a 592 nm depletion laser was used. In brief, confocal and STED images were collected sequentially with a HyD detector at a scan speed of 400 lines/s, a pixel size of 20 nm (960x960 pixel), and a line average of 2. In the STED mode, a pulsed white light laser tuned to 488 nm was used for excitation and the emission signal was collected between 491 nm and 547 nm. To improve the lateral resolution gating between 0.6 ns and 6 ns was performed. The resulting images were imported into Photoshop (Adobe), adjusted equally for intensity

and background and mounted into the final figures without any further image correction.

For quantification, the STED images were first deconvolved with Huygens Professional 14.10 (Scientific Volume Imaging) using a theoretical PSF. The deconvolved STED image was then used to determine coated pit sizes. Coated pit size was quantified with custom code written in Python 2.7, using the scikit-image library (Stéfan van der Walt, Johannes L. Schönberger, Juan Nunez-Iglesias, François Boulogne, Joshua D. Warner, Neil Yager, Emmanuelle Gouillart, Tony Yu and the scikit-image contributors. scikit-image: Image processing in Python. PeerJ 2:e453 (2014) http://dx.doi.org/10.7717/peerj.453). In short, pits were first roughly detected with the Laplacian of Gaussian method (sigma and threshold values determined on control data and then kept constant). In the next step, a 2D circular Gaussian was fit on the pixel neighbourhood of each identified pit to determine its full width at half maximum (FWHM).

TIRF imaging of CALM-GFP and μ2-mCherry in live cells.

HEK293 cells were plated on clean coverslips on the same day as transfection and imaged 48hr later to allow several cell divisions and to give transfected cells time to adapt moderate protein overexpression (Taylor et al., 2012; Taylor et al., 2011). Only low expressing cells were imaged, the laser power at 488nm and 568nm and exposure times were fixed and only cells showing comparable similar signal to noise at coated pits for CALM-GFP and μ2-mCherry were analysed. To determine CCP maturation time in CALM knock-down cells HT1080 cells were transfected with either CALM (ACAGTTGGCAGACAGTTTA) or a non-targeting control siRNA followed by transfection with CALM-GFP and AP2 μ2-

mCherry. All image analysis used custom written scripts in Matlab (Taylor et al., 2011). To track fluorescently labelled clathrin coated pits the ‘a trous wavelet’ segmentation was used as described previously (Taylor et al., 2011) and track histories constructed using the nearest neighbour algorithm. To quantify the average dynamics of CALM-GFP and μ2-mCherry during clathrin coated pit nucleation the start of CALM-GFP track histories was used as a reference to align cohorts of fluorescence traces. This introduced a slight artefact (the ‘jump’ in the CALM-GFP fluorescence trace at t=0s, Figure 2A) since the first moment of detection of CALM-GFP will inevitably coincide with additive noise. However, the CALM-GFP and μ2-mCherry signals were well aligned for ~20sec preceding the first detection of CALM-GFP and there was no evidence to suggest that CALM-GFP arrived before or after μ2-Cherry at these time scales (1.0Hz) (the modal difference between first detection of CALM-GFP and μ2-mCherry was ‘0s’, 1963 events, 3 cells). The recruitment curves were well sampled and a faster acquisition rate would not have improved the analysis of clathrin coated pit nucleation. To align the fluorescence traces to the moment of budding the decrease in CALM-GFP was used as a reference. Briefly, the CALM-GFP fluorescence trace was convolved with a kernel adapted to detect the maximal decrease in fluorescence signal in CALM-GFP which was taken as ‘t = 0s’. Cohorts of CALM-GFP and μ2-mCherry fluorescence traces were then aligned and averaged.

References

Bendix, P.M., Pedersen, M.S., and Stamou, D. (2009). Quantification of nano-scale intermembrane contact areas by using fluorescence resonance energy transfer. P Natl Acad Sci USA *106*, 12341-12346.

Bhatia, V.K., Madsen, K.L., Bolinger, P.Y., Kunding, A., Hedegard, P., Gether, U., and Stamou, D. (2009). Amphipathic motifs in BAR domains are essential for membrane curvature sensing. EMBO J *28*, 3303-3314.

Borner, G.H.H., Antrobus, R., Hirst, J., Bhumbra, G.S., Kozik, P., Jackson, L.P., Sahlender, D.A., and Robinson, M.S. (2012). Multivariate proteomic profiling identifies novel accessory proteins of coated vesicles. J Cell Biol *197*, 141-160.

Christensen, S.M., Bolinger, P.Y., Hatzakis, N.S., Mortensen, M.W., and Stamou, D. (2012). Mixing subattolitre volumes in a quantitative and highly parallel manner with soft matter nanofluidics. Nat Nanotechnol *7*, 51-55.

Diederichs, K., and Karplus, P.A. (1997). Improved R-factors for diffraction data analysis in macromolecular crystallography. Nat Struct Biol *4*, 269-275.

Hatzakis, N.S., Bhatia, V.K., Larsen, J., Madsen, K.L., Bolinger, P.Y., Kunding, A.H., Castillo, J., Gether, U., Hedegard, P., and Stamou, D. (2009). How curved membranes recruit amphipathic helices and protein anchoring motifs. Nat Chem Biol *5*, 835-841.

Heller, H., Schaefer, M., and Schulten, K. (1993). Molecular-Dynamics Simulation of a Bilayer of 200 Lipids in the Gel and in the Liquid-Crystal Phases. J Phys Chem-Us *97*, 8343-8360.

Kozlov, M.M., Campelo, F., Liska, N., Chernomordik, L.V., Marrink, S.J., and McMahon, H.T. (2014). Mechanisms shaping cell membranes. Current opinion in cell biology *29*, 53-60.

Kunding, A.H., Mortensen, M.W., Christensen, S.M., and Stamou, D. (2008). A fluorescence-based technique to construct size distributions from single-object measurements: Application to the extrusion of lipid vesicles. Biophys J *95*, 1176-1188.

Reynolds, E.S. (1963). Use of Lead Citrate at High Ph as an Electron-Opaque Stain in Electron Microscopy. J Cell Biol *17*, 208-&.

Stachowiak, J.C., Schmid, E.M., Ryan, C.J., Ann, H.S., Sasaki, D.Y., Sherman, M.B., Geissler, P.L., Fletcher, D.A., and Hayden, C.C. (2012). Membrane bending by protein-protein crowding. Nat Cell Biol *14*, 944-+.

Taylor, M.J., Lampe, M., and Merrifield, C.J. (2012). A Feedback Loop between Dynamin and Actin Recruitment during Clathrin-Mediated Endocytosis. Plos Biology *10*.

Taylor, M.J., Perrais, D., and Merrifield, C.J. (2011). A high precision survey of the molecular dynamics of mammalian clathrin-mediated endocytosis. PLoS Biol *9*, e1000604.

Weiss, M.S., Metzner, H.J., and Hilgenfeld, R. (1998). Two non-proline cis peptide bonds may be important for factor XIII function. Febs Lett *423*, 291-296.
